# The DNA polymerase of bacteriophage YerA41 replicates its T-modified DNA in a primer-independent manner

**DOI:** 10.1093/nar/gkac203

**Published:** 2022-03-31

**Authors:** Miguel V Gomez-Raya-Vilanova, Katarzyna Leskinen, Arnab Bhattacharjee, Pasi Virta, Petja Rosenqvist, Jake L R Smith, Oliver W Bayfield, Christina Homberger, Tobias Kerrinnes, Jörg Vogel, Maria I Pajunen, Mikael Skurnik

**Affiliations:** Department of Bacteriology and Immunology, Medicum, Human Microbiome Research Program, Faculty of Medicine, University of Helsinki, 00014 UH, Helsinki, Finland; Department of Bacteriology and Immunology, Medicum, Human Microbiome Research Program, Faculty of Medicine, University of Helsinki, 00014 UH, Helsinki, Finland; Department of Bacteriology and Immunology, Medicum, Human Microbiome Research Program, Faculty of Medicine, University of Helsinki, 00014 UH, Helsinki, Finland; Drug Discovery, Herantis Pharma Ltd. Bertel Jungin Aukio 1, 02600 Espoo, Finland; Department of Chemistry, 20014 University of Turku, Turku, Finland; Department of Chemistry, 20014 University of Turku, Turku, Finland; York Structural Biology Laboratory, University of York, YO10 5DD York, United Kingdom; York Structural Biology Laboratory, University of York, YO10 5DD York, United Kingdom; Institute of Molecular Infection Biology (IMIB), University of Würzburg, D-97080 Würzburg, Germany; Helmholtz Institute for RNA-based Infection Research (HIRI), Helmholtz Centre for Infection Research (HZI), D-97080 Würzburg, Germany; Institute of Molecular Infection Biology (IMIB), University of Würzburg, D-97080 Würzburg, Germany; Helmholtz Institute for RNA-based Infection Research (HIRI), Helmholtz Centre for Infection Research (HZI), D-97080 Würzburg, Germany; Faculty of Medicine, University of Würzburg, D-97080 Würzburg, Germany; Department of Bacteriology and Immunology, Medicum, Human Microbiome Research Program, Faculty of Medicine, University of Helsinki, 00014 UH, Helsinki, Finland; Department of Bacteriology and Immunology, Medicum, Human Microbiome Research Program, Faculty of Medicine, University of Helsinki, 00014 UH, Helsinki, Finland; Division of Clinical Microbiology, HUSLAB, Helsinki University Hospital, 00290 Helsinki, Finland

## Abstract

*Yersinia* phage YerA41 is morphologically similar to jumbo bacteriophages. The isolated genomic material of YerA41 could not be digested by restriction enzymes, and used as a template by conventional DNA polymerases. Nucleoside analysis of the YerA41 genomic material, carried out to find out whether this was due to modified nucleotides, revealed the presence of a ca 1 kDa substitution of thymidine with apparent oligosaccharide character. We identified and purified the phage DNA polymerase (DNAP) that could replicate the YerA41 genomic DNA even without added primers. Cryo-electron microscopy (EM) was used to characterize structural details of the phage particle. The storage capacity of the 131 nm diameter head was calculated to accommodate a significantly longer genome than that of the 145 577 bp genomic DNA of YerA41 determined here. Indeed, cryo-EM revealed, in contrast to the 25 Å in other phages, spacings of 33–36 Å between shells of the genomic material inside YerA41 heads suggesting that the heavily substituted thymidine increases significantly the spacing of the DNA packaged inside the capsid. In conclusion, YerA41 appears to be an unconventional phage that packages thymidine-modified genomic DNA into its capsids along with its own DNAP that has the ability to replicate the genome.

## INTRODUCTION

Bacteriophages, or phages, are the most abundant organism on earth (1,2). They are viruses that, in order to replicate, target and kill bacteria. Phages are of primary interest in clinical applications as they can be used to treat bacterial infections (2–5). Moreover, these viruses act as a rich source for discovery of novel antimicrobial compounds. Studying their virulence mechanisms and proteins could lead to the identification of toxic compounds targeting bacteria (1).

Previously, numerous cellular organisms and viruses with variations of the canonical nucleotides in their DNA, such as methyl groups or amino acids, have been reported. These usually do not alter the specificity of base pairing and do not usually affect DNA replication (6,7). Bacteriophages contain the greatest diversity of modified bases observed in nature so far. The primary function of these modifications is to prevent cleavage by host restriction endonucleases (6,7).

YerA41 is a bacteriophage that was isolated due to its ability to infect *Yersinia ruckeri*, the causative agent of enteric red mouth disease in salmonid fish. It has a broad host range as it infects also strains of some other *Yersinia* species, *Escherichia coli*, *Shigella flexneri*, *Enterobacter cloacae*, *Klebsiella* and *Erwinia* (8,9). Multiple unsuccessful attempts to sequence the genome of this phage were carried out with conventional sequencing approaches and modern PCR-based NGS such as Illumina. In addition, the conventional PCR using commercially available DNA polymerases was unable to amplify its DNA and restriction enzymes were unable to digest it (8). RNA sequencing was used to sequence the RNA isolated from YerA41-infected *Y. ruckeri* bacteria resulting in nine YerA41-specific sequence scaffolds with a total length of 143 kb (8). Proteomic analysis of the phage particles revealed that also the predicted DNA polymerase was associated with the virion structure (8). It was hypothesized that the presence of some special modifications in the phage DNA renders it unsusceptible to restriction digestion and amplification by commercial polymerases.

Unlike commercially available DNA polymerases, the DNA polymerase coded by the phage's genome should be able to amplify its own modified DNA. When host polymerases are unable to replicate the viral genome, the bacteriophage produces its own polymerase that can function using the modified DNA as a template (10). Interestingly, this special polymerase should also be able to amplify DNA chains with modifications from other organisms making it a potential highly valuable tool. Despite the benefits of commercial DNA polymerases to use modified nucleotides as substrate, for example in Sanger and next generation sequencing, aptamer selection, and genotyping (11,12), such research has not been carried out with natural non-engineered DNA polymerases. Nevertheless, some commercially available DNA polymerases such as Therminator have been engineered to accept certain, but by no means, all modified nucleotides (11).

Here, we show that YerA41 has a genomic material containing exceptionally bulky thymidine substitution apparently preventing both the activity of restriction enzymes and conventional DNA polymerases. We expressed and purified DNAP01, which showed DNA polymerase activity and was able to use the YerA41 genomic material as a template without any added primers, with just added dNTPs. With the help of this highly unconventional DNAP we were able to complete the YerA41 genomic DNA sequence. Transmission electron microscopy of this unconventional phage revealed structural insights of the capsid structure.

## MATERIALS AND METHODS

### Reagents

In order to carry out the various experimental procedures successfully, media and solutions were prepared as indicated in Supplementary Table S1. For other commercial reagents and details, see the corresponding sub-sections below.

### Biological resources

Unless stated otherwise the bacterial strain used for expression of the protein used in this work is *E. coli* strain BL21 (DE3) that was cultured at +37°C in Lysogeny Broth (LB) liquid medium (13).

The *Y. ruckeri* phage YerA41 (9) was obtained from the Skurnik lab collection, and was propagated in *Y. ruckeri* strain RS41 (HER1300). RS41 was grown at room temperature (RT) using the double-agar method with LB agar plates and soft agar (Supplementary Table S1), or alternatively using the Gabrichevsky method described below.

### ‘Gabrichevsky’ method for phage propagation

The phage bottle lysates were made using this method. Briefly, 2% LB agar was poured into 175 cm^2^ cell culture bottles in such way that the agar formed a wedge that went from 1 cm at the dead-end of the bottle to 2 mm near the tap. The agar surface was flooded with 2.3 ml of o/n bacterial culture and the bacteria were grown on it for three and a half hr at RT. The excess liquid was removed and 2.3 ml of liquid phage lysate was added. After an o/n incubation at RT, the agar surface was rinsed with 5 ml of SM buffer (Supplementary Table S1) and incubated for 10 min. The SM buffer was then recovered and chloroform added to 1% (v/v) final concentration. After 5 min incubation, bacteria and other debris were pelleted by centrifugation at 3000 × g at 4°C for 15 min. The supernatant was filtered through a 0.22 μm filter and stored at 4°C. This method is modified from the one used for phage propagation in the Gabrichevsky Institute of Epidemiology and Microbiology (Moscow, Russia).

### Titration by drop-test

Titration of the phage lysates was performed by drop tests using the double layer method. Ten-fold serial dilutions of the phage lysates were prepared in LB. The double layer plates were then prepared with 45 μl of overnight bacterial host culture mixed with 3 ml of 50°C-adjusted soft-agar. The mixture was immediately poured on the agar plate. After solidification of the agar, 10 μl drops of the serially diluted phage lysates were pipetted onto the plates. A negative control with just LB was always pipetted as well. After overnight growth at RT the titre in plaque forming units (PFU) per ml was ready to be estimated (number of plaques × dilution factor × 100). A modification of this method with whole plates was done to calculate more accurate titres. Briefly, the double layer plates were prepared with 45 μl of overnight bacterial host culture and 50 μl of the desired dilution of phage lysate mixed with 3 ml of 50°C-adjusted soft-agar.

### Ultracentrifugation

Ultracentrifugation was performed using a step-gradient of 1 ml TM-40% sucrose on the bottom and 1.5 ml TM-5% sucrose on top (Supplementary Table S1) prepared to the tubes of Beckman Coulter Optima L-80 XP ultracentrifuge with the rotor SW55 Ti. The tubes were centrifuged at 35 000 rpm at 4°C for 3 h. The phage pellet was resuspended into 500 μl of LB.

For phage samples used for TEM, phage lysate (∼5 ml) was applied to the top of a stepped caesium chloride gradient in a SW28 Ultra-Clear tube (Beckman), comprising of solutions of densities 1.23, 1.31, 1.40 and 1.53 g/ml in 20 mM Tris–HCl pH 7.5, 10 mM MgSO4. Gradients were centrifuged at 28 000 rpm at 4°C for 3 h. The band containing mature phage particles formed at the 1.40–1.53 g/ml interface, which was extracted by needle side-puncture. Samples were dialysed against 20 mM Tris–HCl pH 7.5, 150 mM NaCl, 10 mM MgSO_4_.

### Transmission electron microscopy

For negative staining, carbon-formvar coated copper grids (Agar Scientific Ltd.) were plasma cleaned in a PELCO easiGlow for 60 s at 0.38 mbar (air) and 20 mA. Phage solution (5 μl) was applied to a grid for 1 min then removed with filter paper. The grid was washed with 5 μl deionised water and stained with 5 μl of 2% (w/v) uranyl acetate (Agar Scientific Ltd.). Grids were imaged using an Tecnai 12 G2 BioTWIN microscope with tungsten filament operating at 120 kV, with a Ceta 16M camera (Thermo Fisher Scientific, USA). For cryo-electron microscopy, lacey carbon grids supporting an ultrathin carbon layer on 400-mesh copper (Ted Pella) were glow discharged as above. Phage samples were vitrified in a Vitrobot mark IV. Grids were imaged on a 300 kV Titan Krios (Thermo Fisher Scientific, USA) and images recorded on a Falcon3EC detector (Thermo Fisher Scientific, USA) in linear mode. The nominal magnification was 59 000×, the calibrated pixel size was 1.39 Å/pixel, the nominal defocus range was 0.5–1.7 μm, and the total dose was 50.5 e/Å^2^. Images were processed in RELION3.1, using RELION’s movie motion correction (14). CTFs were estimated using CTFFIND4 (15) using dose-weighting. After manual picking and 2D-classification, particles were manually selected to ensure only DNA-filled particles were included, resulting in a set of 2900 particles. These were extracted, resampling to a final pixel size of 1.507Å/pix with a resulting 1000 pixel box size. Particle orientations were refined imposing icosahedral symmetry. After initial refinement, the CTF estimates were further refined on a per-particle basis using magnification anisotropy estimation. After refinement, the final masked resolution (FSC 0.143) was 5.62 Å. The map was sharpened in postprocessing with an automatically estimated *B*-factor of –382 Å^2^. Radial averaging was performed in EMAN2 (16), and density profiles were calculated in ImageJ (17). Figures and model fits were prepared using UCSF Chimera (18) and ChimeraX (19).

### Modelling of the YerA41 DNAP candidate proteins

Fold recognition searches of the YerA41 DNAP01, DNAP02 and DNAP03 were carried out using Phyre2 (20), a web-based fold recognition server, suggested at the strong conservation of the secondary structure architecture of the C-terminal part of the DNAP01 protein (amino acid residues 946–1215, i.e. DNAP01-Ct), DNAP02 and DNAP03 among the nucleic acid transferase and nucleic acid polymerase structures. Though having very low sequence identity, DNAP01-Ct, DNAP02 and DNAP03 were noticed to be structurally well conserved among different transferase/polymerase-like folds. The DNAP01-Ct structure was modelled based on the previously solved DNA polymerase structure (PDB: 1NJZ, chain A) (21) with which it was found to have only 26% sequence identity. The DNAP03 structure was modelled using a human DNA polymerase beta structure (PDB: 8ICZ, chain A, 4N-326S) (22) with which it was found to have only 26% sequence identity. The models were built using PRIME module of Schrödinger suite version 2018-4, (Schrödinger LLC) (23,24) via the Maestro interface. The final model thus generated was manually checked specially in the non-similar residues with its corresponding templates. The model structure was also verified looking into its Ramachandran diagram.

### Phage genomic material isolation by phenol–chloroform extraction

YerA41 genomic material was isolated from 400 μl of ultracentifuge-concentrated lysate. First, 1.3 μl of DNase I (1 U/ml) and 0.5 μl RNase A (10 mg/ml) were added. After an incubation at 37°C for 30 min, 16 μl of 0.5M EDTA, 1.2 μl of Proteinase K (20 mg/ml) and 20 μl of 10% sodium dodecyl sulphate (SDS) were added. After a 60-min incubation at 56°C, the tube was cooled down to RT, and 1 volume (VOL) of phenol (450 μl) was added. The tube was mixed for 15 min followed by centrifugation at full speed for 5 min. The aqueous upper phase was transferred into a new tube. The same procedure was repeated with 1 VOL phenol–chloroform (1:1) and then, finally, with 1 VOL chloroform. The nucleic acids were then precipitated using 0.1 VOL of 3 M NaOAc (pH 7.0) and 2 VOL absolute EtOH. The precipitated nucleic acid thread was transferred into a new tube with 1ml of 70% (v/v) EtOH. The tube was then centrifuged at RT for 15 min at full speed, the supernatant removed, and the pellet was allowed to air-dry. Finally, the pellet was dissolved in 100 μl Tris buffer on ice and incubated at –20°C overnight. After that, it was stored at –70°C due to the instability of YerA41 nucleic acids at other temperatures. From every 400 μl of lysate, 200 μl of genomic material with a concentration of around 50 ng/μl could be isolated.

### Nucleoside analysis

Nucleoside Digestion Mix (New England Biolabs, Ipswich, MA, cat#M0649S) was used to digest YerA41 genomic material into nucleosides. Briefly, 50 μl reactions were prepared with 2 μl of the enzyme mix, 5 μl of the 10× nucleoside digestion mix buffer and 43 μl of YerA41 genomic material. Samples were incubated overnight at 37°C and stored at 4°C. The LC-MS chromatograms of YerA41 genomic samples digested with the Nucleoside Digestion Mix were recorded with a Agilent Technologies 1260 Infinity Binary LC and Agilent 6100 Series Quadrupole LC/MS Systems with Phenomemex 150 × 4.6 SynergiTM 4 μm Fusion-RP 80 Å analytical column at the University of Turku, Finland. The nucleoside samples were eluted for 2.5 min with 1% MeCN and 99% formic acid (0.1%) buffer in mQ water, and then increasing MeCN volume linearly to 10% at 25 min (25). Tandem mass spectroscopy (LC–MS/MS) was recorded with Thermo Scientific Q Exactive Hybrid Quadrupole-Orbitrap Mass Spectrometer using HPLC conditions mentioned above.

### Reverse transcription

Total RNA from YerA41-infected bacteria had been previously extracted using the Promega SV total RNA isolation system (8). Reverse transcription to generate cDNA was performed using the Moloney Murine Leukaemia Virus Reverse Transcriptase (M-MLV RT; Promega, USA, cat#M170A). The template was mixed with random hexamers, it was heated to for 5 min and then cooled immediately on ice for at least 3 min. Afterwards, as requested by the protocol provided by the manufacturer, additional reagents were added to the reaction mix: 1 × M-MLV RT Reaction Buffer, 0.5 mM dNTPs, 25 U Recombinant RNasin^®^ Ribonuclease Inhibitor and 200 U M-MLV RT. Then it was incubated at 37°C for 90 min.

### PCR

DreamTaq (Thermo Fischer Scientific, USA, cat#EP0701) was always used as thermostable DNA polymerase, unless stated otherwise. A final reaction volume of 50 μl was always used. Into that, the buffer provided by the manufacturer was used (5 μl 10×); dNTPs were added to a final concentration of 200 μM each; primers (Supplementary Table S2) were added to a final concentration of 0.5 μM concentration each (2.5 μl of 10 pmol/μl); 10–50 ng of DNA template were added (1 μl), and the DNA polymerase was added to have the final quantity requested by the manufacturer's protocol (0.25 μl). The cycles were performed as recommended by the manufacturer's protocol. Briefly, initial denaturation at 95°C for 2 min and 35 cycles with denaturation at 95°C for 30 s, annealing at 55°C for 30 s and extension at 72°C for 1 min; then, a final extension at 72°C for 10 min was performed. PCR results were analysed using 1% agarose gel (Seakem LE in Tris-Acetate-Ethylenediaminetetraacetic (TAE) buffer, Supplementary Table S1).

### Electrocompetent cells

Electrocompetent *E. coli* strain BL21 (DE3) cells were prepared as described (26). Briefly, the bacteria were grown in Super Optimal Broth (SOB) media. After overnight incubation, they were diluted 1:250 and grown until the OD_600_ was 0.6–0.8 with vigorous agitation (220 rpm). The cells were then collected by centrifugation (1500 × g at 4°C) and washed twice with original culture volume of 10% glycerol. After that, they were resuspended in 1–2 ml of 10% glycerol, aliquoted at 100 μl into Eppendorf tubes, flash frozen in liquid nitrogen and stored at –75°C. For each group of electrocompetent cells made, a test was performed in order to ensure that there was no contamination and competence status was checked with intact plasmid (pUC19). The competent cells used in this study produced 4.68 × 10^10^ CFU/mg of pUC19.

### Electroporation

Electroporations were carried out with 2 mm cuvettes unless stated otherwise. Competent cells were melted on ice and the desired ligation reaction or plasmid was added (1–3 μl) into 45 μl of cells. The mixture was transferred into cold cuvettes and the cuvette inserted into the electroporation apparatus. A pulse was then given (200 Ω, 25 μF, 2.5 kV). Afterwards, 1 ml of prewarmed SOC (Supplementary Table S1) was added and cells were transferred into a 1.5 ml Eppendorf tube and incubated for 45 min at 37°C with gentle agitation. After incubation, 100 μl undiluted and 100 μl of 1:100-diluted samples were spread to appropriate antibiotic plates that were incubated overnight at 37°C. If there was growth, between four and eight colonies from each plate were then streaked on new antibiotic plates (Supplementary Table S1).

### Determination of target protein solubility

Soluble proteins should be purified under native conditions and insoluble proteins or inclusion bodies should be purified under denaturating conditions (The QIAexpressionist June 2003. A handbook for high-level expression and purification of 6xHis-tagged proteins 5th ed. Qiagen). In order to know if the protein is soluble or insoluble and purify it accordingly we performed the following assay. Ten ml of LB containing the appropriate antibiotics were inoculated with the desired producer of the target protein and grown overnight at 37°C. Then, 50 ml of prewarmed LB were inoculated with 2.5 ml of the overnight culture and the bacteria were grown until the OD_600_ was 0.5–0.7. Then a 1-ml sample was withdrawn and cells were pelleted and resuspended in SDS-PAGE sample buffer (Supplementary Table S1). This represented the non-induced raw control lysate. In the rest of the culture, expression was induced adding IPTG to a final concentration of 0.5 mM. The cultures were then grown overnight at 15°C with vigorous agitation (270 rpm), another 1 ml sample was withdrawn, pelleted and resuspended in SDS-PAGE sample buffer. This was the induced raw control lysate. The bacteria from remaining culture were harvested by centrifugation at 4000 × g for 20 min. Proteins were extracted by resuspending the pellet in 5 ml lysis buffer (Supplementary Table S1), and the sample was then frozen and thawed in cold water. Sonication was then performed with Branson sonifier (Branson Ultrasonics, Danbury, USA) using a pulse mode of 30% and a loading level of 2 for 30 s 10 times with 30 s pauses. Lysate was then centrifuged at 10 000 × g at 4°C for 25 min. Supernatant was decanted and saved on ice (soluble protein). The pellet was resuspended in 5 ml lysis buffer (insoluble protein). All the obtained samples were analysed using SDS-PAGE analysis.

### Purification under native conditions

Purification of a soluble protein was carried out under native conditions. Fifty ml of SOB with the appropriate antibiotics was inoculated with the producer strain. After overnight growth, the culture was diluted 1:100 into 100 ml of medium and incubated at 37°C until OD_600_ of 0.6 was reached, then IPTG was added to 0.5 mM final concentration. The induced culture was incubated overnight at 15°C and then a 1 ml sample was withdrawn, bacteria were pelleted and frozen. The remaining bacteria were harvested by centrifuging at 4000 × g for 20 min. The pellet was resuspended in a 5-ml lysis buffer containing 1 tablet of protease inhibitor (Pierce™ Protease Inhibitor mini Tablets, EDTA-free, Thermo Fisher Scientific, USA, cat#A32959) and 1 mg/ml of lysozyme (these amounts were used for every 100 ml of culture). Cells were then sonicated as stated above. After centrifuging for 25 min at 10 000 × g at 4°C, the supernatant was introduced into a new Falcon tube, a 10 μl sample was taken for future SDS-PAGE analysis. The supernatant was then mixed with 2.5-ml of Ni-NTA agarose (Qiagen, The Netherlands, cat#30250) for 60 min at 4°C. Note that the Ni-NTA agarose beads were washed twice with washing buffer before use. After the incubation, the non-bound fraction was separated from the beads; another 10 μl sample was taken for further analysis. The agarose was then washed six times with one volume of washing buffer (Supplementary Table S1), from the first and last wash a 10 μl sample was taken. Elution was carried out in four steps where 1 ml elution buffer (Supplementary Table S1) was used. In order to separate the liquid from the beads in every step of the process, the agarose was spun for 10 s at 1000 × g at 4°C. All the samples, together with the 10 μl samples from the eluted fractions, were analysed by SDS-PAGE. After purification, the solution was changed to lysis buffer without imidazole (50 mM NaH_2_PO_4_; 300 mM NaCl) using a PES membrane (Vivaspin 4/20 10 000 MWCO, Sartorious) to eliminate imidazole without affecting the protein.

### Anion exchange chromatography

Anion exchange chromatography was performed using Äkta Pure Chromatograph System. Concentrated protein was diluted 1:5 in 50 mM NaH_2_PO_4_ buffer to adjust the salt concentration to that of buffer A. A 5 ml loading loop was used to introduce the sample into the 1 ml column YMC BioPro q75. The column was then washed ten times with buffer A (50 mM NaH_2_PO_4_, 60 mM NaCl, pH 8.0), after which the salt concentration was raised linearly during 20 column volumes until 70% of buffer B was achieved (50 mM NaH_2_PO_4_, 1 M NaCl, pH 8.0). Elution of the protein took place at a concentration of 30% of buffer B that corresponds to a 350 mM NaCl concentration. The eluted fractions that contained pure protein were pooled and concentrated as stated above.

### Protein analysis by mass spectrometry

Mass spectrometry analyses of purified protein samples were performed at Proteomics Unit, Institute of Biotechnology, University of Helsinki, as described earlier (27,28). Two kinds of DNAP01 samples were sent for mass spectrometry, a sample that had only been purified using the Ni-NTA agarose, and another that had been further purified with AIEX.

### BCA protein assay

The PierceTM BCA protein assay kit (Thermo Fisher Scientific, USA, cat#23225) was used for the determination of the protein concentration using a spectrophotometer and Bovine Serum Albumin standards with different concentrations. The working reagent was prepared by mixing 50 parts of reagent A with 1 part of reagent B. 25 μl of each sample were pipetted into microtitre plate wells containing 200 μl of working reagent, and the plate was incubated at 37°C for 2 h. Then it was cooled down at RT for 10 min and the absorbance at 562 nm was measured. Concentration was calculated using the Bovine Serum Albumin standard to create a standard curve, through linear regression based on that curve, the protein concentration could be calculated for any given absorbance.

### Functional assay using YerA41 genome as template

The isolated phage YerA41 genomic material was treated in a 15 μl reaction volume following the protocol of reverse transcription, but replacing the reverse transcriptase with the YerA41 DNAP as the enzyme. Primers, random hexamers or water were added depending on the tested conditions. The incubation times ranged between 1 and 90 min. The negative control reactions were set up either without phage DNA, without DNAP01, or without random primers, and in the positive control, the YerA41 DNA was replaced by the YerA41 cDNA generated for RNA-seq (Supplementary Table S3). After these pre-incubations, 1 μl samples were used as templates for different conventional PCRs (Supplementary Figure S1).

DreamTaq was used as DNA polymerase and the conventional PCR protocol was followed (see above). Five different YerA41 gene specific primer pairs were used (Supplementary Table S2): gp8 (phage tail protein (8); product size: 648 bp); gp37 (major capsid protein(8); product size: 521 bp), and dnap01 (product size: 621 bp), dnap02 (product size: 419 bp) and dnap03 (product size: 494 bp). Primers were designed using Primer3Plus, with a product length of 400–700 bp. The primers were chosen so that each one of them would have a different product length and they could be easily identified in an agarose gel.

### Completion of the genomic DNA sequence of YerA41

Eighteen primers (Supplementary Table S2) were designed pointing outwards from each of the nine RNA-seq assembled sequence scaffolds of YerA41(8). These were tested in different combinations using as template the genomic material of YerA41 preincubated with DNAP01 in the presence of dNTPs. Analogous to the functional assay (see above), 1 μl of the pretreated genomic material was then PCR-amplified using Phusion DNA polymerase (Thermo Fisher Scientific, USA, cat#F-530) in 1 × GC buffer according to manufacturer's recommendation. The PCR products were purified using the NucleoSpin Gel and PCR Clean-up kit (Macherey-Nagel, GmbH Düren, Germany), and subjected to Sanger sequencing using the PCR-amplification primers individually (29), at the Institute for Molecular Medicine Finland Technology Centre Sequencing Unit. The obtained sequence reads and the sequences of the original scaffolds were assembled using the Staden package (30). To fill in remaining gaps, two additional primers (A2b and A3b) were designed and used in combination with the previous primers for PCR and Sanger sequencing to complete the genome. The genome was annotated, and the promoters and terminators were predicted using the BPROM and FindTerm tools (31).

### Commercial DNA polymerase functional assay

EvaEZ Fluorometric Polymerase Activity Assay Kit (Biotium, USA, cat#29051) was used for this functional assay. The protocol followed was the one recommended by the manufacturer for the measurement of fluorescence change by polymerization. Two positive controls with dilutions of T4 DNA polymerase (Thermo Fischer Scientific, USA, cat#EP0061) and a negative control that contained only water, were also included. The fluorescence was measured with a qPCR Bio-rad machine, taking a measurement every 30 sec for an hour. Fluorescence was measured in Channel 1 (the channel for FAM).

The EvaEZ Fluorometric Polymerase Activity Assay Kit was used to determine the specific activity of the DNAP01 polymerase. The protocol followed was the one recommended for the determination of activity of an unknown polymerase sample based on a standard. A standard curve was determined using serial dilutions of T4 DNA polymerase. Different dilutions were prepared for the protein of interest. A duplicate was prepared for each sample and the average was calculated. The fluorescence was measured with a qPCR Bio-rad machine, taking a measurement every 30 sec for an hour. Fluorescence was measured in Channel 1 (the channel for FAM).

## RESULTS

### YerA41 genome nucleotide composition

YerA41 genomic material was isolated using the phenol–chloroform extraction method from ultracentrifuged YerA41 lysates with titres between 1 × 10^15^ and 1 × 10^19^ PFU/ml to elucidate whether nucleotide modifications were behind the observed restriction enzyme resistance of the YerA41 DNA (8). The isolated genomic material was analysed in 1% agarose gel analysis (Supplementary Report Figure I A). While the genomic material gave a clear distinct band in the agarose gel and its UV absorbance 260/280 ratio was near 1.80, it migrated aberrantly in the gel, much slower than the anticipated size of the DNA genome, ca. 145 kb (8) (Supplementary Report Figure I B). Of note, both the phage particles and the isolated genomic material were unstable and were best stored frozen.

The YerA41 genomic material was digested into nucleosides using the Nucleotide digestion mix, and analyzed by chromatography and mass spectrometry to elucidate the nature of the nucleosides (Supplementary Report Figure I C). Interestingly, apart from the canonical deoxyribonucleosides, deoxyadenosine (dA), deoxyguanosine (dG), deoxycytosine (dC) and thymidine (T) present in DNA, ribonucleosides adenosine (A), guanosine (G), cytosine (C) and uridine (U), usually present in RNA molecules, were also found (Supplementary Report Figure I C). While these results indicated that both DNA and RNA are unconventionally present in the genomic material isolated from YerA41 bacteriophage, further investigations, reported in details in the Supplementary Data (Supplementary Report Figures II, III, IV and V, and Supplementary Report Tables I, II and III), revealed that phage lysates of YerA41 were exceptionally heavily contaminated with ribosomes of the host bacteria. The RNase A treatment of the phage lysate prior to DNA isolation was not able to degrade the rRNA embedded in the ribosome structure (Supplementary Report Figure IV) and this was detected as ribonucleosides in the samples. It should be noted that such extensive ribosome contamination of phage stocks is exceptional and not seen with other phages.

The relative proportions of different nucleosides in the YerA41 genomic material (Supplementary Report Figure I C) were estimated based on HPLC peak areas compared to those of the standard nucleoside mixtures (50 μM) (Table 1). While the relative proportions of ribonucleosides reflect the proportions of dNTPs present in the transcribed template DNA, the proportions of deoxyribonucleosides should follow the base-pairing rules such that dA and T should be present in equal proportions as should also dC and dG. The results for dC and dG are very close to each other as expected, but a major discrepancy is present in the relative amounts of dA and T, with T hugely underrepresented (Table 1). A possible explanation for the discrepancy could be that a proportion of T could be modified and the modified nucleotide could co-migrate with the other nucleosides in the sample, thus causing distortion.

**Table 1.** Relative amounts of DNA and RNA-derived nucleosides in YerA41 Genomic samples analyzed from HPLC data

	DNA %					RNA %				
Sample	dA	dG	dC	T	Total	A	C	G	U	Total
1	38.6	21.0	24.8	15.6	100.0	18.5	14.5	55.5	11.6	100.1
2	40.1	19.4	25.1	15.3	99.9	24.9	14.7	44.3	16.1	100.0

The HPLC analysis was repeated for sample 2 (bottom graph, Supplementary Report Figure I C) and careful analysis of the results revealed additional peaks. MS-ESI (Orbitrap, positive mode) analysis of the broad low intensity peak at 16.5 min revealed the presence of a 1102 *m*/*z* molecule. In addition, a 918 *m*/*z* molecule co-migrated with dG, and a 1286 *m*/*z* molecule with dA. The 918 and 1286 *m*/*z* molecules both differ from the 1102 *m*/*z* molecule by a fragment of 184 *m*/*z*. Interestingly, MS/MS-analysis of each molecule (918, 1102 and 1286 *m*/*z*) showed a common cleaved fragment of 346 m/z (plus the observed corresponding counterpart fragments: 572, 756 and 940 m/z) (Supplementary Figures S2 – S7). This fragment of 346 m/z most likely corresponds to a thymidine molecule containing a spacer moiety (T plus spacer = 242 + 104 = 346). However, the observed masses do not match to typical hexose-, hexosamine- or *N*-acetylhexosamine derived fragmentations, which would contribute mass differences of 162, 161 or 203 *m*/*z*, respectively. We interpret the obtained results that the hypothetical substitution would carry at least two 184 *m*/*z* distal residues. Assuming the 1286 *m*/*z* molecule corresponds to a hypermodified thymidine, an additional 1044 Da per thymidine is introduced. Based on the presence of several genes encoding proteins with glycosyltransferase motifs in the genome of YerA41 (8), the structure of this modification is likely a novel type of oligosaccharide. Further studies are underway to provide complete characterization of these hypermodifications.

### Transmission electron microscopy of YerA41 phage particles

**Figure 1.**
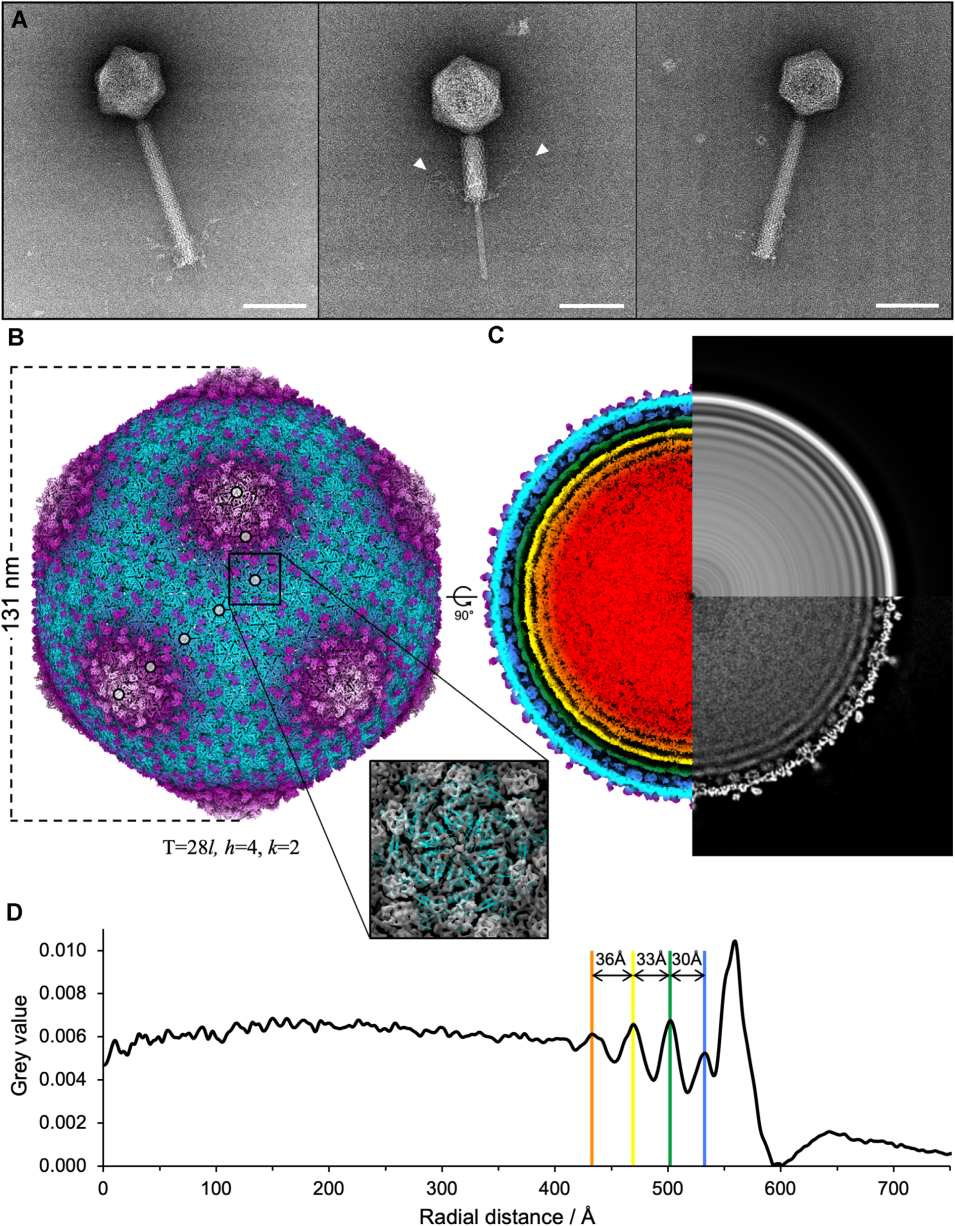
Electron microscopy analysis of YerA41 virions. **Panel A**. Negatively stained electron micrographs, left and central panels are YerA41-B, and right panel is YerA41-S. White triangles indicate tail fibers. Scale bar 100 nm. **Panel B**. Cryo-electron microscopy icosahedral reconstruction of YerA41-B capsid, coloured radially. Circles indicate *h* = 4 and *k* = 2 values that describe the T = 28 quasi-symmetry. Square panel shows fitting of HK97 major capsid protein (PDB 1OHG), in blue, into the YerA41-B map in grey. **Panel C**. Left, cross-sectional view along the z-axis (rotated 90° with respect to panel A), coloured radially. Capsid proteins are in purple and cyan, internal capsid proteins in blue, and genomic layers are in green, yellow, orange and red. Bottom right, central slice through the reconstruction in greyscale. Top right, rotationally averaged central slice in greyscale. **Panel D**. Density profile of rotationally averaged central slice, corresponding to top right section of panel C, plotted against radial distance from centre of the capsid. Colours correspond to radial layers in panel C.

The YerA41 virions exhibited a typical myovirus morphotype, with large icosahedral heads, 131 nm diameter, and tails ∼241 nm long that occasionally appeared in a contracted conformation (Figure 1A). Tail fibers protruded from the tail baseplate (Figure 1A, white triangles central panel). These dimensions are very close to those of Ackermann et al, with heads of about 110 nm in diameter, 10 nm × 8 nm necks, 250 nm × 20 nm non-contracted sheaths, and 70 nm long tail fibers (32). A minor population of tailed virions with smaller ∼103 nm heads was also present (Figure 1A, right panel), hereafter called YerA41-S to differentiate it from the bigger YerA41-B. From their appearance, YerA41-S were also apparently filled with nucleic acid.

The diameter of YerA41-B places it within the range for jumbophage capsids (33), despite YerA41 possessing a genome of <200 kb. To understand the discrepancy between the high storage capacity and low genome length, cryo-electron microscopy analysis of the YerA41-B particles was carried out.

YerA41 exhibits a T-number of 28, and *laevo* handedness (Figure 1B). Fitting of the HK97 major capsid protein fold confirmed the handedness and the family of YerA41 major capsid protein fold (Figure 1B). On the surface of the capsid, additional proteins, potentially auxiliary proteins, were present at the local and icosahedral 2-fold positions, and weaker density a fiber protruding from the centers of hexons was also present (Figure 1C, bottom right). Inside the capsid, internal capsid proteins protruded from the inner surface of the wall (Figure 1C, blue layer). The presence of internal proteins meant that the first layer of genomic material was shifted further towards the capsid core than it would be if only the major capsid protein were present. Three layers of genomic material could clearly be defined, with a less well defined fourth layer (Figure 1C, green/yellow/orange layers). Radial averaging of the central slice on the 5-fold symmetry axis allowed estimation of the spacing between protein and genomic layers (Figure 1C, top right, and Figure 1D). These were: 30 Å between the internal capsid proteins and the first genomic layer (blue and green layers), 33 Å between the first and second genomic layers (green and yellow layers) and 36 Å between the second and third genomic layers (yellow and orange layers), together demonstrating a relatively low density of packing. The internal volume of the YerA41-B capsid, inside of the internal capsid proteins, was ∼620 × 103 nm^3^. The calculated genomic density was therefore ∼0.23 bp/nm^3^, assuming one copy of a 145 577 bp genome per head.

### Identification of the DNAP encoding genes

Previously we were able to determine partial genomic sequence of phage YerA41 by sequencing RNA isolated from YerA41-infected *Y. ruckeri* bacteria, altogether ca. 145 kb in nine sequence scaffolds (8). Among the 201 predicted gene products three showed remote similarity to DNA polymerase like enzymes and they were preliminarily named DNAP01, DNAP02 and DNAP03 (Supplementary Table S4). The amino acid sequence similarity search using BLASTP (34,35), identified a multitude of homologs that aligned with the C-terminal third of DNAP01 sequence (DNAP01-Ct). The conserved DNA polymerase PolA motifs A, B and C could be identified from the sequences of several of the most closely related proteins (Supplementary Figures S8 and S9) further confirming the possibility that DNAP01 is a functional DNA polymerase of YerA41. The profile-profile comparison methods, HHblits and HHpred (36), indicated homology of DNAP01 with other DNA polymerases. The HHblits displayed with *E*-value of 3.3e–20 and 864 aligned columns, similarity with DNA polymerase A (POLAc) domain containing protein of *Campylobacter jejuni* (UniRef100_A0A430VC34). The HHPred search showed similarity of the C-terminal end of DNAP01 with different DNA polymerases. On the other hand, the N-terminal residues 227–577 showed similarity to the amino acid residues 1–253 of T7 DNA polymerase in complex with an *N*-2-acetylaminofluorene-adducted DNA (1X9M) with 93% probability and e-value of 0.85. While the closest BLASTp hits of DNAP02 were putative DNA polymerases (Supplementary Figure S10), HHPred analysis revealed that it resembled most closely 5'-3' exonucleases, therefore it was not studied further. The best BLASTp hits of DNAP03 were all PolX type DNA polymerases (Supplementary Figure S11) that typically function as gap-filling polymerases (37), therefore DNAP03 might not be a truly processive DNA polymerase. DNAP01-Ct and DNAP03 were structurally modelled (Supplementary Figures S8 and S11). As the structural modelling of DNAP01 predicted that the DNA polymerase activity would reside in its C-terminal domain (DNAP01-Ct, residues 946–1306 of the full-length, Supplementary Figure S8), it was also selected for expression cloning. Regarding the modelling of DNAP03, there is no sequence similarity in the amino acid stretch of 180–220 (DNAP03 numbering) between DNAP03 and the human DNA polymerase beta structure 8ICZ that was used as a template to model DNAP03. The modeling of this region (180–220 of DNAP03) was carried out according to the 8ICZ structure based on the similarity of the predicted secondary structure of DNAP03 and the solved 8ICZ structure.

The genes encoding for the polymerase candidates were amplified by PCR using the YerA41 mRNA-derived cDNA as template and cloned into an expression vector pET28a. While the gene encoding DNAP03 did not yield any protein, and the expressed DNAP02 showed no DNA polymerase activity, the DNAP01-Ct was expressed and its DNA polymerase activity could be proved (Supplementary Figure S12). Due to the poor solubility and low stability of DNAP01-Ct, a pET28a codon-optimised vector expressing a His-tagged full-length DNAP01 was custom synthesised and further characterized in this study.

### Isolation and purification of DNAP01

The codon optimised gene encoding DNAP01, cloned in plasmid pET28a-DNAP01, expressed the gene in *E. coli* strain BL21 (DE3) and produced in good quantities a soluble product (Supplementary Figure S13). The DNAP01 protein was purified under native conditions using the Ni-NTA agarose with a yield 980 μg/ml. Ultrafiltrated proteins were subjected to anion exchange chromatography (AIEX) to further purify the protein. The obtained peak fractions were pooled and concentrated as above yielding a highly purified protein (Supplementary Figure S13). The increased purity was further confirmed by proteome analysis of the pre-AIEX and AIEX-purified samples. The LC/MS-MS analysis identified 283 *E. coli* proteins from the pre-AIEX sample compared to only 32 hits for the AIEX-purified protein, over the PSM threshold of 4 (Supplementary Table S5). Remaining in the AIEX-purified sample a distinct > 70 kDa band was visible in SDS-PAGE (Supplementary Figure S13). In the LC/MS-MS results three proteins had a score above 200, of which the 74.2 kDa ArnA protein had the highest score (Supplementary Table S5), consistent with the size of the band present in the SDS-PAGE. ArnA (https://www.uniprot.org/uniprot/A0A140N587) is required for lipopolysaccharide-mediated polymyxin B resistance. The other two high-score proteins were smaller, <70 kDa in size. We cannot explain why ArnA would copurify with DNAP01. It either interacts directly with DNAP01 or with the AIEX column and elutes under the same conditions as DNAP01 does.

### DNAP01 shows DNA polymerase activity with YerA41 genomic material even without external primers

**Figure 2.**
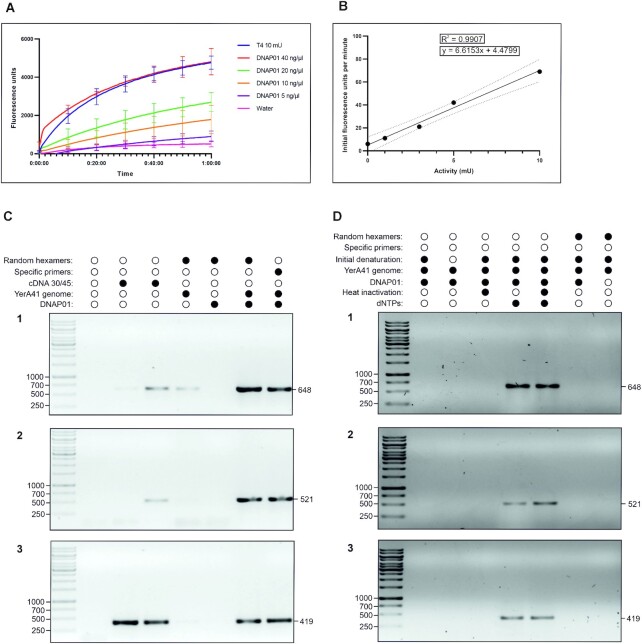
DNAP01 shows DNA polymerase activity also with YerA41 genome even without adding external primers. **Panel A**. DNA polymerase activity of DNAP01 compared with T4 DNA polymerase, measured with a commercial assay for 60 min. Each line represents the mean of three replicates. Negative control is represented by water. **Panel B**. Standard curve calculated with different concentrations of T4 DNA polymerase with known activity. Dashed lines represent the 95% confidence interval of the trend line created with the regression model shown in the same graphic. **Panels C and D**. 1% agarose gels of PCR results of the functional assay performed with YerA41 genomic material. 1. gp8 primers; 2. gp37 primers 3. dnap02 primers. At the top, the reagents added or any special condition applied during the treatment with DNAP01 is shown. All those samples were then used as template for PCR with Dream-Taq polymerase, as described in the methods section, and shown here are the products of those PCRs. The sizes of the bands are given in bp.

The DNA polymerase activity of DNAP01 was first verified using the EvaEZ Fluorometric Polymerase Activity Assay Kit where DNAP01 showed a clear dose-dependent polymerase activity (Figure 2A). Using a T4 DNA polymerase standard curve (Figure 2B) the polymerase activity of 7.73 (±1.17) mU/μg was estimated for DNAP01 where an enzyme unit catalyzes the incorporation of 10 nmol of deoxyribonucleotides into a polynucleotide fraction in 30 min at 37°C (38).

The presence of DNAP01 in the phage particles (8) implicates that it is delivered along with the genomic material from the phage particles upon infection into the host bacteria. DNAP01 should inherently possess the ability to use the YerA41 DNA as template. To confirm this, we set up an in-house assay where we used YerA41 genomic material as template. We first incubated the genomic material with different combinations of DNAP01, dNTPs, random hexamer primers, or specific YerA41 DNA primers with and without heat treatment. After the incubation, conventional PCRs with YerA41-specific primer pairs were carried out, and the products analysed in an agarose gel (Figure 2C). These results demonstrated that DNAP01 was indeed able to replicate the YerA41 DNA thereby creating a non-modified DNA-product that a thermostable commercial DNA polymerase was able to use as template in a subsequent PCR. Furthermore, the DNAP01 polymerase did not require any added specific or random hexamer primers for its activity with the YerA41 genomic material. This was demonstrated by samples that contained only DNAP01 mixed with YerA41 genomic material, the appropriate buffer and dNTPs, that gave a positive band when used in PCR with Dream-Taq (Figure 2D). This shows that DNAP01 DNA polymerase activity was fully operational with YerA41 genomic material without the need of any external primers.

### RNase A treatment does not influence the activity of DNAP01

**Figure 3.**
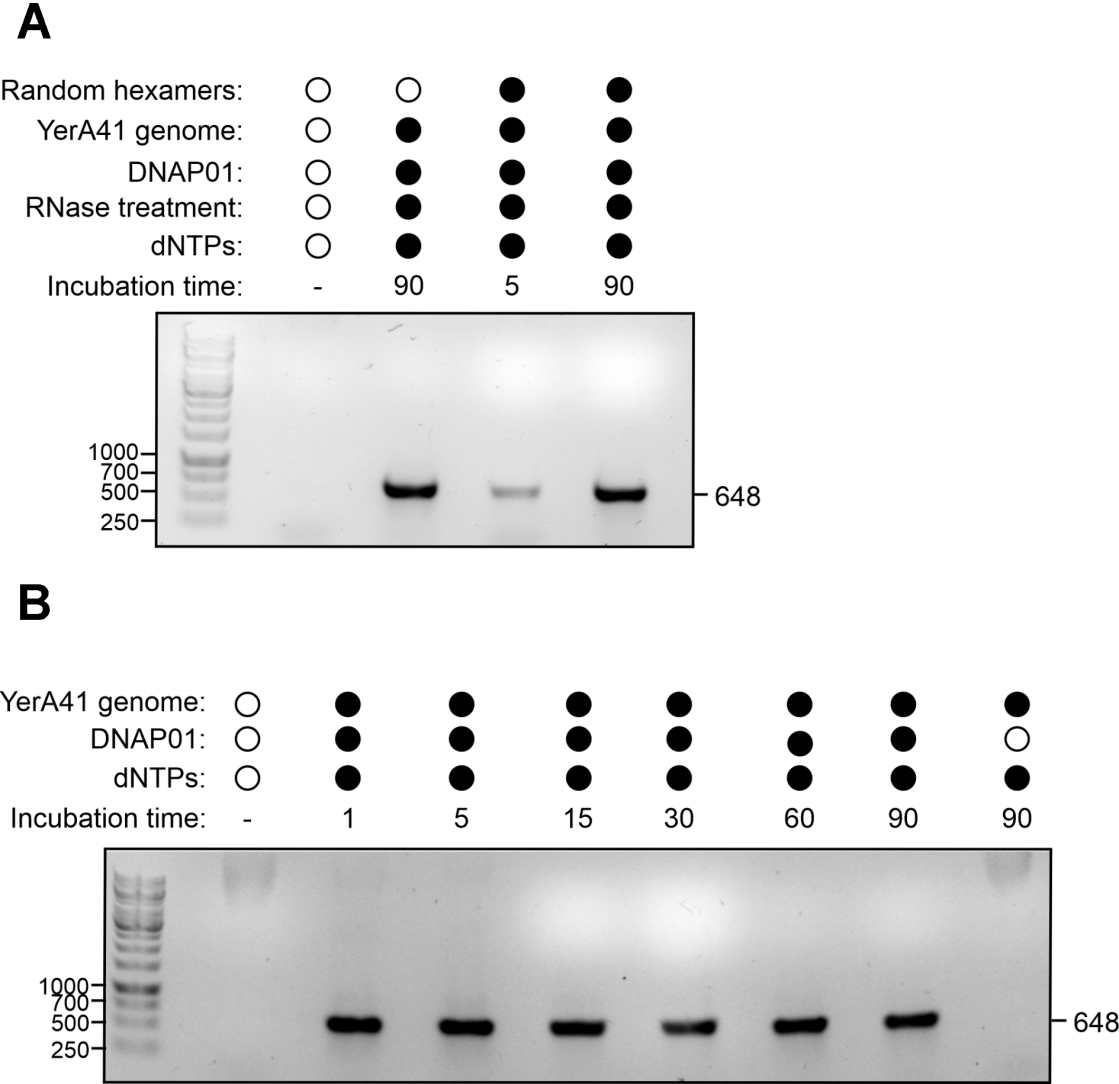
RNase A treatment does not affect activity of DNAP01, which produces enough extension even in 1 min. **Panel A**. Influence of RNase A treatment on DNAP01 activity. **Panel B**. Influence of incubation time on DNAP01 activity. In both panels, the obtained PCR products were analysed in 1% agarose gel electrophoresis. Above the gel images are indicated the added reagents and any special conditions applied to the DNAP01 incubation. Aliquots of the samples were used as templates for PCR with Dream-taq using gp8 primers, as described in the methods section. The sizes of the bands are given in bp, and the incubation times, in min.

It was possible that the RNA fragments complexed with the YerA41 DNA could act as primers for DNAP01. As RNase A treatment changed the migration of the YerA41 genomic material in the agarose gel (Supplementary Figure S2a), we tested whether the RNase A treatment would prevent DNAP01 activity. That was not the case as DNAP01 was still able to replicate the DNA without primers (Figure 3A). DNAP01 demonstrated apparent high progressivity in experiments where it was incubated different times with the YerA41 genomic material as a mere one-minute incubation was sufficient for DNAP01 to produce a template for the detection PCR (Figure 3B).

### YerA41 complete genome was determined using DNAP01 treated samples

Previously, sequencing of the YerA41 genome could only be performed by RNA sequencing of the RNA isolated from host bacteria infected with the phage (8). The DNAP01 activity opened a new possibility. We reasoned that the treatment of the genome of YerA41 with the DNAP01 would deliver a product that could be amplified by PCR and then sequenced. Using this approach, we should be able to amplify DNA fragments between adjacent RNA-seq-derived scaffolds by PCR using as a template the genomic material incubated first with DNAP01 plus dNTPs. To this end, primers for PCR were designed pointing outwards from both ends of the nine scaffolds and used initially in all possible combinations. Whenever a PCR-product was obtained its sequence was determined by Sanger sequencing. This way, all the gaps between the scaffolds were filled and the full DNA sequence of the YerA41 genome was obtained resulting in a total genome size of 145,577 bp. The genome assembled into a circular form and our data did not allow detection of physical termini. As the predicted genes were organized in two major blocks with divergent orientation, the genome was therefore re-organized based on the overall organization of the genes such that the forward genes are in the left end and the reverse genes in the right end of the genome (Supplementary Figure S14). The genome contains altogether 213 predicted protein coding and 3 tRNA coding genes. While altogether 42 putative sigma-70 like promoters were identified upstream the predicted genes (Supplementary Table S6), only one rho-independent terminator was detected between the g161 and g162 genes that are the last genes in the forward and reverse oriented gene blocks (Supplementary Figure S14). The annotations of the genes have been described in details earlier (8). Based on several lines of evidence, exemplified by the VIPTree analysis against all sequenced bacteriophages (Supplementary Figure S15), YerA41 is the first representative of a new family of phages. Its morphological features resemble those of jumbo phages infecting both Gram-positive and –negative bacteria.

## DISCUSSION

We describe here a bacteriophage with a genome containing a novel bulky nucleotide modification in its DNA, whilst packaging DNA in its capsid at a lower density compared to other DNA phages. We hypothesised how these two observations are related. The reconstruction of the YerA41 larger morphological variant capsid, YerA41-B, shows it has a T-number of 28 with *laevo* handedness. This differs to previously reported T = 28 capsids, which reported *dextro* handedness, in the cases of 121Q(33), phAPEC6(39) and ΦXacN1(40). Nevertheless, a *laevo* handedness for YerA41-B is supported by the fitting of the canonical HK97 major capsid protein fold. The 131 nm diameter and the T-number of the YerA41-B capsid is consistent with those of jumbophages (33), despite the total length of the YerA41 genome of ca. 146 kb (Supplementary Figure S14) being less than the 200 kb threshold generally considered to define this class. The 33–36 Å spacing between layers of the genome cargo inside the capsid indicates a low density of packing, compared to many lower T-number members of the *Caudovirales* which have values closer to 25 Å (41). This wider spacing and lower packing density is consistent with a large volume for the relatively short genome. The calculated density for the YerA41-B head is, however, markedly low, at ∼0.23 bp/nm^3^, compared to other jumbophages (33). Phage G possesses a T = 52d capsid and a calculated density of 0.39 bp/nm^3^, which is also lower than that of other described jumbophages (33). Phage G does not apparently show layers of genomic material around the inside of its capsid, which likely only arise as the result of DNA close-packing. The presence of visible genome layers inside YerA41-B indicates its density may therefore be higher, and its volume of ∼620 × 103 nm^3^ is indeed sufficient to store at least two copies of the 146 kb genome. This would increase internal density to ∼0.47 bp/nm^3^. Interestingly, the small morphological variant capsid, YerA41-S, could accommodate one full length of the YerA41 genome. Based on the diameter of YerA41-S, its volume will be ∼2.7 × 10^5^ nm^3^, implying a density of ∼0.5 bp/nm^3^ in this instance, which is also within the expected range of capsid densities.

Given that only one copy of the 146 kb genome is presumably required for infection, and terminal redundancy of packaged DNA has not been observed over 35% of the genome length (33), a proportion of the excess storage volume inside YerA41-B is likely filled by the large nucleotide hypermodifications described in this study. In particular, by the glycosubstitution of thymidines. While the calculated thymidine percentage in the YerA41 genome is 34%, the estimated nucleotide percentages of T, dA, dG and dC are 15, 40, 20 and 25%, respectively (Table 1). The values of dA and dG include a little extra due overlapping of the T-substitution in the HPLC. Assuming that, T% should be 34%, it can be estimated that 56% of thymidine residues could be substituted in this way.

The additional storage requirements that result from the glycosubstitution of thymidines can therefore be estimated as follows. The calculated molecular mass of the phage genomic DNA, consisting of 145,577 bp without terminal repeats, is 89.97 × 10^6^ Da, taking 618 Da as the average mass of an unmodified basepair. If all the thymidines present in the 98567 AT base pairs of the genome are fully substituted, this would contribute an additional 1044 Da per AT basepair, and altogether an additional 102.9 × 10^6^ Da per genome. Assuming, however, that 56% of thymidines are modified, this figure is 57.63 × 10^6^ Da. This is 64% percent of the unmodified genome's molecular weight, and as such is a significant addition. Whilst the structure of this modification is yet to be characterised, given its size it likely imposes additional storage requirements on the capsid and the genome arrangement inside, which may be explained by the capsid's large diameter and the wide spacing between genome layers (Figure 1).

Apparently, due to the massive glycosubstituted thymidine in the DNA, YerA41 requires a unique DNA polymerase for replication. Here we carried out the first characterization of DNAP01, the YerA41 DNA polymerase. DNAP01 is packaged inside the mature phage particle (8) indicating that it is required by the phage immediately upon infection. To better understand the behaviour of DNAP01 and its polymerase activity, we expressed and purified the protein and characterised it using in vitro assays. While the His-tagged DNAP01-Ct was almost insoluble, the full length His-tagged DNAP01 was fully soluble, which facilitated in its purification. The folding problem of DNAP01-Ct was probably caused either by the lack of the N-terminal domain or by the lack of an appropriate chaperone in the expression host (42–44).

Importantly, the activity of DNAP01 was used in an assay where the YerA41 genomic material functioned as a template. This far, no other DNA polymerase had been able to replicate it. Not only could DNAP01 use the YerA41 genomic material as template, it accomplished that without any added primers (Figure 3). Incubation of the YerA41 genomic material with T4 DNA polymerase in a similar setting did not generate a template for PCR (Supplementary Figure S16). It is possible that DNAP01 could be a primer-independent DNA polymerase (45). In conclusion, the assays performed in this study clearly demonstrated the DNA polymerase activity of DNAP01 and its capacity to use YerA41 genome as template. Further research is required to characterize the protein and fully understand its possible applications.

In conclusion, we describe here YerA41, a novel type of (pseudo)jumbo phage that requires a jumbo capsid to package its heavily thymidine-modified DNA. YerA41 uses its own DNA polymerase, DNAP01, to replicate its modified genomic DNA. Further studies are due to fully characterize this bacteriophage and the molecular mechanisms involved in its life style.

## DATA AVAILABILITY

Cryo-EM data is available through the Electron Microscopy Data Bank (https://www.ebi.ac.uk/pdbe/emdb/) with accession number EMD-12590, and was deposited with wwPDB (https://www.wwpdb.org). The *E. coli* strain carrying the plasmid pET28a-DNAP01 generated in this study has been deposited to the Skurnik laboratory bacterial strains collection under the storage number #6800. The authors declare that all other data supporting the findings of this study are available within the paper and its supplementary information files. Further information and requests for resources and reagents should be directed to and will be fulfilled by the Lead Contact, Mikael Skurnik.

### ACCESSION NUMBERS

The annotated sequence data of YerA41 has been submitted in GenBank under the accession number MW570730.

## Supplementary Material

gkac203_Supplemental_File
