# Supplementary material for: The DNA polymerase of bacteriophage YerA41 replicates its T-modified DNA in a primer-independent manner
Source: Nucleic Acids Res. 2022 Mar 31;50(7):3985–97. doi: 10.1093/nar/gkac203 (PMC9023294; doi:10.1093/nar/gkac203)
Supplement: gkac203_Supplemental_File [file gkac203_supplemental_file.pdf]

Supplementary data

The DNA polymerase of bacteriophage YerA41 replicates its T-modified DNA in a primer-independent manner

Miguel V. Gomez-Raya-Vilanova[1], Katarzyna Leskinen[1], Arnab Bhattacharjee[1,2], Pasi Virta[3], Petja Rosenqvist[3], Jake L. R. Smith[4], Oliver W. Bayfield[4], Christina Homberger[5], Tobias Kerrinnes[6], Jörg Vogel[5,6,7], Maria I. Pajunen[1], Mikael Skurnik[1,8,*]

[1] Department of Bacteriology and Immunology, Medicum, Human Microbiome Research Program, Faculty of Medicine, University of Helsinki, 00014 UH, Helsinki, Finland
[2] Drug Discovery, Herantis Pharma Ltd. Bertel Jungin Aukio 1, Espoo 02600, Finland
[3] Department of Chemistry, University of Turku, 20014 Turku, Finland
[4] York Structural Biology Laboratory, University of York, YO10 5DD York, United Kingdom
[5] Institute of Molecular Infection Biology (IMIB), University of Würzburg, D-97080 Würzburg, Germany.
[6] Helmholtz Institute for RNA-based Infection Research (HIRI), Helmholtz Centre for Infection Research (HZI), D-97080 Würzburg, Germany.
[7] Faculty of Medicine, University of Würzburg, D-97080 Würzburg, Germany.
[8] Division of Clinical Microbiology, Helsinki University Hospital, HUSLAB, 00290 Helsinki, Finland
* To whom correspondence should be addressed: mikael.skurnik@helsinki.fi; Tel.: +358-50-3360981

Supplementary report

How contaminating ribosomes misled the determination of the nucleotide composition of phage genomic material

Introduction

Bacteriophages often protect their genomic DNA against degradation by host bacterial defence systems such as restriction enzymes by introducing substitutions to nucleotides. To elucidate the nucleotide modifications present in YerA41, genomic material isolated from concentrated phage lysate was processed into nucleosides and the nucleoside composition was determined as described in the main text.

Materials and methods

RNA isolation from phage particles

To an aliquot of purified phage YerA41 lysate in 100 µl SM buffer, containing 10^9 – 10^{12} PFU, 1.3 µL of DNase I (1 U/mL) and 0.5 µL RNase A (10 mg/mL) were added, and the tube was incubated at 37°C for 30 min. Thereafter the total RNA was isolated from the samples using the RNeasy® Mini Kit (Cat no. 74104, Qiagen) starting at the step of bacterial RNA isolation where 350 µl buffer TLE is added to the 100 µl sample. After vortexing the sample, 250 µl absolute ethanol was added to the sample, mixed by pipetting and immediately transferred to an RNeasy mini column. After centrifugation, the bound RNA was washed once with 700 µl buffer RW1, and twice with 500 µl buffer RBE according to the kit instructions. The RNA was eluted into 50 µl RNase free water, and immediately, after removing a 10 µl aliquot for quality control stored on ice, the remaining sample was stored at -70°C.

The RNA concentrations were determined using the Qubit™ RNA High Sensitivity Assay kit (Cat. no. Q32852, Invitrogen, Eugene, Oregon, USA), and the DNA concentrations, using the Qubit™ dsDNA Broad Range Assay kit (Cat. no. Q32850, Invitrogen). The size distribution of the RNA molecules was analysed by TapeStation device (Agilent Technologies, Inc., Santa Clara, CA, USA) at Biomedicum Functional Genomics Unit, University of Helsinki (https://www2.helsinki.fi/en/infrastructures/genome-analysis/infrastructures/biomedicum-functional-genomics-unit-fugu/fugu-genomics-instruments).

Phage lysate RNA concentration measurements

The concentrations of free RNA in phage lysates (10^{12} - 10^{13} PFU/mL) were measured using Qubit™ RNA High Sensitivity Assay kit. Thirty 30 µl of lysate was diluted with 70 µl of SM buffer to obtain untreated test lysate (UTL). A 1:10 dilution of UTL was prepared and a 2 µl aliquot was used to determine its RNA concentration with Qubit. After that either 0.5 or 1 µl of RNase A (10 mg/mL) was added to UTL and incubated at 37 C for 60 min to get RNase treated test lysate (RTL). RNA concentration of RTL was determined directly using a one µl sample. Finally, using RNeasy® Mini Kit, total RNA was isolated from RTL, and the RNA was eluted with 50 µl RNase free water. A 1:10 dilution was prepared immediately from the eluate, and its RNA concentration was measured with Qubit using a 2 µl aliquot. The measurements were then used to calculate the corresponding RNA concentrations present in the original undiluted phage lysates.

RNA-sequencing

RNA quality was checked using the Bioanalyzer RNA 6000 Nano kit (Agilent). cDNA libraries were prepared from 100 ng of total RNA. After fragmentation for 5 min using Mg ions the RNA was treated with T4 PNK for phosphorylation/dephosphorylation and RppH for decapping followed by NEBNext® Multiplex Small RNA Library Prep. (New England Biolabs). rRNA depletion was not performed. The number of the PCR cycles was determined to 12 by qPCR and the elongation time was set to 30 sec. Libraries were quantified before and after pooling by Qubit™ dsDNA HS Assay Kit (3.0 Fluorometer; ThermoFisher) and quality was checked using Bioanalyzer High Sensitivity DNA kit (Agilent). Sequencing of pooled libraries, spiked with 1% PhiX control library, was performed in single-end mode with 75 cycle High Output Kit v2.5 on the NextSeq 500 platform (Illumina). Demultiplexed FASTQ files were generated with bcl2fastq2 v2.20.0.422 (Illumina).

To assure high sequence quality, Illumina reads were quality- and adapter-trimmed via Cutadapt (1) v2.5 using a cutoff Phred score of 20 in NextSeq mode, and reads without any remaining bases were discarded (command line parameters:

--nextseq-trim=20 -m 1 -a AGATCGGAAGAGCACACGTCTGAACTCCAGTCAC

Afterwards, we applied the pipeline READemption (2) v0.4.5 to align all reads longer than 11 nt (-l 12) to the *Yersinia ruckeri* strain CSF007-82 (NCBI Acc.-No: LN681231.1, LN681229.1, LN681230.1) as well as Yersinia phage YerA41 (NCBI Acc.-No: MW570730) genomes using segemehl v0.2.0 (3) with an accuracy cut-off of 95% (-a 95). We applied READemption to generate coverage plots representing the numbers of mapped reads per nucleotide. Here, we used sequencing depth-normalized plots from output folder coverage-tnoar_min_normalized for visualization in the genome browser. We used READemption gene_quanti to quantify aligned reads overlapping genomic features by at least 10 nts (-o 10) on the sense strand (-a). For this, we applied CDS, rRNA and tRNA annotations from NCBI for the bacterium and for the phage.

RNase A and DNase I treatment of YerA41 genomic material

RNase A treatment was performed in samples with 30 µL final volume. Three µL of 10x DNAse buffer (Promega, USA) and 1.5 µL of RNase A enzyme were added to a mixture of 15.5 µL of nuclease-free water and 10 µL of YerA41 genomic material (concentration of 50 ng/µL). The mixture was incubated for 30 min at 37ºC and then stored at -70ºC. The DNase I treatment was carried out with the same protocol with 1U of Promega's RQ1 RNase-Free DNase (Cat.# M6101).

Results

Nucleoside composition of YerA41 genomic material isolated from conventional phage stock

The YerA41 genomic material, isolated by phenol-chloroform extraction as described in the main text, was digested using the Nucleotide digestion mix, and analyzed by chromatography and mass spectrometry to elucidate the nucleoside composition (**Figure I panel C**). Interestingly, apart from the canonical deoxyribonucleosides, deoxyadenosine (dA), deoxyguanosine (dG), deoxycytosine (dC) and thymidine (T) present in DNA, ribonucleosides adenosine (A), guanosine (G), cytosine (C) and uridine (U), usually present in RNA molecules, were also found (**Figure I panel C**).

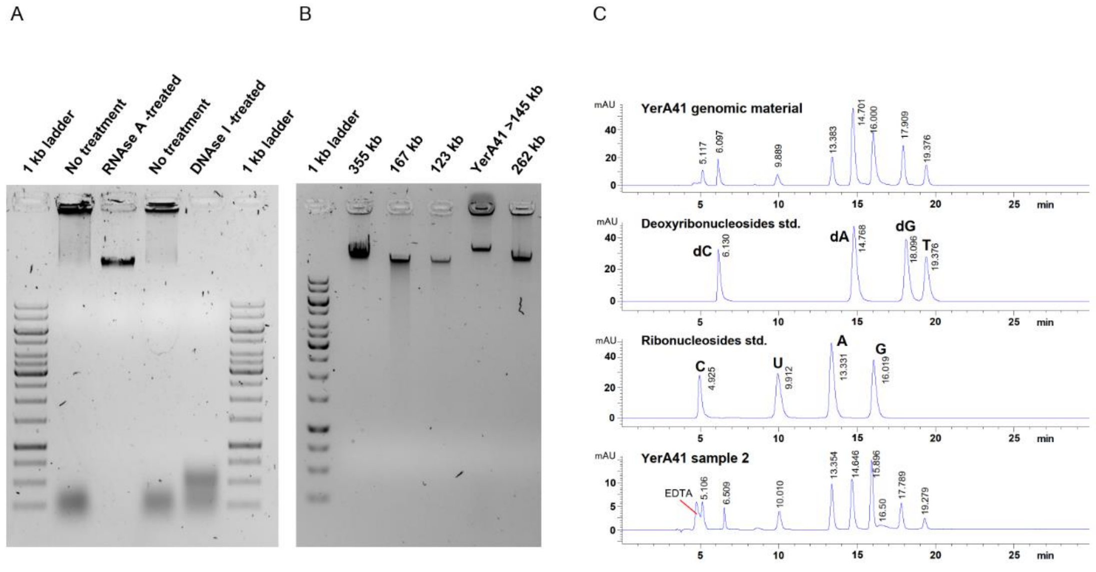

Figure I. *Analysis of the genomic material of phage YerA41.* ***Panels A and B.*** *1% agarose gel electrophoresis analysis of phage YerA41 genome samples without and with RNase A or DNAse I treatment (panel a), and in comparison to other phage DNA samples of different sizes (355 kb, fHe-Yen9-04, acc.no LT960551; 167 kb, ϕR1-RT, acc.no HE956709; 123 kb, ϕR2-01, acc.no HE956708; 262 kb, ϕR1-37, acc.no AJ972879) (panel b).* ***Panel C.*** *HPLC analyses of two independently isolated genomic material of YerA41 digested into nucleosides compared with ribonucleoside and deoxyribonucleoside standards. UV detection wavelength = 260 nm.*

The phage particles were RNase-treated prior to the genomic material was isolated, however, that would not exclude the possibility of some RNA contamination in the samples. The analysis was repeated from another batch of phage with almost identical results. In addition, we have used identical preparation methods to genomic materials of other phages and never seen even traces of NTPs in the samples, thus these results indicated that RNA forms part of the genomic material of YerA41. Prompted by this finding, we treated the genomic material with RNAse A and observed a change in the migration profile in the agarose gel electrophoresis (**Figure I panel A**). The gel demonstrates well the influence of both DNase I and RNase A on the genomic material. The RNase A -treatment released the DNA to migrate from the wells into the gel, and at the same time degraded the small RNA species that migrated in the front of the gel. Very interestingly, the DNase I -treatment, in addition to degrading the DNA, released new RNA material that migrated slower than the intact small RNA in the gel. While nothing conclusive could be said on the size distribution of the released RNA molecules based on the DNA gel, they migrated faster than rRNA in similar DNA gels, so it was safe to conclude that the majority of these RNA molecules were likely below 1000 nt in length.

In light of the above results, the RNA and DNA concentrations of genomic materials isolated from phage YerA41 and from a control DNA phage using the phenol-chloroform extraction protocol were determined using the Qubit assay kits that allow independent measurement of both types of nucleic acids. The results (**Table I**) showed that consistently RNA was present in the YerA41 genomic material in equal quantity to DNA. On the contrary, for the control phage fEin-Pmi01, the amount of RNA was below 10 % from that of DNA.

Table I. *RNA and DNA contents of genomic material isolated from phage YerA41 lysates using the phenol-chloroform (Ph/Chl) extraction method and the the RNeasy kit protocol. Phage fEin-Pmi01 lysate was used as a control for Ph/Chl extraction.*

Sample	PFU / ml	Sample volume	Method	Final susp. vol.	RNA ng/µl	Total RNA ng	RNA $ng/10^{10}$ PFU	Extrapolated RNA conc in original lysate (µg/mL)	DNA ng/µl[a]	Total DNA ng[a]	DNA $ng/10^{10}$ PFU
YerA41 b-2	$3.95*10^{12}$	400 µl	Ph/Chl	100 µl	19.2	1920	12.5	4.8	16.2	1620	10.3
YerA41 b-3	$3.95*10^{12}$	400 µl	Ph/Chl	100 µl	20.8	2080	13.2	5.2	17.0	1700	10.8
YerA41 d	$3.0*10^{13}$	400 µl	Ph/Chl	200 µl	24.3	4860	4.1	12.15	31.3	6260	5.2
fEin-Pmi01	$1.25*10^{11}$	400 µl	Ph/Chl	50 µl	2.80	140	28	0.35	29.8	1490	298
YerA41 b-11	$3.95*10^{12}$	1200 µl	Ph/Chl	300 µl	37.2	11160	23.5	9.3	35.1	10530	22.2
YerA41 b-12	$3.95*10^{12}$	1200 µl	Ph/Chl	300 µl	39.2	11760	24.8	9.8	38.7	11610	24.5
YerA41 b	$3.95*10^{12}$	100 µl	RNeasy	50 µl	45.0	2250	57.0	22.5	<<	<<	<<
YerA41 d-1	$3.0*10^{13}$	100 µl	RNeasy	50 µl	430	21500	71.7	215	1.96	98	0.33
YerA41 d-1	$3.0*10^{13}$	30 µl	RNeasy	50 µl	640	32000	355	1067	n.m.	n.m.	--
YerA41 d-2	$3.0*10^{13}$	30 µl	RNeasy	50 µl	230	11500	128	383	n.m.	n.m.	--
YerA41 d-3	$3.0*10^{13}$	30 µl	RNeasy	50 µl	323	16150	179	538	n.m.	n.m.	--

[a] <<, under detection limit; n.m., not measured

To carry out further the RNA analysis, total RNA was extracted directly from the phage particles using the RNeasy mini kit (**Table I**). Surprisingly, the RNA yield was consistently very high, at best 355 ng / 10^{10} PFU. When compared to the total amount of RNA present in the phenol-chloroform extracted genomic material, the yields in the latter were >10-fold smaller, at best 24.8 ng / 10^{10} PFU, indicating that a majority of RNA was lost during the phenol extraction. The same apparently happened also to DNA as the calculated mass of the 145 kb phage genomic DNA would be 1600 ng / 10^{10} PFU, and the best yield was only 24.5 ng / 10^{10} PFU (**Table I**). It was very likely that the isolation methods should be further optimized for quantitative recovery of the genomic material.

An aliquot of the isolated RNA from lysate d was analysed using the TapeStation instrument to get an idea of the size distribution of the molecules (**Figure II**). While most of the RNA molecules were of 50 – 200 nucleotides in size, the presence of longer, up to 6000 nt long molecules were detected.

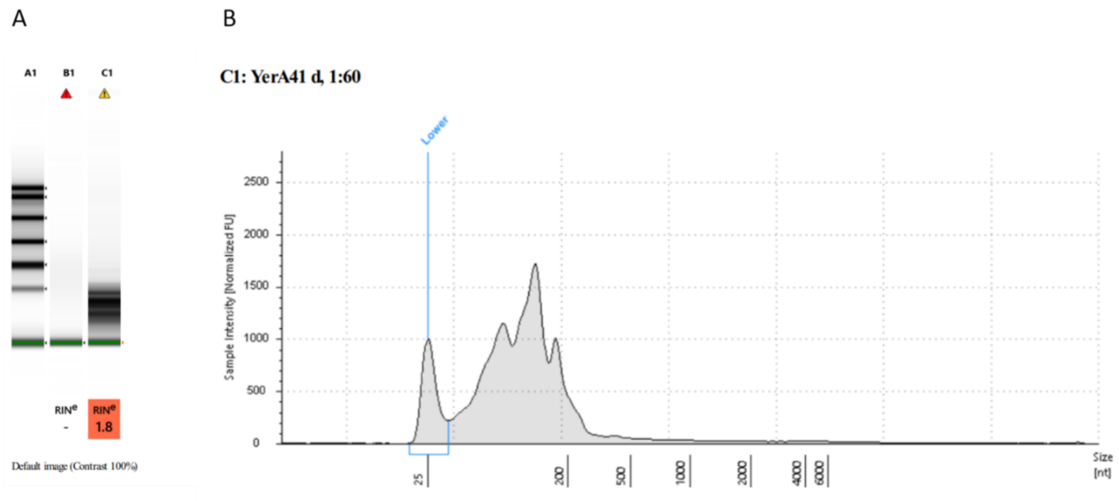

Figure II. *Size distribution of RNA molecules isolated from phage particles using the Qiagen RNeasy Mini Kit.* ***Panel A.*** *The digital TapeStation image of the analysed samples.* ***Lane A1,*** *size standards (from top, 6000, 4000, 2000, 1000, 500, 200 and 25 nucleotides);* ***Lane B1,*** *YerA41 RNA sample M (too dilute for analysis);* ***Lane C1,*** *YerA41 RNA sample d1(1:60 diluted).* ***Panel B.*** *The sample intensity graph of the YerA41 RNA sample d1.*

The RNA samples isolated from lysates b and d (**Table I**) were submitted to RNA-sequencing. The sequence reads were aligned to the reference genomes of both the host bacterium *Y. ruckeri* PB-H2 chromosome (Acc.no. LN681231.1) plus its plasmids pYR2 (Acc.no. LN681229.1) and pYR3 (Acc.no. LN681230.1), and that of phage YerA41 (Acc. no MW570730). The results surprisingly revealed that >90% of the RNA-reads aligned to the rDNA operons of the host bacterium (**Figure III**).

A

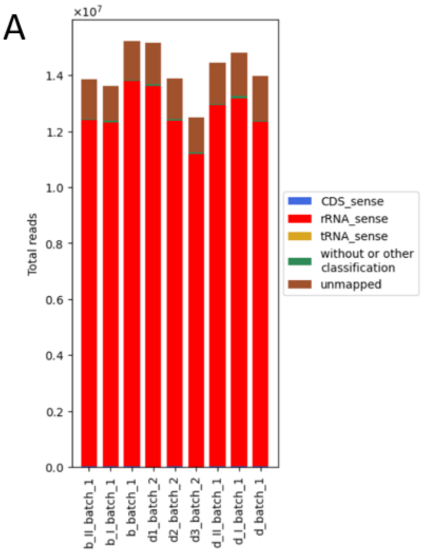

Mapping Stats:

Reference: various
Mapper: READemption version: 0.4.5
Counting: READemption version: 0.4.5

Sample ID	Total reads	Aligned reads	Multimapped reads	Unique reads	Unmapped reads	Number of splices
b_II_batch_1	13849538	12441976	12308701	133275	1407562	0
b_I_batch_1	13632262	12362241	12211310	150931	1270021	0
b_batch_1	15224338	13832761	13704251	128510	1391577	0
d1_batch_2	15163515	13676822	13528318	148504	1486693	0
d2_batch_2	13886005	12439430	12303773	135657	1446575	0
d3_batch_2	12488220	11232594	11113749	118845	1255626	0
d_II_batch_1	14435798	12968263	12826396	141867	1467535	0
d_I_batch_1	14796778	13249925	13088952	160973	1546853	0
d_batch_1	13975340	12368113	12232696	135417	1607227	0

Absolute:

Sample ID	CDS_sense	rRNA_sense	tRNA_sense	without or other classification	unmapped
b_II_batch_1	21450.47	12374804.49	685.61	45035.43	1407562.00
b_I_batch_1	21158.42	12281181.54	1325.46	58575.58	1270021.00
b_batch_1	19510.47	13781883.59	608.70	30758.24	1391577.00
d1_batch_2	19318.62	13593479.12	965.83	63058.43	1486693.00
d2_batch_2	21345.62	12362090.40	1377.88	54616.10	1446575.00
d3_batch_2	16722.03	11172868.93	680.76	42322.28	1255626.00
d_II_batch_1	21214.57	12908156.88	504.48	38387.06	1467535.00
d_I_batch_1	21155.34	13164005.52	946.42	63817.71	1546853.00
d_batch_1	20011.47	12309959.83	813.55	37328.15	1607227.00

B

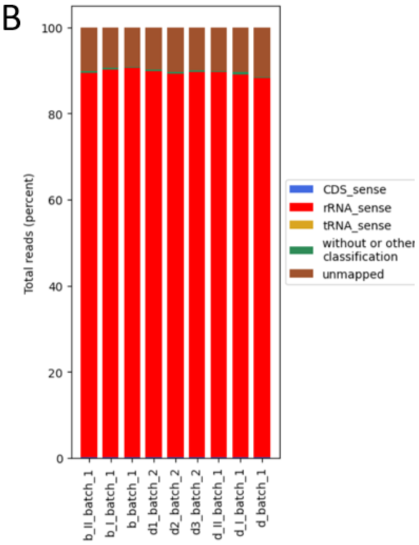

Percentage:

Sample ID	CDS_sense	rRNA_sense	tRNA_sense	without or other classification	unmapped
b_II_batch_1	0.15	89.35	0.00	0.33	10.16
b_I_batch_1	0.16	90.09	0.01	0.43	9.32
b_batch_1	0.13	90.53	0.00	0.20	9.14
d1_batch_2	0.13	89.65	0.01	0.42	9.80
d2_batch_2	0.15	89.03	0.01	0.39	10.42
d3_batch_2	0.13	89.47	0.01	0.34	10.05
d_II_batch_1	0.15	89.42	0.00	0.27	10.17
d_I_batch_1	0.14	88.97	0.01	0.43	10.45
d_batch_1	0.14	88.08	0.01	0.27	11.50

C

Sample ID	Detected genes	Genes below threshold	Total number of reads
b_II_batch_1	320	3524	13849538
b_I_batch_1	300	3544	13632262
b_batch_1	270	3574	15224338
d1_batch_2	293	3551	15163515
d2_batch_2	303	3541	13886005
d3_batch_2	258	3586	12488220
d_II_batch_1	302	3542	14435798
d_I_batch_1	304	3540	14796778
d_batch_1	290	3554	13975340

Figure III. *Summary of RNA-sequencing data on RNA isolated from YerA41 lysates b and d analysed in 3 replicates.* ***Panel A.*** *Absolute read numbers showing the read mapping statistics against the reference genomes of the host bacterium* Y. ruckeri *PB-H2 chromosome (Acc.no. LN681231.1) and its plasmids pYR2 (Acc.no. LN681229.1) and pYR3 (Acc.no. LN681230.1) and that of phage YerA41 (Acc. no MW570730). Reads that mapped to multiple locations equally well (e.g. rRNA reads) were divided equally amongst potential mapping locations. The graph shows the distribution of the reads among the different target categories.* ***Panel B.*** *Distribution of the read numbers as percentages among the different target categories.* ***Panel C.*** *Number of expressed protein coding genes identified (>10 reads mapped)*

As there could be a possibility that the rRNA specific reads could originate from a minimal contamination originating from the host cell cytoplasm, we determined how much RNA is present external the phage particles in the lysate, and how efficiently the RNase A treatment included in the isolation protocol reduced this external RNA. The results of two independent experiments carried out with lysates b and d are presented in **Table II**. To enable comparisons between the samples, their RNA concentrations were calculated back to undiluted original phage lysates.

Table II. *Qubit™ RNA High Sensitivity Assay kit measurements of RNA concentrations of (i) YerA41 lysates before and after RNase A treatments and of (ii) RNeasy Mini Kit isolated RNA. Shown are the results of two independent experiments. Samples contained 30 µL of original lysate plus 70 µL of added SM-buffer. The measurements were carried out on appropriately diluted aliquots withdrawn from untreated lysate samples, from samples after 60 min incubation with 5 or 10 µg of RNase A (yellow shading), and on RNA isolated from the RNase A-treated samples (grey shading).*

Phage lysate	PFU / mL	RNase A (µg)	Aliquot (µL)	Sample			RNA concentration (µg/mL) of lysate	Comments
				Total or elution vol (µL)	RNA (ng/µL)	Total RNA (ng)		
**Experiment 1**								
b	$4*10^{12}$	0	30	100	330	33,000	1,100	Contaminating RNA
d	$3*10^{13}$	0	30	100	610	61,000	2,030	Contaminating RNA
b	$4*10^{12}$	5	30	100	10.0	1,000	33 (3.0%)	Remaining contaminating RNA
d	$3*10^{13}$	5	30	100	25.4	2,540	85 (4.2%)	Remaining contaminating RNA
b	$4*10^{12}$	5	30	50	198.5	9,925	330.8	RNeasy isolated RNA
d	$3*10^{13}$	5	30	50	486	24,300	810.0	RNeasy isolated RNA
**Experiment 2**								
b	$4*10^{12}$	-	30	100	280	28,000	933	Contaminating RNA
d	$3*10^{13}$	-	30	100	216	21,600	720	Contaminating RNA
b	$4*10^{12}$	10	30	100	9.4	9,400	31 (3.3%)	Remaining contaminating RNA
d	$3*10^{13}$	10	30	100	8.19	8,190	27 (3.8%)	Remaining contaminating RNA
b	$4*10^{12}$	10	30	50	121	6,050	201.5	RNeasy isolated RNA
d	$3*10^{13}$	10	30	50	135	6,750	225.0	RNeasy isolated RNA

The following conclusions could be drawn from these experiments.

- the Qubit RNA HS kit apparently measured only the free RNA (external to the phage particles).
- RNase A treatment was able to reduce the external free RNA concentration of the lysate by 96-97 % during the 60 min incubation.
- there was approximately 10-fold more RNA protected against RNase A that could be isolated using the RNeasy kit from the RNase A -treated samples.

These results, related to the RNA-sequencing results strongly indicated that majority, >90%, of the sequence reads obtained (**Figure III**) originated from the protected RNA released during the RNeasy Mini kit purification protocol. As ribosomal rRNA is transcribed initially as a 5,500-6,000 nt long primary transcript that is gradually processed by several different RNases into 23S, 16S, 5S and several tRNA species (4), logistically, if phage indeed would package rRNA into capsids it should be the primary unprocessed transcript. In such a case one should detect an even read coverage over the whole rDNA operon including the rRNA intergenic regions and on the tRNA genes present in the rDNA operons. Inspection of the alignment of the sequence reads on the rDNA operon revealed that they did not cover the operon evenly (**Figure IV**). No reads were detected on the intergenic regions and within the 16S and 23S rRNA encoding genes there were distinct regions without any read coverage. It looked like these parts of rRNA had been accessible to RNase A degradation as if the rRNA molecules had resided inside intact ribosomal structures known to have surface-exposed rRNA loops.

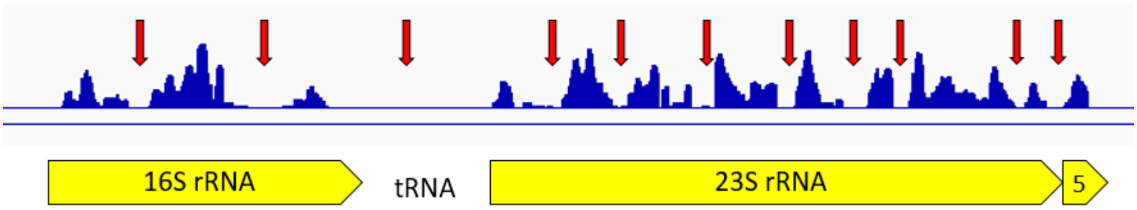

Figure IV. *RNA-sequencing read coverage over a rDNA operon of* Y. ruckeri. *Shown are the locations of the 16S rRNA, tRNA, 23S rRNA and 5S rRNA encoding genes. The regions missing read coverage are indicated by the red arrows.*

Thus it could be possible that the purified phage lysate might contain intact ribosomes. This was indeed visualized in transmission electron microscopy on CsCl-gradient purified phage particles (**Figure V**) showing that the phage particles are accompanied by particulate and fibrous material.

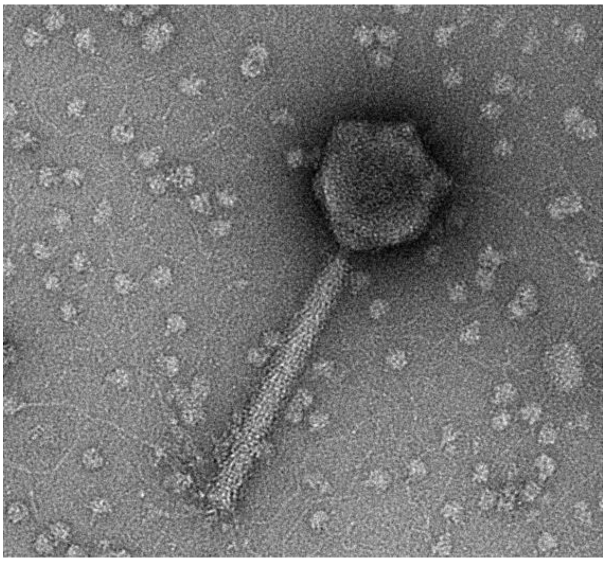

Figure V. *Transmission EM micrograph on phage YerA41 virion surrounded by particles of ca 20 nm in diameter and some fibrous material.*

We previously determined the proteome of the phage particle (5), however, the LC-MS/MS data was not analysed for the presence of the phage host proteins. This analysis revealed abundant presence of ribosomal proteins in the phage sample purified by ultracentrifugation. There were 31 large and 21 small subunit ribosomal proteins among 87 proteins with highest identification scores (**Table III**) . Also the flagellar protein FliC was abundant, possibly the the fibrillar material in **Figure V**. In addition, several enzymes and RNAP beta and beta' subunits were present in the sample. In conclusion, the rRNA and the ribonucleosides detected in the sample (**Figure I panel C**) have to be considered as artefacts due to protection of the rRNA within ribosomes from RNase A.

Table III. *LC-MS/MS analysis of phage YerA41 host proteins in purified phage stock sample. LSU, ribosome large subunit; SSU, ribosome small subunit.*

Description	Score	Coverage %	# Unique Peptides
CSF007_11070 Flagellar biosynthesis protein FliC	388,51	83,68	17
CSF007_1425 LSU ribosomal protein L24p (L26e)	357,68	82,69	11
CSF007_11080 Flagellar biosynthesis protein FliC	308,68	83,53	13
CSF007_1385 LSU ribosomal protein L2p (L8e)	280,98	67,15	21
CSF007_1430 LSU ribosomal protein L5p (L11e)	252,80	86,03	20
CSF007_16660 DNA-directed RNA polymerase beta subunit	243,02	46,94	54
CSF007_16655 DNA-directed RNA polymerase beta' subunit	239,18	42,75	53
CSF007_2055 LSU ribosomal protein L9p	229,28	82,00	15
CSF007_3330 Dihydrolipoamide acetyltransferase component of pyruvate dehydrogenase complex	217,75	37,36	28
CSF007_1375 LSU ribosomal protein L4p (L1e)	199,15	85,07	18
CSF007_16675 LSU ribosomal protein L1p (L10Ae)	189,66	64,10	18
CSF007_1455 SSU ribosomal protein S5p (S2e)	188,83	86,83	15
CSF007_11945 LSU ribosomal protein L25p	158,81	78,72	7
CSF007_1370 LSU ribosomal protein L3p (L3e)	150,33	48,80	13
CSF007_1400 SSU ribosomal protein S3p (S3e)	148,13	63,36	20
CSF007_9590 Pyruvate kinase	139,28	59,73	20
CSF007_1340 SSU ribosomal protein S7p (S5e)	132,64	73,08	15
CSF007_0085 ATP synthase beta chain	130,04	52,17	19
CSF007_1440 SSU ribosomal protein S8p (S15Ae)	129,70	76,92	14
CSF007_1490 SSU ribosomal protein S4p (S9e)	126,19	50,00	14

CSF007_1395 LSU ribosomal protein L22p (L17e)	124,36	70,91	14
CSF007_14275 SSU ribosomal protein S2p (SAe)	115,26	77,59	19
CSF007_0075 ATP synthase alpha chain	114,93	47,17	22
CSF007_16670 LSU ribosomal protein L10p (P0)	110,96	82,63	14
CSF007_1445 LSU ribosomal protein L6p (L9e)	106,01	79,10	14
CSF007_2040 SSU ribosomal protein S6p	102,33	76,15	12
CSF007_1405 LSU ribosomal protein L16p (L10e)	100,78	60,29	10
CSF007_17300 Transcription termination factor Rho	96,45	45,82	20
CSF007_7025 Chromosome partition protein MukB	94,99	23,75	23
CSF007_1485 SSU ribosomal protein S11p (S14e)	93,86	72,09	12
CSF007_1465 LSU ribosomal protein L15p (L27Ae)	93,83	70,14	15
CSF007_15860 LSU ribosomal protein L13p (L13Ae)	91,79	80,28	15
CSF007_4120 SSU ribosomal protein S16p	89,89	65,85	5
CSF007_8740 DNA topoisomerase I	85,77	30,38	20
CSF007_2050 SSU ribosomal protein S18p @ SSU ribosomal protein S18p, zinc-independent	82,27	70,67	9
CSF007_1420 LSU ribosomal protein L14p (L23e)	81,00	78,05	12
CSF007_1410 LSU ribosomal protein L29p (L35e)	79,13	63,49	7
CSF007_5820 Dihydrolipoamide succinyltransferase component (E2) of 2-oxoglutarate dehydrogenase complex	78,87	35,06	13
CSF007_2910 SSU ribosomal protein S20p	78,32	45,98	6
CSF007_1480 SSU ribosomal protein S13p (S18e)	73,44	73,73	16
CSF007_2180 LSU ribosomal protein L27p	70,66	55,29	5
CSF007_1365 SSU ribosomal protein S10p (S20e)	69,23	66,99	11
CSF007_8495 Alcohol dehydrogenase-Acetaldehyde dehydrogenase-Pyruvate-formate-lyase deactivase	67,31	32,36	21
CSF007_2440 Cytosol aminopeptidase PepA	65,30	40,36	16
CSF007_7340 LSU ribosomal protein L32p	58,14	63,64	7
CSF007_1335 SSU ribosomal protein S12p (S23e)	57,74	54,84	5
CSF007_1775 Heat shock protein 60 family chaperone GroEL	56,92	39,67	17
CSF007_1500 LSU ribosomal protein L17p	56,64	55,47	9
CSF007_4135 LSU ribosomal protein L19p	55,58	57,63	8
CSF007_6950 SSU ribosomal protein S1p	54,43	35,19	14
CSF007_16680 LSU ribosomal protein L11p (L12e)	54,38	42,96	8
CSF007_6500 6-phosphogluconate dehydrogenase, decarboxylating	52,88	47,55	16
CSF007_1460 LSU ribosomal protein L30p (L7e)	51,16	76,27	8
CSF007_1435 SSU ribosomal protein S14p (S29e)	51,15	54,46	8
CSF007_1380 LSU ribosomal protein L23p (L23Ae)	50,82	37,00	4
CSF007_1450 LSU ribosomal protein L18p (L5e)	50,00	55,56	4
CSF007_5805 Succinate dehydrogenase flavoprotein subunit	49,37	30,44	13
CSF007_17815 LSU ribosomal protein L28p	48,57	47,44	8
CSF007_1495 DNA-directed RNA polymerase alpha subunit	47,58	55,93	17
CSF007_5810 Succinate dehydrogenase iron-sulfur protein	44,85	59,66	10
CSF007_2280 SSU ribosomal protein S15p (S13e)	44,50	67,42	8
CSF007_9200 ATP-dependent RNA helicase HrpA	43,69	13,36	12
CSF007_9825 LSU ribosomal protein L20p	42,50	38,14	8
CSF007_1390 SSU ribosomal protein S19p (S15e)	41,89	53,06	4
CSF007_15530 SSU ribosomal protein S21p	41,88	46,48	7
CSF007_1415 SSU ribosomal protein S17p (S11e)	39,43	55,95	6
CSF007_5885 peptidoglycan-associated outer membrane lipoprotein	38,64	56,89	10
CSF007_3710 3-oxoacyl-[acyl-carrier protein] reductase	38,46	43,75	7
CSF007_15855 SSU ribosomal protein S9p (S16e)	37,25	40,77	6
CSF007_4295 Na(+)-translocating NADH-quinone reductase subunit A	36,51	24,38	8
CSF007_2895 Chaperone protein DnaJ	35,99	36,34	10
CSF007_14120 Methionine ABC transporter substrate-binding protein	34,24	53,56	9

CSF007_9830 LSU ribosomal protein L35p	33,56	43,08	3
CSF007_13735 ATP-dependent RNA helicase SrmB	33,54	25,17	10
CSF007_7135 Outer membrane protein A precursor	32,49	27,89	8
CSF007_4710 Peptidyl-prolyl cis-trans isomerase PpiD	28,74	15,63	7
CSF007_0080 ATP synthase gamma chain	27,71	37,02	9
CSF007_4705 DNA-binding protein HU-beta	26,95	47,78	4
CSF007_15400 Topoisomerase IV subunit A	25,84	12,42	8
CSF007_13275 Polyphosphate kinase	25,58	16,74	9
CSF007_2175 LSU ribosomal protein L21p	24,31	34,95	4
CSF007_9810 Integration host factor alpha subunit	24,26	46,94	7
CSF007_12680 3-ketoacyl-CoA thiolase	24,06	22,25	7
CSF007_15045 hypothetical protein	23,59	21,74	3
CSF007_12210 DNA gyrase subunit A	23,27	12,60	8
CSF007_16595 DNA-binding protein HU-alpha	21,59	66,67	5
CSF007_17810 LSU ribosomal protein L33p	21,49	41,82	3

References

1. Martin,M. (2011) Cutadapt removes adapter sequences from high-throughput sequencing reads. *EMBnet j.*, **17**, 10.

2. Forstner,K.U., Vogel,J. and Sharma,C.M. (2014) READemption--a tool for the computational analysis of deep-sequencing-based transcriptome data. *Bioinformatics*, **30**, 3421–3423.

3. Hoffmann,S., Otto,C., Kurtz,S., Sharma,C.M., Khaitovich,P., Vogel,J., Stadler,P.F. and Hackermüller,J. (2009) Fast Mapping of Short Sequences with Mismatches, Insertions and Deletions Using Index Structures. *PLoS Comput Biol*, **5**, e1000502.

4. Magdalena Kaczanowska and Ryden-Aulin,M. (2007) Ribosome Biogenesis and the Translation Process in Escherichia coli. *Microbiology and Molecular Biology Reviews*, **71**, 477–494.

5. Leskinen,K., Pajunen,M.I., Gomez-Raya Vilanova,M.V., Kiljunen,S., Nelson,A., Smith,D. and Skurnik,M. (2020) YerA41, a Yersinia ruckeri Bacteriophage: Determination of a Non-Sequencable DNA Bacteriophage Genome via RNA-Sequencing. *Viruses*, **12**, 620.

Supplementary Tables

Table S1. *Compositions of media and solutions*

Media or solution	Ingredients and concentration
Kanamycin	100 µg/ml
Chloramphenicol	20 µg/ml
Ampicillin	100 µg/ml
Elution buffer	50 mM NaH_2PO_4; 300 mM NaCl, 250 mM imidazole, pH 8.0
Glycerol	10% (v/v) BD (VWR) in nuclease-free water
Guanidine hydrochloride buffer	GuHCl, 100 mM NaH2PO4, 10 mM Tris-Cl and 6M GuHCl, pH 8.0
Lysis buffer	50 mM NaH_2PO_4, 300 mM NaCl, 10 mM imidazole, pH 8.0
LB	10 g/l Tryptone, 5 g/l Yeast extract, 10 g/l NaCl, pH 7.0
Lysogenic Agar	LB + 15 g/l Bacto-agar
Refolding buffer	50 mM HEPES pH 8.0; 1M GuHCl; 0.06% (w/v) PEG 4000; 0.1% (v/v) NP40; 1 mM DTT
SDS	10% (w/v) in milli-Q water
SDS running buffer	0.1% (w/v) SDS, 248 mM Tris, 1.9M Glycine
SDS sample buffer	50 mM Tris pH 6.8, 2% (w/v) SDS, 0.1% (w/v) Bromophenol blue, 10% (v/v) glycerol, 100 mM DTT
Soft Agar medium	LB + 0.4 % (w/v) agar
SOB	20 g/l Tryptone, 5 g/l Yeast extract, 10 mM NaCl, 2.5 mM KCl, pH 7.0
SOB with Catabolite repression	SOB + 20 mM Mg^{2+} + 20 mM glucose
TM-buffer	50 mM Tris, pH 7.5; 10 mM $MgSO_4$
TM-buffer with 5% or 40% sucrose	TM-buffer + 5% or 40% sucrose (w/v)
SM-buffer	100 mM NaCl; 10 mM $MgSO_4$; 50 mM Tris-HCl, pH 7.5; 0.01% gelatin (w/v)
TAE acid buffer	40 mM Tris, 1 mM EDTA pH 8.0, 20 mM acetic acid
Urea lysis buffer	8 M urea, 0.1 M NaH_2PO_4, 0.01 M Tris/HCl, pH8.0
Wash buffer	50 mM NaH_2PO_4, 300 mM NaCl, 20 mM imidazole, pH 8.0

Table S2. *YearA41 specific primers for PCR and Sanger sequencing.*

**Primer**	**Location in YerA41 genome**	**Sequence 5' to 3'**	**Purpose**
A5_72R	264-283	TGATCCTCGCAAATCAGCAG	PCR and sequencing primers to join the RNA-seq scaffolds
A5_11329F	11539-11558	CCTATGGAGCTCCGTGAGTC	
A3b_99R	13164-13144	ACGACTATTACTGAATGCAAG	
A3_206R	13892-13871	TCCCATGAGGAGTTTCTACAGG	
A3_26441F	40127-40146	GCAATGGGTCACATGTTCCC	
A2b_127R	40399-40378	TCATTAAGAATAGGGAGTCACC	
A2_27191F	44491-44510	ACATTTCCGAGGGAGCTGTC	
A4_80R	44811-44792	CGCACTGTTCACCTGATTGA	
A4_14995F	59726-59747	TCGATATCAAGGCAGCTCTCTT	
A6_212R	60087-60067	GGCTCACCATTCTCTATGCAA	
A6_8058F	67931-67954	TGGTTGATGATTCAGTACTTCGTG	
A10_213R	68380-68357	TCAGAAATTGGTTGGATTCCACAA	
A10_2336F	70503-70524	GACAACTGGTCACAAAGCTACT	
A7_89R	70721-70701	AGCACTCCTGAGAATGCTACT	
A7_5103F	75735-75755	(A)GGATTTCTGGGTCGAAGTCC	
A2_74R	75976-75957	GGAATCAACGAACAACCCCG	
A8_59R	98884-98863	AGTGCTTAGCTAGAAATGCTCT	
A8_3489F	102314-102334	TATGCGTGATGAAGCTCCACA	
A1_42874F	102559-102536	TGAAGTGAAAGTACCTTCATTGGA	
A1_290R	145143-145163	TACTTTCAGCTACGTCACGGA	
Yqp08_qPCRLfor	108134-108115	TTAGGTCAAGGAGTGGGTGA	*g171c* primers for PCR
Yqp08_qPCRLrev	107487-107506	TCCAGTCCATGATCCAGCAA	
gp8F	107506-107487	TTGCTGGATCATGGACTGGA	
gp8R	107297-107316	CTCGTCCATTATCCCACCCA	
Yqp37_qPCRLfor	134247-134228	CCTGCAATCCCAGTACTCCA	*g200c* primers for PCR
Yqp37_qPCRLrev	133727-133746	TTACTGGCAGGAATGGAGCA	
gp37R	134090-134071	GCAACACCTGAAGTCTGCTC	
DNAP1Ct_qPCRLfor	88385-88405	TCAATGCTAACCCGAACTTCA	*g144* primers for PCR
DNAP1Ct_qPCRLrev	89003-88984	CCTCACGGAATCTACAATCAGG	
DNAP1qR	88650-88629	AGCATCATGAACGAATCCAAGT	
DNAP02_qPCRLfor	59328-59349	TCGTGATAAACAACTTGCTGGT	*g113* primers for PCR
DNAP02_qPCRLrev	59746-59726	AGAGAGCTGCCTTGATATCGA	
DNAP2qF	59461-59482	TGGATTCAACGAAAAGCATTGC	
DNAP03_qPCRLfor	99476-99496	TGACAGGGATTGGTGAATTGA	*g158* primers for PCR
DNAP03_qPCRLrev	99969-99950	ATCAGTTCCGACAATCCCGT	
DNAP3qF	99633-99655	TGGATCTATGAGAAGAGGTTGCT	
DNAP3qR	99932-99910	CCTTTGGACTTAGCAATACCTCT	

Table S3. *Controls used for the PCR of the functional assay.*

**Reaction**	**Type of control**	**Comments**
**No DNAP01**	Negative control, no DNAP01, only the untreated YerA41 DNA as template	The untreated phage DNA contains modifications that supposedly make impossible for the thermostable DNA polymerase to amplify the strand.
**cDNA**	Positive control	The cDNA that results from the reverse transcription of the RNA transcripts isolated from YerA41-infected host bacteria. The targeted DNA fragments of YerA41 are easily amplified by PCR.
**No YerA41 DNA**	Only DNAP01 added to check for residual plasmid control (positive).	There may be some residual *dnap01* containing plasmid, even after the purification of the protein, which can produce a false positive with *dnap01*-specific primers.
**Water**	PCR control (negative)	To test if there is some contamination in the PCR.
**Primers**	Primer control	To test the role of the random hexamer primers, YerA41 specific primers or no primers

Table S4. *Overview of the YerA41 gene products showing homology to DNA polymerases.*

Gene	Location	bp	Product	aa	MW	PDB homolog	% identity
g144	85132-89052	3918	DNAP01	1306	152.71	1njZ; DNAP I *Geobacillus stearothermophilus*	12
g144 (3'-end)		1083	DNAP01-Ct	361	42.19		
g113	58885-59853	966	DNAP02	322	37.08	3zdd; protein XNI, Escherichia *coli* O157:H7	18
g158	99157-100053	894	DNAP03	298	34.32	3auo; DNA polymerase beta family, Thermus *termophilus*	12

Table S5. *LC-MS/MS analysis of the isolated DNAP01 protein before and after Anion Exchange Chromatography*

Before Anion Exchange Chromatography			
**Accession**	**Description**	**Score**	**# PSMs**
YerA41	DNAP01	4741,27	1305
A0A140N587	Bifunctional polymyxin resistance protein ArnA	1408,89	330
A0A140NH65	60 kDa chaperonin	857,30	234
A0A140NFV3	Chaperone protein DnaK	761,31	197
A0A140N7C7	Elongation factor G	309,15	80
A0A140NE25	Glutamine--fructose-6-phosphate aminotransferase [isomerizing]	285,86	73
A0A140NBA5	30S ribosomal protein S1	227,91	58
A0A140N6C1	DNA gyrase subunit B	218,64	56
A0A140N9B6	Succinylornithine transaminase	204,67	53
A0A140N3T4	Translation initiation factor IF-2	175,74	44
A0A140NDI2	Beta-galactosidase	151,59	43
A0A140NH27	DNA-directed RNA polymerase subunit beta'	144,99	43
A0A140NFZ9	Chaperone protein DnaJ	147,73	41
A0A140SS80	DNA-directed RNA polymerase subunit beta	150,76	40
A0A140N5F4	Polyribonucleotide nucleotidyltransferase	137,67	39
A0A140NE66	2-oxoglutarate dehydrogenase, E1 subunit	144,72	39
A0A140NFP9	Aconitate hydratase B	130,74	38
A0A140N6W0	Elongation factor Tu	124,55	37
A0A140N982	Bifunctional protein PutA	149,15	37
A0A140ND61	Chaperone protein HtpG	127,24	36
A0A140NC10	Citrate synthase	135,90	36
A0A140ND17	Protein translocase subunit SecA	120,99	34
A0A140NBG5	ATP-dependent Clp protease, ATP-binding subunit clpA	125,58	33
A0A140NEV5	Alkyl hydroperoxide reductase, F subunit	109,52	30
A0A140NBE7	Formate acetyltransferase	109,57	30
A0A140N6W9	Alanine--tRNA ligase	100,41	30
A0A140N6E5	D-tagatose-1,6-bisphosphate aldolase subunit GatZ	104,28	29
A0A140NCX5	DNA topoisomerase 1	100,12	28
A0A140N6Z9	30S ribosomal protein S5	91,66	27
A0A140NHS0	ATP synthase subunit beta	94,03	26
A0A140N775	Malate dehydrogenase	100,81	26
A0A140SS63	50S ribosomal protein L7/L12	62,23	26
A0A140N5L3	ATP-dependent zinc metalloprotease FtsH	79,06	25
A0A140NCE7	DNA-directed RNA polymerase	94,67	25
A0A140N5I2	Malate dehydrogenase (Oxaloacetate-decarboxylating) (NADP(+)), Phosphate acetyltransferase	91,41	25
A0A140NET2	Trigger factor	79,82	24
A0A140N8W2	Diguanylate phosphodiesterase	79,85	24
A0A140NF86	ATP-dependent protease ATPase subunit HslU	74,76	23
A0A140NBT9	Cell division protein FtsA	94,30	23
A0A140ND72	ATP synthase subunit alpha	85,50	22
A0A140N4G4	Glycine dehydrogenase (decarboxylating)	83,74	22
A0A140NAA1	Ribonuclease E	82,01	22
A0A140NE28	Glycoside hydrolase family 4	83,62	22
A0A140NCE4	Aldehyde-alcohol dehydrogenase	77,86	21
A0A140NBM4	Bifunctional uridylyltransferase/uridylyl-removing enzyme	79,01	21
A0A140NDE3	OmpA domain protein transmembrane region-containing protein	63,68	20
A0A140SS84	Acetylornithine deacetylase	71,33	20
A0A140N6A5	Chaperone protein ClpB	70,05	20
A0A140NGN0	Aspartate ammonia-lyase	65,87	20
A0A140N7U4	6-phosphogluconate dehydrogenase, decarboxylating	82,63	20
A0A140N3D6	Transcriptional regulator, Crp/Fnr family	53,33	19
A0A140N2U0	DNA-directed RNA polymerase subunit alpha	68,10	19
A0A140NBF4	Succinate--CoA ligase [ADP-forming] subunit beta	67,31	19
A0A140N7E6	Threonine--tRNA ligase	60,01	19
A0A140NAN3	Isocitrate dehydrogenase [NADP]	62,80	19
A0A140N2J1	33 kDa chaperonin	63,85	19
A0A140N6V1	Peptidyl-prolyl cis-trans isomerase	69,46	19
A0A140NC97	Peroxiredoxin	70,88	19
A0A140NA80	Succinate dehydrogenase flavoprotein subunit	55,64	19
A0A140NBR4	Chromosome partition protein MukB	55,96	19
A0A140N4Y7	ATP-dependent RNA helicase DeaD	72,67	18
A0A140NDL0	Pyruvate dehydrogenase E1 component	64,72	18
A0A140NF01	Transcription termination factor Rho	73,11	18
A0A140N2U8	Glycine--tRNA ligase beta subunit	53,91	17
A0A140N3H4	50S ribosomal protein L14	48,21	17
A0A140N582	Cell shape determining protein, MreB/Mrl family	57,60	17
A0A140NFM6	Cell division protein FtsZ	61,93	17
A0A140NFL6	Type II secretion system protein E	69,73	17
A0A140NFK0	Methionine synthase	55,35	17

After Anion Exchange Chromatography			
**Accession**	**Description**	**Score**	**# PSMs**
YerA41	DNAP01	4825,60	1432
A0A140N587	Bifunctional polymyxin resistance protein ArnA; MW 74.2 kDa	560,59	151
A0A140NFV3	Chaperone protein DnaK; MW 69.1 kDa	254,13	75
A0A140NE25	Glutamine--fructose-6-phosphate aminotransferase [isomerizing]; MW 66.9 kDa	237,48	63
A0A140ND61	Chaperone protein HtpG	78,03	28
P00761	Trypsin	63,72	16
A0A140N6V1	Peptidyl-prolyl cis-trans isomerase	62,57	17
A0A140NDL0	Pyruvate dehydrogenase E1 component	52,82	18
A0A140NET2	Trigger factor	47,94	15
A0A140SS61	Regulator of sigma D	45,01	13
A0A140NE13	Ferric uptake regulation protein	41,82	10
A0A140N6C1	DNA gyrase subunit B	39,19	14
A0A140N7C7	Elongation factor G	38,03	11
A0A140N562	L-aspartate oxidase	37,05	14
A0A140N6W0	Elongation factor Tu	30,48	10
A0A140N899	Glucose-6-phosphate 1-dehydrogenase	28,71	10
A0A140N7Y4	Pseudouridine synthase	26,88	8
A0A140NC10	Citrate synthase	24,27	7
A0A140NBA5	30S ribosomal protein S1	23,18	10
A0A140N4G4	Glycine dehydrogenase (decarboxylating)	22,58	6
A0A140NE43	tRNA-2-methylthio-N(6)-dimethylallyladenosine synthase	18,75	6
A0A140N340	50S ribosomal protein L27	17,96	7
A0A140N6V2	Diaminopimelate decarboxylase	17,73	5
A0A140N5A3	50S ribosomal protein L5	17,59	6
A0A140NBX4	Ribonuclease T	15,99	4
A0A140N4H3	30S ribosomal protein S12	15,38	5
A0A140SS84	Acetylornithine deacetylase	15,34	4
A0A140NFZ9	Chaperone protein DnaJ	15,11	7
A0A140NAC4	PhoH family protein	14,49	4
A0A140N9F9	tRNA (guanine-N(1)-)-methyltransferase	14,33	6
A0A140NAY3	Histidine biosynthesis bifunctional protein HisB	13,63	6
A0A140N9E5	Glutamate--tRNA ligase	13,39	4
A0A140NHQ8	ATP-dependent protease subunit HslV	11,41	4
A0A140N3L9	50S ribosomal protein L28	10,54	4
A0A140N9D5	Cysteine desulfurase IscS	9,91	4
A0A140N8B4	30S ribosomal protein S21	9,89	4

A0A140N6C6	Elongation factor 4	48,79	16
A0A140N745	NADH-quinone oxidoreductase subunit C/D	50,24	16
A0A140N753	Alpha-1,4 glucan phosphorylase	63,22	16
A0A140NFK2	30S ribosomal protein S2	50,38	16
A0A140N821	Fructose-bisphosphate aldolase, class II	59,27	16
A0A140NE27	Acetyltransferase component of pyruvate dehydrogenase complex	53,71	16
A0A140N8N4	Phenylalanine--tRNA ligase beta subunit	49,37	16
A0A140NAC4	PhoH family protein	60,71	16
A0A140NAC7	UDP-glucose 6-dehydrogenase	55,76	16
A0A140N783	Glyceraldehyde-3-phosphate dehydrogenase	53,06	15
A0A140NBL4	Outer membrane protein assembly factor BamA	47,87	15
A0A140N8S5	Protein GrpE	54,74	15
A0A140N7D6	Transcription termination/antitermination protein NusA	59,78	15
A0A140NGF8	Tryptophanase	62,73	15
A0A140NF15	Isocitrate lyase	56,24	15
A0A140NAN1	Quinone-dependent D-lactate dehydrogenase	52,95	14
A0A140ND68	Aminotransferase	44,79	14
A0A140NHC7	GTP-binding protein TypA	45,70	14
A0A140N6F6	Oligopeptidase A	55,34	14
A0A140NBS3	Dihydrolipoyl dehydrogenase	48,51	14
A0A140NDV9	Transaldolase	49,65	14
A0A140NDT0	Aminopeptidase N	56,03	14
A0A140N626	DNA gyrase subunit A	47,02	14
A0A140NDQ6	3-ketoacyl-CoA thiolase	49,14	14
A0A140ND70	Efflux transporter, RND family, MFP subunit	58,06	13
A0A140SS67	Phosphoenolpyruvate carboxylase	38,94	13
A0A140NFD7	Elongation factor Ts	42,38	13
A0A140NCH9	DNA-binding protein	48,83	13
A0A140N4Y8	Protein RecA	49,44	13
A0A140N7J1	50S ribosomal protein L2	42,64	13
A0A140N4K1	30S ribosomal protein S3	50,27	13
A0A140N4G2	Protein-export protein SecB	54,54	13
A0A140N9G2	Succinate--CoA ligase [ADP-forming] subunit alpha	43,71	13
A0A140NGH2	ABC transporter related	45,57	13
A0A140NDB1	Aminoacyl-histidine dipeptidase	44,62	13
A0A140N7E7	Chorismate mutase	47,45	13
A0A140N8Y0	Fumarate hydratase class I	45,63	13
A0A140NB77	Phosphoenolpyruvate synthase	44,90	13
A0A140NAN5	Porin Gram-negative type	45,68	12
A0A140N5B4	30S ribosomal protein S13	40,71	12
A0A140NA84	Phosphate acetyltransferase	46,58	12
A0A140NGG2	Peptidyl-prolyl cis-trans isomerase	42,49	12
A0A140NBN8	Beta-lactamase	39,26	12
A0A140N9N5	Phosphoenolpyruvate-protein phosphotransferase	47,20	12
A0A140N9Q3	GMP synthase [glutamine-hydrolyzing]	42,65	12
A0A140NBM7	Leucine--tRNA ligase	39,50	12
A0A140ND98	Isoleucine--tRNA ligase	39,06	12
A0A140N7T0	Luciferase-like monooxygenase	34,17	12
A0A140NCJ7	Argininosuccinate lyase	40,58	11
A0A140NCJ4	D-amino acid dehydrogenase	40,10	11
A0A140NE43	tRNA-2-methylthio-N(6)-dimethylallyladenosine synthase	40,07	11
A0A140N8E1	Phosphoglycerate kinase	43,52	11
A0A140NEK1	Adenylosuccinate synthetase	41,22	11
A0A140NFM7	50S ribosomal protein L1	36,71	11
A0A140NCP3	Thioredoxin domain protein	45,42	11
A0A140NDX4	Dihydrolipoyllysine-residue succinyltransferase component of 2-oxoglutarate dehydrogenase complex	36,93	11
A0A140N5L9	Cysteine synthase	44,03	11
A0A140N201	PTS system, mannitol-specific IIC subunit	35,14	11
A0A140NDD7	Sulfatase	42,49	11
A0A140NBB3	Pyruvate kinase	43,22	11
A0A140NDY4	Guanosine-5'-triphosphate,3'-diphosphate pyrophosphatase	34,15	11
A0A140NDZ9	Succinate dehydrogenase iron-sulfur subunit	32,59	11
A0A140N9Z6	Aldehyde Dehydrogenase	39,73	11
A0A140NFX6	Membrane protein insertase YidC	38,96	11
A0A140NHM8	Soluble pyridine nucleotide transhydrogenase	45,51	11
A0A140N9N7	UDP-4-amino-4-deoxy-L-arabinose--oxoglutarate aminotransferase	30,00	10
A0A140N5A9	Fe/S biogenesis protein NfuA	35,45	10
A0A140NC11	Peptidoglycan-associated protein	31,33	10
A0A140N5I0	Peptidyl-prolyl cis-trans isomerase	36,70	10
A0A140N5P0	Ribosomal RNA small subunit methyltransferase I	36,12	10
A0A140N9V8	Pyruvate kinase	36,33	10
A0A140N9A4	PTS system, glucose subfamily, IIA subunit	37,15	10
A0A140NF95	Sulfatase	34,03	10
A0A140NB96	Transcriptional regulator, LacI family	34,71	10
A0A140N7T7	DegT/DnrJ/EryC1/StrS aminotransferase	38,12	10
A0A140N8D7	Phosphomethylpyrimidine kinase	40,86	10
A0A140NGV6	Valine--tRNA ligase	29,96	10
A0A140NGG3	Two component transcriptional regulator, winged helix family	31,82	9
A0A140NF24	Histone family protein DNA-binding protein	33,57	9

A0A140SS73	Glycerol kinase	28,94	9
A0A140NCU5	6,7-dimethyl-8-ribityllumazine synthase	33,32	9
A0A140N2T1	50S ribosomal protein L6	28,11	9
A0A140N683	RNA polymerase sigma factor RpoD	30,68	9
A0A140NCS9	Ribose-phosphate pyrophosphokinase	33,25	9
A0A140ND23	3-oxoacyl-(Acyl-carrier-protein) reductase	33,46	9
A0A140N7N3	Glutamate synthase (Ferredoxin)	26,10	9
P00761	Trypsin	26,37	8
A0A140N6G0	Enolase	31,28	8
A0A140NDW9	2,3,4,5-tetrahydropyridine-2,6-dicarboxylate N-succinyltransferase	30,51	8
A0A140NEQ0	ATP-dependent Clp protease ATP-binding subunit ClpX	37,68	8
A0A140NHQ8	ATP-dependent protease subunit HslV	33,00	8
A0A140N711	50S ribosomal protein L15	24,80	8
A0A140N5A3	50S ribosomal protein L5	28,14	8
A0A140N6W8	30S ribosomal protein S7	28,85	8
A0A140NDE0	Proline--tRNA ligase	24,95	8
A0A140NE54	UPF0250 protein YbeD	29,13	8
A0A140N319	RNase adapter protein RapZ	32,29	8
A0A140N810	Transketolase	27,74	8
A0A140NBZ3	Protein HflK	24,56	8
A0A140N899	Glucose-6-phosphate 1-dehydrogenase	26,50	8
A0A140NDN8	Transcriptional regulator, LacI family	34,78	8
A0A140N5N9	TrkA-N domain protein	31,14	8
A0A140N4S8	Outer membrane protein assembly factor BamC	25,41	8
A0A140NFB9	Formate dehydrogenase, alpha subunit	25,82	8
A0A140NCV3	Peptidylprolyl isomerase	26,90	8
A0A140N793	Chaperone protein HscA	35,50	8
A0A140NEX9	Replicative DNA helicase	22,88	7
A0A140N8G3	Aminomethyltransferase	20,35	7
A0A140N9R4	Translation initiation factor IF-3	25,35	7
A0A140N8V2	Site-determining protein	25,73	7
A0A140N7A5	Nucleoside diphosphate kinase	27,45	7
A0A140NHL8	Transcription termination/antitermination protein NusG	19,99	7
A0A140N4N4	Peptidase B	26,92	7
A0A140N6Y5	30S ribosomal protein S10	20,96	7
A0A140N4H3	30S ribosomal protein S12	23,67	7
A0A140N996	Aspartate--tRNA ligase	24,72	7
A0A140N6J6	Histidine--tRNA ligase	21,70	7
A0A140NEI9	Tol-Pal system protein TolB	32,73	7
A0A140NEL5	Triosephosphate isomerase	26,85	7
A0A140N7W3	UPF0265 protein YeeX	24,72	7
A0A140N8K1	Transcriptional regulator, LysR family	32,81	7
A0A140N8E8	(P)ppGpp synthetase I, SpoT/RelA	21,40	7
A0A140N8V5	Phospholipase D/Transphosphatidylase	27,43	7
A0A140N7A4	Alcohol dehydrogenase zinc-binding domain protein	27,59	7
A0A140NHU6	Formate dehydrogenase iron-sulfur subunit	25,65	7
A0A140N4S7	Glutathione S-transferase domain protein	22,10	7
A0A140NAV6	Periplasmic serine endoprotease DegP-like	31,41	7
C5W865	Sulfite reductase [NADPH] hemoprotein beta-component	24,78	7
A0A140NAY3	Histidine biosynthesis bifunctional protein HisB	21,73	7
A0A140N9F5	Outer membrane protein assembly factor BamB	25,28	7
A0A140NAX3	FAD-dependent pyridine nucleotide-disulphide oxidoreductase	20,58	7
A0A140SS89	Fatty acid oxidation complex subunit alpha	19,98	7
A0A140NCE9	Malate synthase	26,00	7
A0A140NAW9	Adenylate kinase	20,31	6
A0A140N9D9	2,3-bisphosphoglycerate-dependent phosphoglycerate mutase	20,66	6
A0A140N7J9	Biotin carboxylase	19,56	6
A0A140NI98	ATP synthase subunit b	19,42	6
A0A140NAW2	Cold-shock DNA-binding domain protein	19,89	6
A0A140N942	GTPase Der	21,08	6
A0A140N2D9	Chromosomal replication initiator protein DnaA	24,13	6
A0A140NA92	3-oxoacyl-[acyl-carrier-protein] synthase 2	16,42	6
A0A140NCM4	Fatty acid metabolism regulator protein	25,56	6
A0A140N644	Glutamate--cysteine ligase	19,76	6
A0A140NCU2	Acyl-[acyl-carrier-protein]--UDP-N-acetylglucosamine O-acyltransferase	19,47	6
A0A140NHG8	Maltose/maltodextrin import ATP-binding protein MalK	22,99	6
A0A140N627	S-adenosylmethionine synthase	22,62	6
A0A140NDV1	50S ribosomal protein L9	19,59	6
A0A140NCR0	Ribonuclease R	16,90	6
A0A140N731	Cytoskeleton protein RodZ	22,11	6
A0A140N548	30S ribosomal protein S4	21,86	6
A0A140N536	Signal recognition particle protein	20,85	6
A0A140N1Y4	L-threonine 3-dehydrogenase	17,76	6
A0A140N9Z5	Cell division protein ZipA	20,25	6
A0A140N3M3	Protease TldD	25,68	6
A0A140NFA9	Histone family protein DNA-binding protein	21,14	6
A0A140NFY3	Carbamoyl-phosphate synthase large chain	24,19	6
A0A140NCD0	Maltodextrin-binding protein	22,54	6
A0A140NC81	Uncharacterized lipoprotein YdcL	17,28	6

A0A140N5W3	Aminotransferase class I and II	21,43	6
A0A140N4F2	Cold-shock DNA-binding domain protein	15,61	6
A0A140N7A3	ABC transporter related	26,91	6
A0A140N812	Transcriptional regulator, PadR-like family	22,25	6
A0A140NGG7	30S ribosomal protein S6	25,59	6
A0A140NBC5	Glutamate dehydrogenase	22,18	6
A0A140N870	Alcohol dehydrogenase GroES domain protein	18,31	6
A0A140N6Z3	Succinic semialdehyde dehydrogenase	22,14	6
A0A140N9S6	Diguanylate cyclase/phosphodiesterase with PAS/PAC sensor(S)	22,11	6
A0A140NEJ3	6,7-dihydropteridine reductase	16,92	6
A0A140N931	N-succinylglutamate 5-semialdehyde dehydrogenase	18,64	6
A0A140N5P6	Putative transferase	19,65	6
A0A140N6R8	Cell division protein DamX	20,64	6
A0A140ND59	Aspartate--ammonia ligase	22,51	6
A0A140N9P3	NADH-quinone oxidoreductase	18,51	6
A0A140NDE6	Acetyl-coenzyme A carboxylase carboxyl transferase subunit alpha	16,27	5
A0A140NCR5	Acyl carrier protein	15,32	5
A0A140NEW8	ATP-dependent Clp protease proteolytic subunit	17,09	5
A0A140N5E8	Phosphoglucosamine mutase	14,94	5
A0A140N9D5	Cysteine desulfurase IscS	14,63	5
A0A140NBC1	HTH-type transcriptional repressor PurR	15,20	5
A0A140NDB6	50S ribosomal protein L10	14,70	5
A0A140N6Z2	50S ribosomal protein L16	20,47	5
A0A140N5K8	50S ribosomal protein L4	19,41	5
A0A140N9P4	Dual-specificity RNA methyltransferase RlmN	17,28	5
A0A140NAU7	Ribosome-recycling factor	17,85	5
A0A140N7L9	30S ribosomal protein S11	15,62	5
A0A140N9E5	Glutamate--tRNA ligase	17,84	5
A0A140N8S8	Lysine--tRNA ligase	16,27	5
A0A140NAB7	Glutamine--tRNA ligase	17,30	5
A0A140NBF5	Serine--tRNA ligase	16,70	5
A0A140N751	Uracil phosphoribosyltransferase	18,15	5
A0A140NA83	Enoyl-[acyl-carrier-protein] reductase [NADH]	15,40	5
A0A140NDC7	Protein translocase subunit SecD	16,59	5
A0A140N4Y5	Aspartate-semialdehyde dehydrogenase	17,50	5
A0A140NGU5	Acetyl-coenzyme A synthetase	14,37	5
A0A140NDX8	GTPase HflX	19,80	5
A0A140NCU1	Malonyl CoA-acyl carrier protein transacylase	19,96	5
A0A140N9G9	Beta-ketoacyl synthase	11,66	5
A0A140NEQ9	Two component transcriptional regulator, winged helix family	20,78	5
A0A140SSA2	Glutamine synthetase	20,51	5
A0A140NBD3	Phospho-2-dehydro-3-deoxyheptonate aldolase	14,85	5
A0A140N4V4	Cell division ATP-binding protein FtsE	23,44	5
A0A140NE13	Ferric uptake regulation protein	17,00	5
A0A140NBC0	DNA repair protein RadA	19,52	5
A0A140NC45	ABC transporter related	19,29	5
A0A140NER9	DNA polymerase I	14,45	5
A0A140N8G9	Sulfite reductase [NADPH] flavoprotein alpha-component	15,23	5
A0A140N6K2	N-succinylarginine dihydrolase	18,84	5
A0A140N3P4	Glycosyl transferase family 2	15,68	5
A0A140NBF7	Catalase	13,52	5
A0A140N8Q5	Putative PTS IIA-like nitrogen-regulatory protein PtsN	17,98	5
A0A140N8C6	Methionine--tRNA ligase	12,86	5
A0A140N4C7	(P)ppGpp synthetase I, SpoT/RelA	15,22	5
A0A140NEY2	Maltoporin	16,58	4
A0A140SSC0	Thioredoxin	15,68	4
A0A140N3N8	N-acetylneuraminate lyase	18,34	4
A0A140N448	Heavy metal translocating P-type ATPase	14,70	4
A0A140NEV1	Probable cytosol aminopeptidase	9,00	4
A0A140NEG9	Acetylglutamate kinase	18,18	4
A0A140N7C3	Argininosuccinate synthase	11,15	4
A0A140NBM9	Arginine N-succinyltransferase	15,51	4
A0A140NF41	ATP synthase gamma chain	15,89	4
A0A140NEN6	10 kDa chaperonin	12,06	4
A0A140NA88	Cytochrome o ubiquinol oxidase, subunit I	12,66	4
A0A140N913	3-oxoacyl-[acyl-carrier-protein] synthase 3	18,02	4
A0A140N7B7	Inosine-5'-monophosphate dehydrogenase	17,30	4
A0A140N7J2	LPP repeat-containing protein	15,48	4
A0A140NFH4	Maltose operon periplasmic	16,40	4
A0A140N3F8	3,4-dihydroxy-2-butanone 4-phosphate synthase	14,88	4
A0A140N9Q4	Ribonucleoside-diphosphate reductase	16,47	4
A0A140N811	30S ribosomal protein S15	14,39	4
A0A140N9V2	Tryptophan synthase alpha chain	14,92	4
A0A140NDC1	UPF0227 protein YcfP	14,83	4
A0A140N9F7	Uncharacterized protein	9,53	4
A0A140NEK4	Modulator of FtsH protease HflC	16,61	4
A0A140N652	UPF0149 protein YgfB	19,48	4
A0A140N923	Iron-sulfur cluster assembly scaffold protein IscU	10,67	4
A0A140N4Z4	Transcriptional regulator, DeoR family	14,92	4
A0A140NB60	RNA polymerase-associated protein RapA	10,26	4
A0A140N914	Nitroreductase	13,07	4
A0A140ND82	Sodium/proline symporter	12,63	4

A0A140NBN1	Short-chain dehydrogenase/reductase SDR	16,29	4
A0A140N859	Transcriptional regulator, GntR family	7,69	4
A0A140NG09	Threonine synthase	12,19	4
A0A140N771	Stringent starvation protein B	12,64	4
A0A140N9H5	tRNA/rRNA methyltransferase (SpoU)	14,97	4
A0A140NAU1	NAD-dependent epimerase/dehydratase	11,22	4
A0A140NEF7	Phosphopentomutase	14,81	4
A0A140N9H6	Uncharacterized protein	11,98	4
A0A140N562	L-aspartate oxidase	11,65	4
A0A140NCG0	Thiol peroxidase	17,49	4
A0A140N7Y4	Pseudouridine synthase	13,56	4
A0A140NH75	Cell division protein ZapB	11,33	4
A0A140NF57	Transcriptional regulator, LysR family	11,93	4
A0A140NEX7	Periplasmic binding protein/LacI transcriptional regulator	15,78	4
A0A140N7Q7	Two component transcriptional regulator, winged helix family	8,23	4
A0A140NFX8	DNA-directed DNA polymerase	11,14	4
A0A140NGF2	Uncharacterized protein	12,21	4
A0A140N692	Uncharacterized protein	12,09	4
A0A140NC56	Purine nucleoside phosphorylase DeoD-type	12,87	4
A0A140NCK8	Aconitate hydratase	13,31	4
A0A140N9I7	3-ketoacyl-CoA thiolase	17,54	4
A0A140N9Z7	NADH-quinone oxidoreductase subunit F	13,26	4
A0A140N5G6	Twitching motility protein	12,15	4
A0A140NC67	Phosphoglucomutase, alpha-D-glucose phosphate-specific	12,26	4
A0A140N9E8	Endopeptidase La	10,21	4
A0A140NBF1	Glucans biosynthesis protein G	10,75	4
A0A140NAR5	Ribosome-binding ATPase YchF	10,94	4
A0A140NAB3	Diguanylate cyclase with PAS/PAC sensor	14,27	4
A0A140N6V3	NADH:flavin oxidoreductase/NADH oxidase	16,15	4
A0A140N9U3	FAD linked oxidase domain protein	13,56	4
A0A140N6I5	Selenide, water dikinase	12,58	4
A0A140N8E6	Protein-N(Pi)-phosphohistidine--sugar phosphotransferase	16,87	4
A0A140N4H6	Thiol:disulfide interchange protein	10,45	4
A0A140N4Y2	Peptidyl-prolyl cis-trans isomerase	16,73	4
A0A140NF66	Catalase-peroxidase	13,85	4
A0A140NCP4	Transcription-repair-coupling factor	10,27	4

ArnA - Bifunctional polymyxin resistance protein ArnA - Escherichia coli (strain B / BL21-DE3) https://www.uniprot.org/uniprot/A0A140N587. Accessed September 30, 2019.
DnaK - Chaperone protein DnaK - Escherichia coli (strain B / BL21-DE3) https://www.uniprot.org/uniprot/A0A140NFV3. Accessed September 30, 2019

Table S6. *Sigma-70 promoters predicted by the BPROM tool from the sequences upstream of each YerA41 gene. The linear discriminant function (LDF) threshold for the search was 0.20. The predicted -35 and -10 boxes are highlighted in green and the promoters with higher LDF are shown highlighted with stronger shade of brown.*

Prom	The next gene	Sequence upstream of the start codon of the gene	LDF	-35 box score	-10 box score
P1	g001	CATAAACGTTTATAAACGTTATTGGAGCTTATTTATTCTCAATTAATGAACTTGATAAGAG.. -P2-	3.40	39	57
P2	g001	TTTATTACCTTTACTGTTAACTTAGAACTTATTTAGCACATCAGGCCTTTATCAATAACTGCTGATTTGCGAGGATCAATTCTCC	0.83	42	8
P3	g004	AATGAGTATCATTGCAGTATTAGCATTAGCTGCTATCATCACATCAGTACTTAAACTTTAAGGAATTATA	5.34	49	70
P4	g017	AGCTGGATTTATTGATTACTATGATCTGCATGATATATTAGGATTTAAATT	3.10	53	62
P5	g021	TGGATGTTATGATGAAAAGAAAGATCCTAATCTTAAAATAAAAGGATCATCAAA	6.69	30	72
P6	g027	CGTGGTGGATACATTAAAATATCTGATGAATGCTATAATAATTAAATAGGATCTACT	8.55	37	88
P7	g041	ATCCATAATAATTCAATTAGGATCCTACAATTGTAGGATCCTTTTTATTTTGGAGGAATTAATCTT	9.17	35	54
P8	g046	GTAGAAGAATTCAGTGTTAAGGATAATCTTCGTTATACTCAATTACAACTTGAATATGATCGCATCAAAAATATTCGATAAGGATAAATA	4.89	18	79
P9	g054	ACGAATGGACCATCAAACGAGTACTCCATATGGTAAAATGGAAATACTAGTAGGATAAATA	6.42	10	87
P10	g057	GTCATGAGGTTCTCAGAACAATAACTGCCATTAGAGAATCTCATGACTCA	0.48	33	22
P11	g061	CTCAGGTAATTCTATAATATTACTGCATAGTGGTATTATAGAATTACCTTATAGGAATATTGCGT	4.52	22	80
P12	g065	AAACTGGATGACTCCATATGGAGTCATCCTTTTTATTTTAGAGGTTATT	6.22	31	52
P13	g073	GTGATGAATGGTGAAGATGTAATTCTAGGATGTTTTTGTTACCCTAGAAGATGTCATTGTGATACTATTGTTAAATACGTGCTTAAATTGGTAGGATAAA	2.37	25	48
P14	g076	ATTCTCCCATTCATTAATGATAATTCAATGAGTTATCATTAATGAATTCACTCAACTAGATAATCATCATAAGGATGAATAAA	6.52	33	70
P15	g079	TGAATCAATTGAGAAATATTGATTCATCGTATTTATAATTTCATAAATACGATAACTAGAAATTAGATAGGTAGGTATAAT	8.00	43	81
P16	g087	GAACTGCACTCTTTCAAGTGAAAGATCGTGATCTATACTGATTATTTTGATTTAATATAAGGAATTAG	5.08	36	66
P17	g091	TCGTTAATCGATAAATAAACTTAATAAATAATCTATCATTAGATTAAAGGATAAGTAA	11.17	21	58
P18	g096	TCAAGATAACTGTAACGTAGCAGTCACTACTGGTGAACTTGTTGACGATCGTAATAAATAATTAGTGAGGACATT	7.17	12	39
P19	g106	TTTAATTCTGTTGATGAAGTAGTAGTAATTCTCCAGAATTACTATAATGATCCAGCATCTGTAGTGTCTGATCAAATCGCTCACTAAGGATTGTT	5.25	52	34
P20	g109	GTAGTAGTCAACGAATCATCTCTCAGATCTTTGTATTATATCCTGGATCATCA	2.62	0	73
P21	g112	GCATAGATTCTATTGAAAAATTATTATTTTAGCTAAAATAAAGTAGGTAAATC	5.45	60	76
P22	g120	AATGGTAATGTGATTGGTAGTGTGAAGGATGGTAAAGTAACACTAGAAGGTAATAATGTTTCAATACTATCATCTTAGAAGATAGTTGATGAGGTGATAT	4.12	20	63
P23	g123	TTATAAAAATCTTTATTCCATTACTTTATAATCTATAATTTATGCTTAATATAAATAATATGATTTTGAGGTAATA	10.32	34	76
P24	g127	TATCAGATAATTAATCAATGAAGTAGTTCATTGTATAATTAATTCCTCAAGGATCAATGT	6.13	13	87
P25	g131	TTTAATAGATCATCTACTATACTTAAATATTTCGATAATTTTGATGATTTTTATAATTTTGTTACGAGGTAATG	9.78	5	45
P26	g140	TGAATTTAAGAAAGTTCTCCGTGGCATGGGTATTAAGATCGAGGATAATAAATAA	5.57	20	56
P27	g146	TCAATTAAAAATGACTCAGGGAGCTTGGTAGGTTATAATTTATCATTGTCATGGCATCCTAAAGAAATTGATTATCTATTAAGTAAGGTAACTGAAAA	8.06	31	90

P28	g150	ATCAATATCAATTAATTCGTAAGAAAATTGATCTATAATTGAATATCAGTTCAGTTATCGAGGATTT	4.82	30	76
P29	g157	GATCCATTGTAGATTAATTTATTCGAATAGGAGTAGAATA	7.28	41	60
P30	g162c	GATCAATACTTTATAAAGATAATAAATAAAGGATAAGAT	6.57	39	56
P31	g168c	AGTTACATTAAAATGCGTCCTCGTAACGTAGCTTATAATTATATAGTTCGTATGGCATAAGGTGTAATAAA	8.92	37	76
P32	g177c	AAGGTAGTAGGACTTGATTCAGAAATTAATATATACAATACTGAATTATCACCAAAAGGGTAGAATAA	9.48	53	54
P33	g182c	ATTCGGTCATAGAATGAGGCTGAATAGAGATTATACAATGTAAGGATTATC	3.53	18	59
P34	g186c	CTAATACATTTTACAAAACCATCGTCAATGAATTAGAATTGTACGACTCAATGAAAGCTAATAGTTAATTAATAGGAGATCATTT	3.80	47	57
P35	g189c	TCTTCAATAGTTCTGTTGATATCTCAGTATCATTATAATTGAGGATAAGTTAAC	6.08	5	75
P36	g195c	TATTCATAAATTTAAATTTATTAGATTAAATAATAAATTTGATTTGGATAATCTATTTAATGGAGCATGTC	8.47	41	41
P37	g199c	TCACACTGGTCTAATTATACTTTGCATTAATTGTATAATTAACTAATAATGGTACCACTCATATGAGTGGTACCATTTTCTAGTAACAGTGGGATTCTTC	9.15	11	87
P38	g202c	ATAGTGTTACTTAGGGAGCTCCCTAAAGATTCCTATAATTATAATTCTATGAGGTCATCT	5.30	25	74
P39	g206c	TCATCAGAGACTTATGGAAGAAGATGAGATGGCTATAATCAATGGATTAATGTAATTTTTATGGGATTCAAT	6.99	2	85
P40	g209c	GATCAGCAATTCCCATTCTATGTCACCATTGAGCAGAATGATTAAGGAATTCAA	1.71	41	34
P41	g213c	TAATTATATAATTATAATTAATCATGAATATACTATAATAAATAGTTAATTATTAAATTAATTTAATCTTGATGAATTACTTGAACATAAGTAATGATTGAACTGGAGTAAATC	15.16	24	74

Supplementary Figures

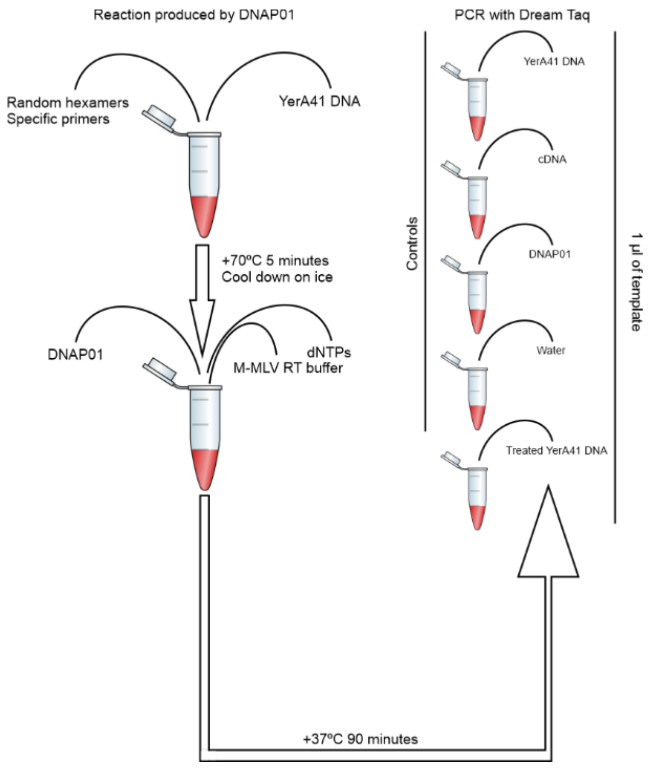

Figure S1. *The DNAP01 functional assay. On the left, the set-up of the complete reaction mixture in the test tube starting with heat treatment to allow random hexamer or specific primer annealing. After cooling down, reverse transcriptase buffer, dNTPs and purified DNAP01 were added to a total volume of 15 µL, and the reaction was incubated at 37 °C up to 90 min. One µL of the reaction mixture was then used as a template in a conventional PCR with the Dream Taq DNA polymerase and YerA41-specific primer pair.*

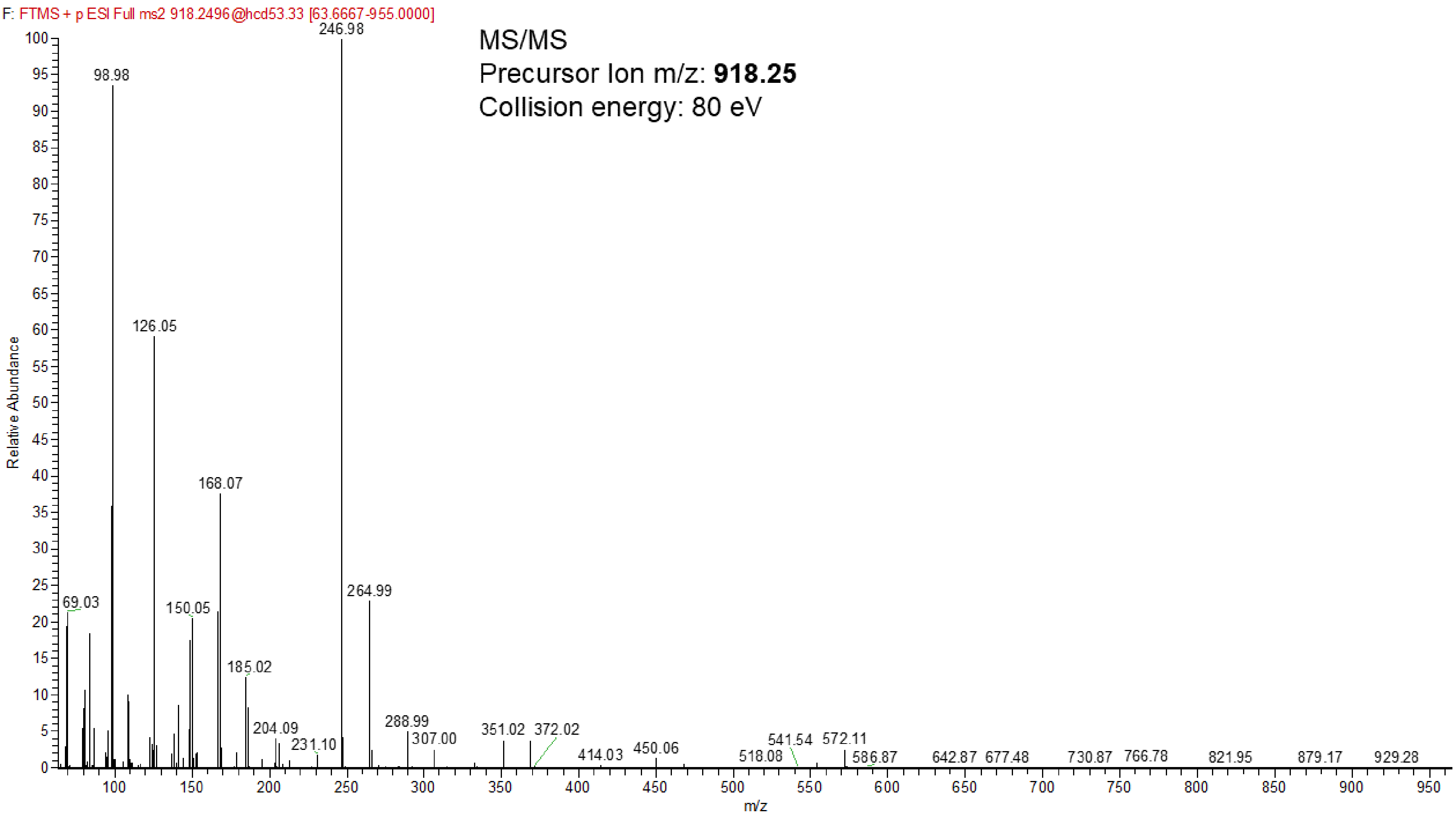

Figure S2. *Tandem mass spectra of precursor ion m/z 918 fragmented with 80 eV collision energy.*

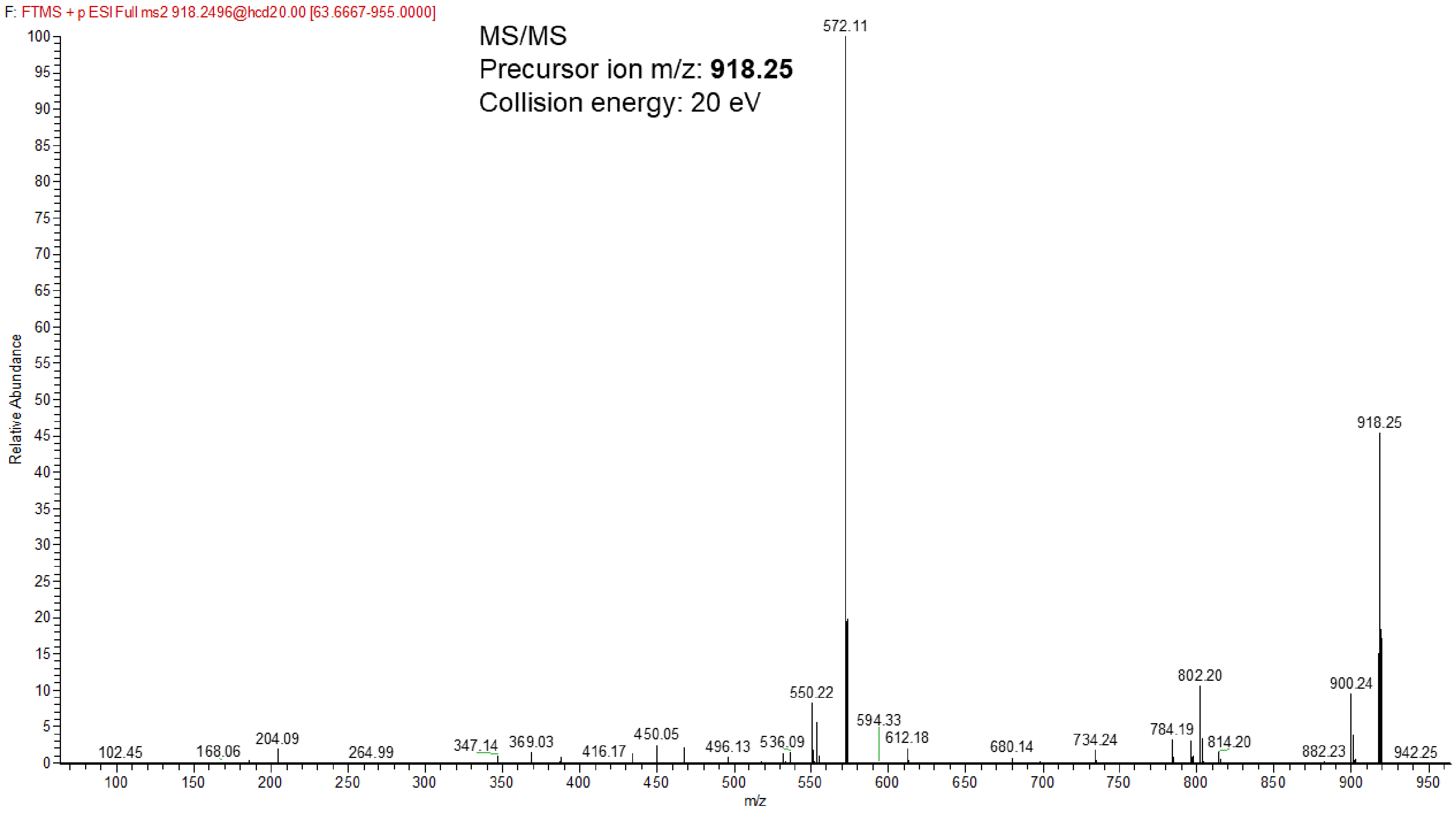

Figure S3. *Tandem mass spectra of precursor ion m/z 918 fragmented with 20 eV collision energy.*

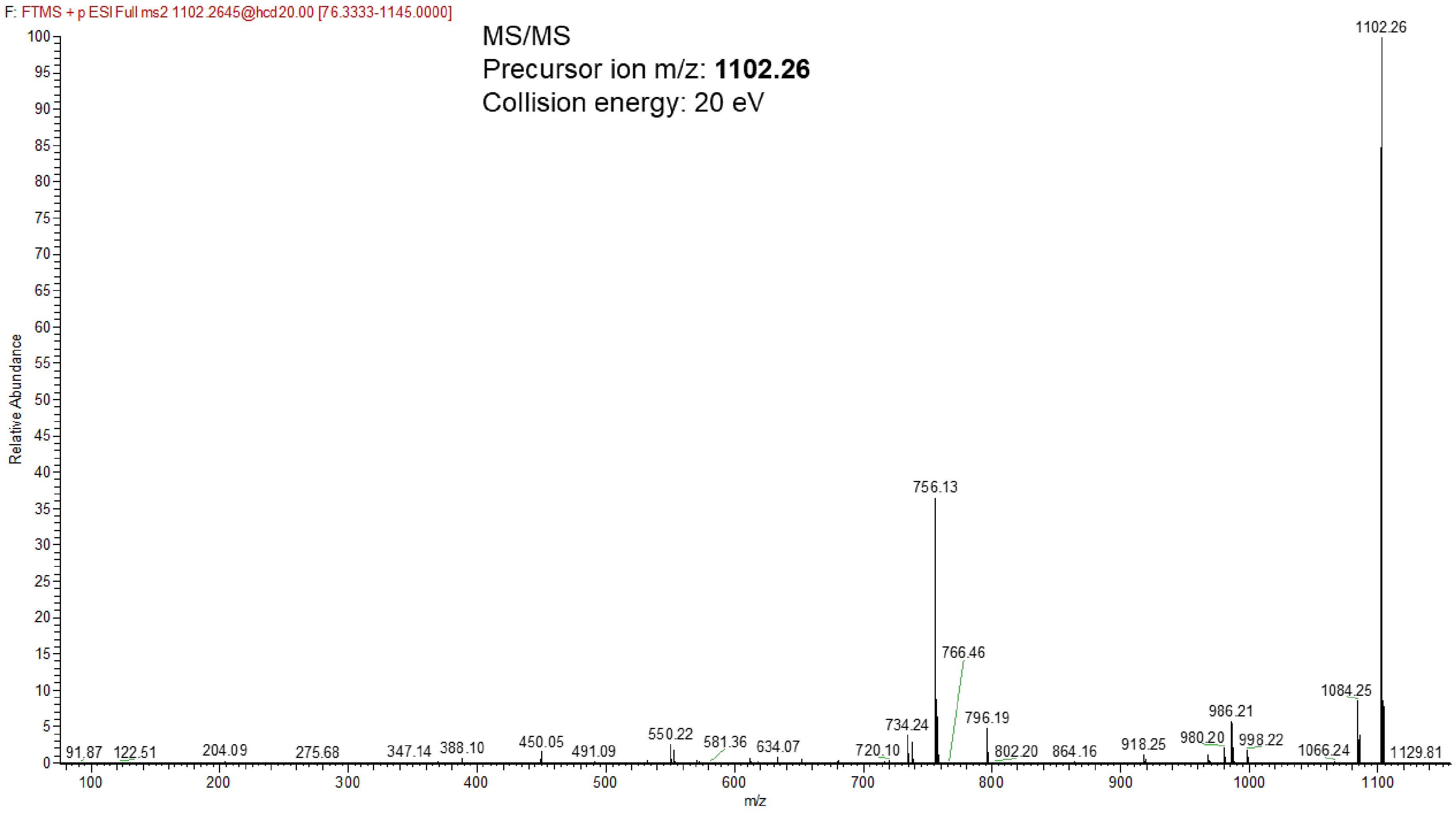

Figure S4. *Tandem mass spectra of precursor ion m/z 1102 fragmented with 20 eV collision energy.*

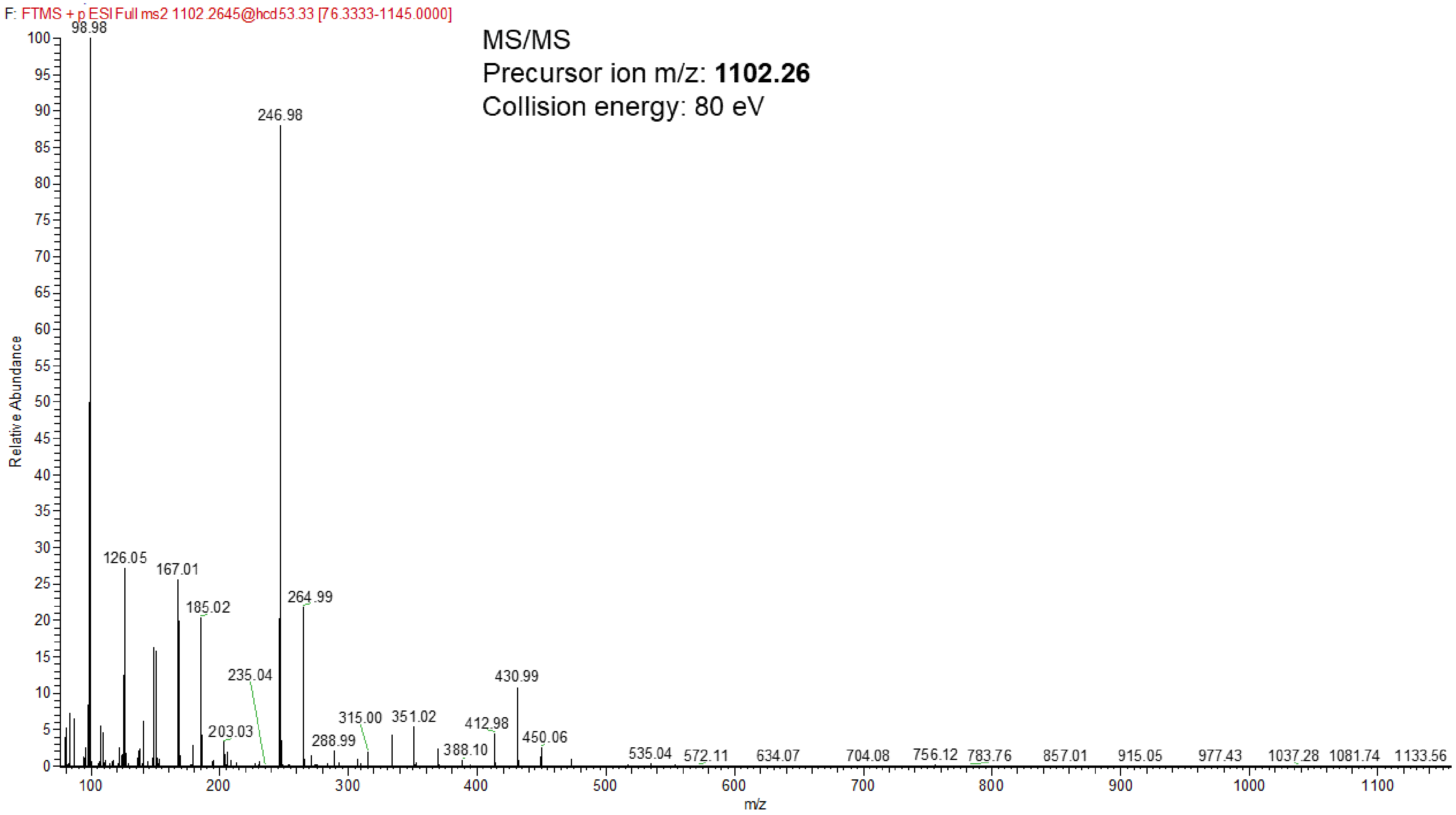

Figure S5. *Tandem mass spectra of precursor ion m/z 1102 fragmented with 80 eV collision energy.*

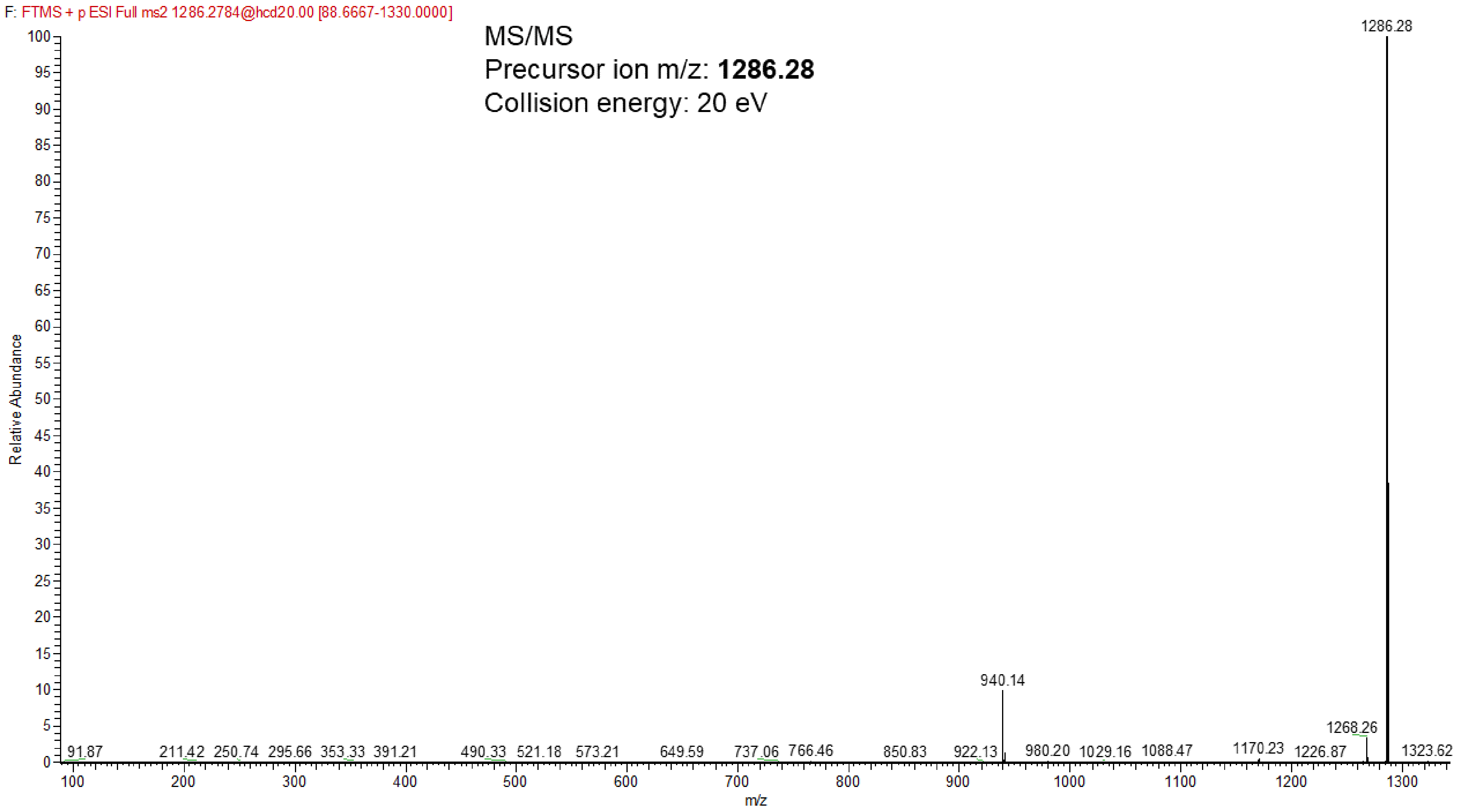

Figure S6. *Tandem mass spectra of precursor ion m/z 1286 fragmented with 20 eV collision energy.*

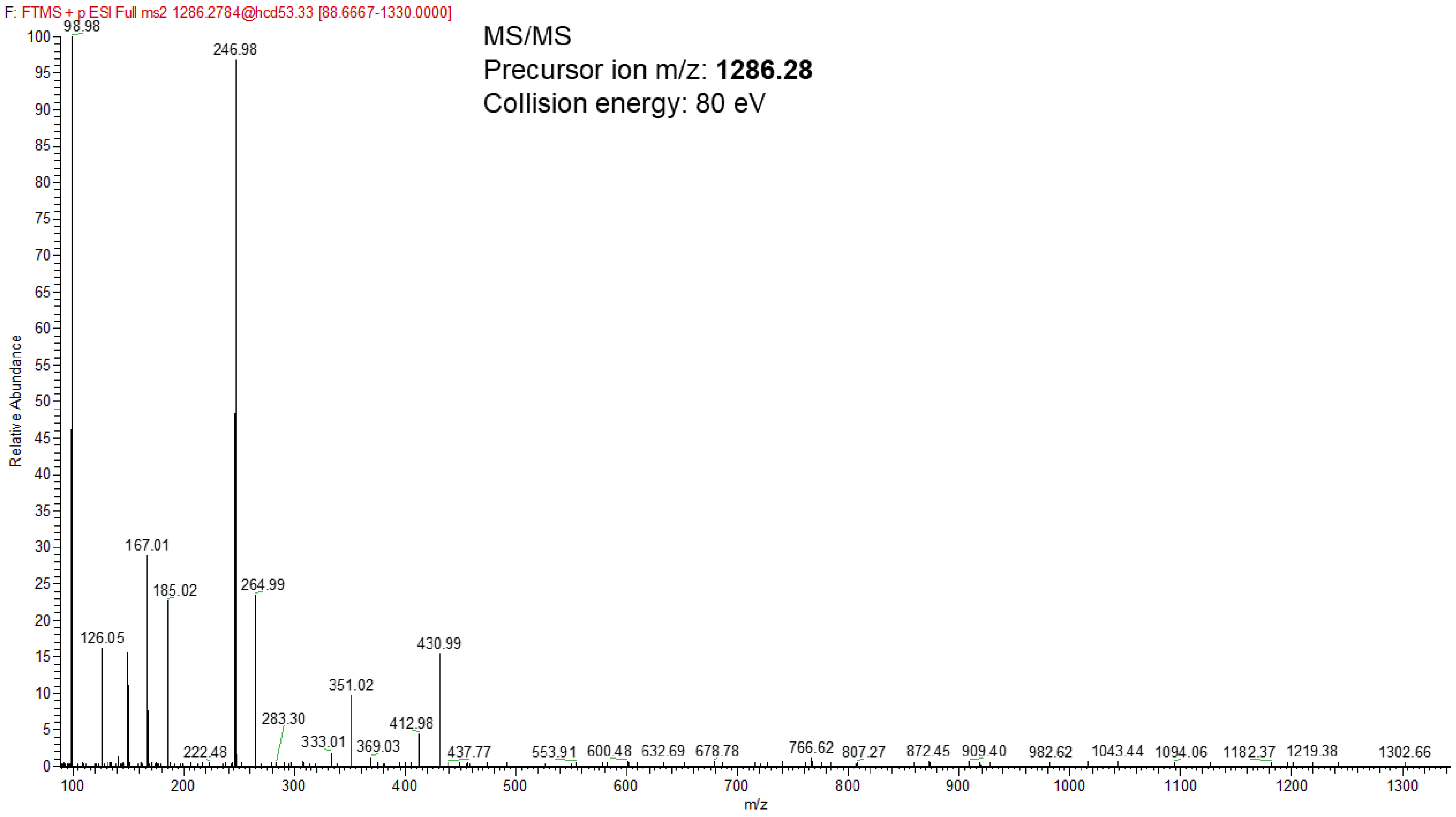

Figure S7. *Tandem mass spectra of precursor ion m/z 1286 fragmented with 80 eV collision energy.*

```
                772
YerA41_DNAP01   PAGHEMVILQFLLVNRLKSTSIDHEFIDKMPSDEFGEYLVDLVKRGNLAEAGQGKRLSAADKIILLEVKT-AKGTLSKILSGEKLGKHALRTIKQAWDFKNGRKNSLDEYPDFIQLIYHL
RHph_TM39_DNAP  ----------------MSVDWTKYDGVDLKTAKSIQKLMMDLI--GTLDEKIGWKLYAFRSDVLEWQ------------YKAHETYGGLDIDNRETWT------------PEF-ELLYLL
RHph_Y65_DNAP   LVDRQDL--NKAIENLMSVDWTKYDGVDLKTAKSIQKLMMDLI--GTLDEKIGWKLYAFRSDVLEWQ------------YKAHETYGGLDIDNRETWT------------PEF-ELLYLL
Flavob_DNAP     QKEFPTFFPLYKKIISLESTEDKINFLEEVLKNADEFLKKPFV--ENMKANSRTKEVELKAAQRNWEIPSLSQEYTSKMYSIFSDIFGIDPNDDSNWP------------EEF-FALYYF
RP13_DNAP       LGDKSFIYQNKPAVVMEAIL--GVTQQNNALGKGVQQSLNFAV--NTLPENI-RR---FAGPVVKFQ------------YKMHNTFLKLNPDDESTWS------------KEF-RMLFNI
IME392_DNAP     L-EKEFFREFPVDEILTRIIQFGDTAESKHVRELIAMCVQDGL--EAYEERFTNK---FKADVLDAQ------------LAIHQLMLGVNPDDESTWN------------TSF-RMMVDL
Consensus                                                                                                       *               *

                891                                                                                             PolA Motif A
YerA41_DNAP01   QLLKRHNKSISTVIEGSLGGGSFIVSHKDSHI---PIGPKTS-WKDIENLPEDT-------QIFYKTTYWQNDKDTLRWSSSFHTIPSTLDYSLCFTPRKEGRMFFHMDMAQAEVRIAFA
RHph_TM39_DNAP  RRYKKVEKSKNTYINGKVGRARVWLSTVDDYQKP-PVRLKRY-FDVMEERQWKKYELQHNERWILDTEFNVAGANTKRWKAAIHTVPWGSELREIYASRWWDGLFMHYDYSQAEVRILAA
RHph_Y65_DNAP   RRYKKVEKSKNTYINGKVGRARVWLSTVDDYQKP-PVRLKRY-FDVMEERQWKKYELQHNERWILDTEFNVAGANTKRWKAAIHTVPWGSELREIYASRWWDGLFMHYDYSQAEVRILAA
Flavob_DNAP     KVFKKVEKSIGTYLNGSLGRSSVEIVNYSDLEKECPPRIAQY-----SDRPKNE-----NEVYINRTGWGVCTAGTKRWQAGQHTVPKSTELMDLRCSRYKDGIKLHYDYSQAEVRVLAI
RP13_DNAP       FYYKKLNKMVSTNVNGTTGRSACHQIVGTLHG-K-PLRGANY-WDLVEQKVD-----MSSVQLILNTDFNTLSAATTRWSAGFHTVPASSPARKCFTV-PEDEIWVHADYSQAELVLLAF
IME392_DNAP     KMYKRHYKQIATYIDGKVGRQTVWAAKLRDGL-P-PLRVRKYDWD--NPKLQ-----DGEDCYIMTPDFNSLAAITSRWQAGIHNVPGDSTLRNIWVSKPEGGLWYHQDFSQAELVMVSV
Consensus          *   *   *   *  *                *                                       * **    *  *                   * *  ***

                1000                                           ---PolA Motif B--
YerA41_DNAP01   VAKEMGLINAILK-GLDIHGFNANNAFDLGFAEDELYKIKQDPELDQLRSYAKMLTFAILYGASVGSIA-KQIKKPFDEAKKIYDGYFNANPNFKKFVEDNIAELSEN------FGFRYL
RHph_TM39_DNAP  ISQDRALLEAFADPDTDIHMFVASRIW----------KKDPKDVTPAERRYAKVCSFSILYGKSIEGFATEHTKGDVAAAKRIFDDFYAAFPQVKEWIEKQHRDAKVSGKVFTLFGDPLY
RHph_Y65_DNAP   ISQDRALLEAFADPDTDIHMFVASRIW----------KKDPKDVTPAERRYAKVCSFSILYGKSIEGFATEHTKGDVAAAKRIFDDFYAAFPQVKEWIEKQHRDAKVSGKVFTLFGDPLY
Flavob_DNAP     MSGDENLLQAFKD-GLDIHLYNAARMW----------KKDPKDVTSTERSMAKGVTFSLLYGASVSEFANKFTNGNYDAAKKIINSFFASYPKVEEFIKESHRLGTFTGTVPTVFGDPLY
RP13_DNAP       LSGDPTMIQAFLD-GKDMHQFMASKVF----------EVDYDQVSKDQRKYTKTINFGIVYGKSVENIAIEITGGDVAKAQNLFDTIFRTFPGVEIWMNEKKKEVDDFGYVTTLFGNRLL
IME392_DNAP     FADDSDMKNVFSS-GGDMHRFVSSIAY----------QKSEPEVTDVERKGGKAINFALVYQSSLESVAMVATGGDMERAQNLMDTVFGKFTGLKAWIDATKKNGFETGYAYGYFGNR--
Consensus                        * *                             *   *   *   *  *    *          *                                  **

                1112                                                                                  PolA Motif C
YerA41_DNAP01   PIFNHRFY----------IGNPYHYSIK----------------------QKGLNYIIQNLSSSLTAYTAYALYDDLRTNYGVEIQLLGFVHDAIEFEFDAKDLFIILDRMNYWYKEMPL
RHph_TM39_DNAP  LDMSD----------------------------------------P-NGAMRDAQNWPIQSSSSCVAGWAIWMNYEFSRNR-NIPILPMVFTHDSHDMEFRCQHLFETIDTVLETAVDLPK
RHph_Y65_DNAP   LDMSD----------------------------------------P-NGAMRDAQNWPIQSSSSCVAGWAIWMNYEFSRNR-NIPILPMVFTHDSHDMEFRCQHLFETIDTVLETAVDLPK
Flavob_DNAP     VDMPDWVFSLDDETKLALIENPYNPSIKIINKKTDDQEERKQRAKYSKALRNCQNYRIQSSSSTLAGLGMEELQSKIEEF-NLSTKLECFTHDSCDADLRIKDLIRTMELVKSTSVDYLL
RP13_DNAP       IDVNE----------------------------------------PGNGRYRKGVNAPLQGGASTIAGTSIESFSSSCDKD-GIPEHSMGFTHDAMDSASKIDYVFPYIDLMVQRLQTDLR
IME392_DNAP     --INL----------------------------------------EGTNVNSTSVNYPIQNTSSMVAGAGMYFLDEDFKQS-NMEAETHIMVHDSLDTTSGINEMFKVFQKTRENMEGAIR
Consensus                                                             *   *   *                            **

                1200
YerA41_DNAP01   EKWDIPSDFDFELGSSRYSGGSCK-VKYNDDKSMADIKLEIKDYYND-KEDILKLMKESFNIIEDSLKKEDHPVEKHPFVFMIDGVKPD-CRFREEVTD-----QYVYSAKIGLK--
RHph_TM39_DNAP  RRFNLPMKIDWEIGVNQSDAIEFKETSRFDEGRGRTYHFECNENA---MMPVFDRLKNYFDIEYEVTSRETERASLKEMFVARRAFSKY---LGTDVTV--VGGNLKLVKKNGIKI-
RHph_Y65_DNAP   RRFNLPMKIDWEIGVNQSDAIEFKETSRFDEGRGRTYHFECNENA---MMPVFDRLKNYFDIEYEVTSRETERASLKEMFVARRAFSKY---LGTDVTV--VGGNLKLVKKNGIKI-
Flavob_DNAP     EQYNIPMKIDMGIGVSNNKIVELSDININD--NVIKAEFEGTQESLTLLKNKIE--NNNGKLEYSIEESKESIISIKDLFLTTNAYAQS---MGKPFTN--LKGELKIAI-------
RP13_DNAP       EKMGIPMSIDYELGANAYNICHWHE--INQEGDVKTIHIEGENESTELLIKKLDKSTTYKVLSSEVIKSKSEATSWAEMFTTGTALKDS---WGKTVTKNTVELKVQMLPQTCM---
IME392_DNAP     ELWDMPMRTDIEFGARGGSLMGLDDYGVNEDGSGWLI-VEGRESALEKLLEALDQYSQWTPYV-EILKEKEKLNSIANSFNSKSGIRSDGTGWGCKYIE--LKAKITLEPKHGEKAA
Consensus            *   *   *                         *
```

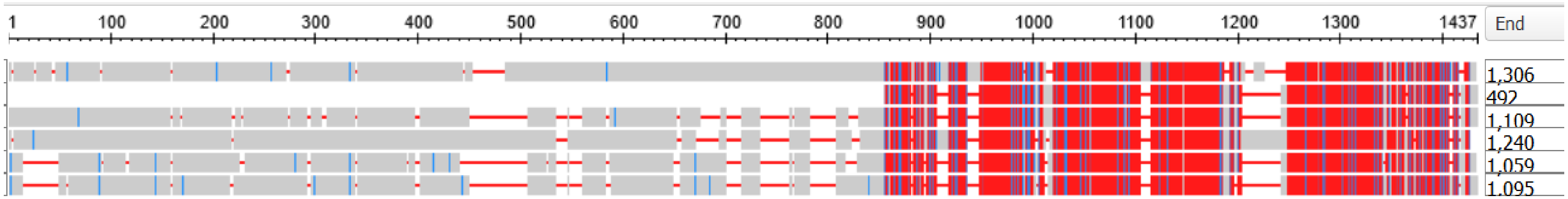

Figure S8. *Multiple alignment of* YerA41 DNAP01 *with its BLASTp-identified closest related sequences identified as follows:* Hph_TM39_DNAP, *DNA polymerase A domain-containing protein of Rhizobium phage RHph_TM39 (Acc.no QIG77270.1);* RHph_Y65_DNAP, *DNA polymerase A domain-containing protein of Rhizobium phage RHph_Y65 (Acc.no QIG72859.1);* Flavob_DNAP, *hypothetical protein of Flavobacteriaceae bacterium (Acc.no NNK81911.1);* RP13_DNAP, *DNA polymerase I of Ralstonia phage RP13 (Acc.no BCG50106.1);* IME392_DNAP, *putative DNA polymerase I of Escherichia phage vB_EcoM_IME392 (Acc.no AXY86073.1).*

TOP PANEL. *Multiple alignment of DNAP01 residues 772-1306 with the others. Identical residues with that of DNAP01 are highlighted in grey, and residues shared by all sequences are indicated by asterisks on the consensus line. The DNA polymerase PolA motifs A, B and C are indicated above the alignment.*

BOTTOM PANEL. *Overview of the alignment over the full lengths of the proteins (indicated at right). The coloring of the amino acid residues highlights highly conserved and less conserved amino acid positions based on the relative entropy threshold of the residue. Only alignment positions with no gaps are colored. Red indicates highly conserved positions and blue indicates lower conservation. The alignment was carried out at https://blast.ncbi.nlm.nih.gov/Blast.cgi (accessed on April 4, 2021).*

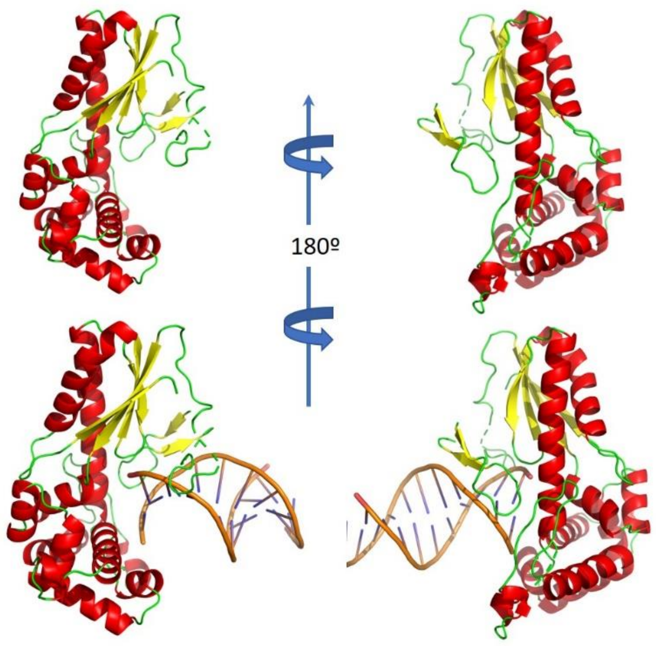


```
                                                     Motif A
DNAP01   946 TTYWQNDKDTLRWSSSFH---TIPSTLDY----SLCFTPRKEGRMFFHMDMAQAEVRIAFAVAKEMGLINAILKGL  1014
             +++-|....|-||||+--   .||+.-.+    --||.|+..+..+.-+|..|-|+||.-..++.-.|+.+...|.
 1njzA   308 TIFNQALTQTGRLSSTEPNLQNIPIRLEEGRKIRQAFVPSESDWLIFAADYSQIELRVLAHIAEDDNLMEAFRRDL  383

                                            Motif B
DNAP01  1015 DIHGFNANNAFDLGFAEDELYKIKQDPELDQLRSYAKMLTFAILYGASVGSIAKQIKKPFDEAKKIYDGYFNANPN 1090
             |+|-..|-..|  |...++.++        ..|..||.+.|+++||++..++|+++.-+-+||+.+.+.||+..|.
 1njzA   384 DIHTKTAMDIF--QVSEDEVTP--------NMRRQAKAVNFGIVYGISDYGLAQNLNISRKEAAEFIERYFESFPG  449

DNAP01  1091 FKKFVEDNIAELSENFGFRYLPIFNHRFYIGNPYHSIKQKG------LNYIIQNLSSSLTAYTAYALYDDLRTNY 1161
             .++|.++-+++..++--.+  .++..|.|+-+--.-....++      +|++||.-++.+.-..-..++..+ ...
 1njzA   450 VKRYMENIVQEAKQKGYVT--TLLHRRRYLPDITSRNFNVRSFAERMAMNTPIQGSAADIIKKAMIDLNARL-KEE  548

                      Motif C
DNAP01  1162 GVEIQLLGFVHDAIEFEFDAKDLFIILDRMNYWYKEMPLEKWDIPSDFDFELGSS 1215
             |....++-.|||.+-||-+...+--...-+..--.+-.  ++++|-..+.+.|.|
 1njzA   549 RLQAHLLLQVHDELILEAPKEEMERLCRLVPEVMEQAV--TLRVPLKVDYHYGST  575
```

Figure S9. *At the top, the cartoon representation of the modelled C-terminus (aa residues 946-1215) of the YerA41 DNAP01. The lower part shows the DNA fragment imbibed from 1NJZ stucture. The secondary structure elements have been colored differently (α-helix: red; β-sheet: yellow, and loop: green). At the bottom, the amino acid sequence alignment between DNAP01-Ct and 1NJZ_A.*

```
YerA41_DNAP02   MQLSKVDVYDASHLTYRMYHQAK-SINPENPIQT-ASYLLLNSVFALMKGN---KGIPTVFLYDTAG-ATKDRKEIDENYKSNRVFNP-YVHELKTRMTRYLTSLGFPVVGIEGLEADDI
DNAP of GPB_I   -MKDRIFVIDGSSYLYRAYHAMP-PLSTSKGQPTGAVKGVTNMLMNLKKDS---EGSPIIVVFDAKG-KTF-RNDIYSEYKANRPPMPDELRLQLQPVKSICKAIGFPLIEIEGVEADDV
DNAP of T_afr   -M-GKMFLFDGTGLVYRAFYAIDQSLQTSSGLHTNAVYGLTKMLIKFLKEHISIGKDACVFVLDSKGGSKK-RKDILETYKANRPSTPDLLLEQIPYVEELVEALGIKVLKIEGFEADDI
DNAP of T_glo   -M-GKMFLFDGTGLVYRAFYAIDQSLQTSSGLHTNAVYGLTKMLIKFLKEHISIGKDACVFVLDSKGGSKK-RKDILETYKANRPSTPDLLLEQIPYVEELVEALGIKVLKIEGFEADDI
DNAP of SAR86A  -MKDRIFVIDGSSYLYRAYHAMP-PLSTSKGQPTGAVKGVTNMLMNLKKDS---EGSPIIVVFDAKG-KTF-RNDIYSEYKANRPPMPDELRLQLQPVKSICKAIGFPLIEIEGVEADDV
DNAP of T_jap   -M-GKMFLFDGTGLVYRAFYAIDQSLQTSSGLHTNAVYGLTKMLIKFLKEHINIGKDACVFVLDSKGGSKK-RKNILETYKANRPSTPDLLLEQIPYVEEFVDALGIKVLKIEGFEADDI
DNAP of GPB_II  -MKDRIFVIDGSSYLYRAFHAMP-SLTTSSGKPTGAVKGVTNMLINLKKDS---EGSPIIIVFDAKG-KTF-RNEIYSEYKANRPPMPEELREQLKPVKSICKAIGFPLIEIEGVEADDV
                         *     **                * *            *              *  *     *  *   ** **   *                 *     *** ****

YerA41_DNAP02   AYYLSR---NCLNPLKFISEDKDWYSYVIDNKELLRPIRDKQLAGGSLTTFNDLLMKVDPILSGYLPENNTKEDIDREVHKVWIQRKALLGDASDNVAKFKGVGKSNIVQLLVKY-NKAG
DNAP of GPB_I   IATITKMAKDAKYKCVVSSLDKDLMQLVEDPDTTLM---N---------TMKHEIFNEDKVFEKF----GVKPSQIRDM-------LALVGDSSDNIPGVPKVGQKTAAKWLNEYGNLEG
DNAP of T_afr   IATLSKKFENDFEKVNIITGDKDLLQLVSDKVFVWRVERG---------ITDLVLYDRNKVLEKY----GISPEQFKDY-------LSLVGDQIDNIPGVKGIGKKTAVSLLKKYNSLEN
DNAP of T_glo   IATLSKKFENDFEKVNIITGDKDLLQLVSDKVFVWRVERG---------ITDLVLYDRNKVLEKY----GIYPEQFKDY-------LSLVGDQIDNIPGVKGIGKKTAVSLLKKYNSLEN
DNAP of SAR86A  IATITKMAKDAKYKCVVSSLDKDLMQLVEDPDTTLM---N---------TMKHEIFNEDKVFEKF----GVKPSQIRDM-------LALVGDSSDNIPGVSKVGQKTAAKWLNEYGNLEG
DNAP of T_jap   IATLSKKFENDFEKVNIITGDKDLLQLISDKVFVWRVERG---------ITDLVLYDRNKVFEKY----GIYPEQFKDY-------LSLVGDQIDNIPGVKGIGKKTAVSLLKKYNSLEN
DNAP of GPB_II  ISTIALLAKKAKYKCVISSLDKDLMQLVEDPHITMM---D---------TMKHKIFNEEGVFEKF----GVKPSQIRDM-------LALVGDASDNIPGIPKVGQKTAAKWLAEYGNLEN
                                    ***      *                                                          * **  **       *       *  *

YerA41_DNAP02   CDINKFTPKGVVEKRFKADHDRFLRNIQVLSYDRMQDMDIKDQLTFEFNPDIDIKAALLEIAKEINSSRITNNADKYAQRILDNHVPVVFNWK--------------------------
DNAP of GPB_I   IISNADSIKGVVGDNLRNSLDDLDRNIELVSLKXDVDLNLKXEDLLIFNPDEE---ELEKIFNELEFAAINNNKDEKSPKKLGKYKTVLDKKD-LDDWIKKIKNSKAFAIDTETDSVDTV
DNAP of T_afr   VLNNINLLTEKLRRLLEDSKEDLQKSIELVELIYDVPMDVEKDEIIYRGYNPD---KLLKVLKKYEFSSII--KELNLQEKLEKEYILVDNEDKLKKLADEIEKYKTFSIDTETTSLDPF
DNAP of T_glo   VLKNINLLTEKLRRLLENSKEDLQKSVELVELIYDVPVDVEKDEIIYRGYNPD---KLLKVLKKYEFSSII--KELNLQEKLEKKYILVDNEDKLKKLAEEIEKYKTFSIDTETTSLDPF
DNAP of SAR86A  IISNADSIKGVVGDNLRNSLDDLDRNIELVSLKEDVDLNLKFEDLLICNPDEE---ELEKIFNELEFAAINNNKDEKSPKKLGKYKTVLDKKD-LDDWIKKIKNSKAFAIDTETDSVDTV
DNAP of T_jap   VLKNINLLTEKLRRLLENSKEDLQKSVELVELIYDVPVDLEKDEIIYRGYNSD---KLLKVLKKYEFSSII--KELNLQEKLEKEYILVDNEDKLKKLADEIEKYKTFSIDTETTSLDPF
DNAP of GPB_II  IKKNAESIKGVVGENLRNTINDLDRNIELVSLKQNVDLSMKFEDLLVFSPDDE---KLEEIFSELEFKALDSKKEKEIPKEKSNYETILDQKS-LKGWISKINNCETFAIDTETDSVNTV
                   *                                                     *

YerA41_DNAP02   ------------------------------------------------------------------------------------------------------------------------
DNAP of GPB_I   TANLLGISISVNENEGCYXPLGHTYEDCPKQLSMDYVVKTLGSVIEENQNKAVGQNLKFDIPILSRHGINITNFLADTMLMSYVLNSTATRHGMDRLAEYYLNYTTTKYTDVTGTASKQI
DNAP of T_afr   EAKLVGISISTMEGKAYYIPVSHFGA---KNISKGSVVKFLKQILQEKDYNIVGQNLKFDYEIFKSMGFSPNVPHFDTMIAAYLLNPDEKRFNLEELSLKYLGYKMISFDELVNENVPLF
DNAP of T_glo   EAKLVGVSISTMEGKAYYIPVSHFGA---KNISKSLIDKFLKQILQEKDYNIVGQNLKFDYEIFKSMGISPNIPHFDTMIAAYLLNPDEKRFNLEELSLKYLGYKMISFDELVNENVPLF
DNAP of SAR86A  TANLLGISISVNENEGCYIPLGHTYEDCPKQLSMDYVIKTLGSVIEENQNKAVGQNLKFDIPILSRHGINITNFLADTMLMSYVLNSTATRHGMDRLAEYYLNYTTTKYTDVTGTASKQI
DNAP of T_jap   EAKLVGVSISTMEGKAYYIPVSHFGA---KNISKSLIDKFLKQILQEKDYNIVGQNLKFDYEIFKSMGFSPNIPHFDTMIAAYLLNPDEKRFNLEELSLKYLGYKMIAFDELVNENVPLF
DNAP of GPB_II  SANLIGISLAVTEKEGCYIPIAHDYEDCPNQLGIDYIVKNLGPVIEKNQEKAIGQNLKFDIPILERHGIKITKFHADTMLMSYVLNSTATRHGMDRLADYYLNYSTTKYSDVTGTASKQI
```

Figure S10.*Multiple sequence alignment of DNAP02 with N-terminal parts of best BLASTp hits.* ***DNAP of GPB_I,*** *DNAP of a Gammaproteobacteria (MAN84441.1);* ***DNAP of T_afr,*** *DNAP I of* Thermosipho africanus *(WP_004103743.1);* ***DNAP of T_glo,*** *DNAP I of* Thermosipho globiformans *(WP_126993006.1);* ***DNAP of SAR86A,*** *DNAP of SAR86 cluster bacteria SAR86A (EJP71381.1);* ***DNAP of T_jap,*** *DNAP I of* Thermosipho japonicas *(WP_184619851.1);* ***DNAP of GPB_II****, DNAP I of Gammaproteobacteria (MBL32466.1). Note that DNAP02 sequence aligns to the N-terminal 5'-3' exonuclease domains of these typical 900 aa DNAPs, and not to the C-terminal domains that typically carry the polymerase activity.*

```
YerA41_DNAP03  MKVLDKSYVVYYLNRLAKKYPLP------NHKYYAFTNAASAIA--QSDIKKIDNEVIHNELKKVKFVGPFIKEILTMLVNEDID---EKYVLENTDNLIDCYELRDVFPVLIEKYY---
PolX_Hal_prae  ---MKKINNKEIAKILSEFADLMAIKGENDFKIKAYTNAARQIESYQTKISELVAT---NELKEIKGIGSGIAETITELLENGSISEMEAIKAELPPGVIEMTNIQGLGPKTAHRFYYEL
PolX_Meth_ar   ---MKNALVAKILNRVADFLE---LK-EETFRVAYRRAAHTIQLLPTDIEEYAKK---RKLTELPGIGRNIAEKVEEILETGRLSYLEKLEKEYPIDMDSLLAIEGIGPKTIKILYEKL
PolX_Meth_sp   ---MKNALVARILNRVADFLE---LK-EETFRAKAYRRAAHTIQLLPTDIEEYAKK---RKLTELPGIGKNIAEKIEEILETGQLSYLEELEKEYPIDMDSLLAIEGIGPRTIKILYEKL
PolX_Hal_sac   ---MQEMSNKEIAKILAEFADLMAIKGENDFKIRAYTNAARKIESYPEDIFELAEE---DKLKEIKGIGSGIAESITELLQNGIIEEMEAVKAELPPGVIEMTDIQGLGPKTAHRFYYEL
PolX_Therm     ---MKNHEIAYILYEIANMLE---IKGENFFKIRAYRNAAHEIENLAIDIQKLVNQ---NALKEIDGVGEAISGKIYEILEKGTCRYYEELKAEVPRGLVDMLKIPGLGAKRIKVIYDNL
PolX_Planct    ---MQKEQVIVNLKHIATLME---LKGENSFKIRAFHNACRALESETENLKTIVDE---NRLTNIKGIGEGIAKKIAEMVLHDKSTWLEDLQNEIPEDLVKMLEIPGLGAKKVRTIHKEL
                                                 *   *                      *      *  *                *    *

                                                                                                  PolX motif I
YerA41_DNAP03  ----------------------LTGIGELKS-----ILKVNQSVLDLLNDKHLEFLNLNRSKDWVSLIATYLDNPKIRIAGSMRRGCLVIKDFDLVTYLTYPE-LRDYIASVNNSTNDIR
PolX_Hal_prae  EIEDLERLEKAL---QTGKIQKLKGFGKKSEAKLLKALKNHEKYVDKINLNKALKTANQIIKKIKHQVDS-SLFSQIEVCGSSRRAKEMTGDLDILIATSQPEAIAAKLKNLDFTAE---
PolX_Meth_ar   ---KIKNLQDLEYHARRGDLRKIKGIGEKKEER----ILQSIEFVRSTLGRRLLAYAEPIAEYIKSLIEGHPAAERVEIAGSIRRGGETIGDIDILTTTKKPKEIIDYFTSLEVADE---
PolX_Meth_sp   ---KIKNLQDLEYHAQKGNLQKIKGIGEKKEKR----ILQSIKFVRSTLGRRLLAYAAPIAEYIKSRIEEHPKTEKVKIAGSIRRGKETIGDIDILTTTKNPKEIIDYFTSLEIADE---
PolX_Hal_sac   EIVDLISLEKAL---KEGRVQELKGFGKKSEEKLLKALKNHEKYVDKINLAQALKTAGEIIETIKTELEP-DFFEEIEICGSSRRAKELTGDLDILIATAKPEKLSQKLKNLNFTAE---
PolX_Therm     ---KIASIEELEKAANSKQLQTLPGIGVKTEQS----IIRGIQMIKGRAGRYLLSTALSIAQDMVTRLSSLPSVEKAQIAGSLRRKKEMVGDIDLLVVSNSHQQVTESFLKFPEIRE---
PolX_Planct    ---GITTLEELKLACVDNKIESLPKFGAKVQQN----ILTGIETLKKHAGRYLFSFAHAESQKIIQYMRECECISKLEIAGSLRRRKEVTKDIDMLVVASDAETVMQHFVSYPEVEK---
                                         *                                                     ** **      * *

                                      PolX motif II
YerA41_DNAP03  INIIGNGKKRVRINLINNLTEKSIEGDVRIVNEESIHSAMLYFTGPKSLNVKMRGIAKSKGLTLNEYGIVGT-DKKLITFNSEEEIFKYLGMKYLAPTNRK-------------------
PolX_Hal_prae  --VIGAGKTKVSIRT-----EAGVQTDFRLVSQAEFPSALHYFTGSKAHNVRMRQLAKNNNLKLSEYGLFKK-DGSKKLIESEADIFSILGLDYIIPELREDEGEIEAAQKGELPTSIEL
PolX_Meth_ar   --IIAKGTKKAIIRL-----EDGLECELRIFREEEFGAALLYFTGSMEFNVELRKLARSKSMKLNEYGLYK--NGKRIASKTEKEIFKALGLEYIQPELRENNGEIEAAARGNLPSLIRE
PolX_Meth_sp   --IIAKGPKKAIIRL-----EDGLECELRIFKEDEFGAALLYFTGSMEFNVELRRLARSKSMKLNEYGLYK--NSKRIASKTEKEIFKALGLEYIQPELRENNGEIEAALQGKLPTLVRE
PolX_Hal_sac   --VIGAGATKVSIRT-----EQGVQTDFRLVTKEEFPSALHYFTGSKAHNVRMRQLAKEHNLKLSEYGLFRD-DDSKEKIESEADIFKILGLEYIIPELREDQGEIEAAQKGELPDSIKL
PolX_Therm     --VIAKGSTKTSVIL-----ELGIQVDLRVVKPESFSAALLHFTGSKEHNTKLRSLALRKGLRLNEYGIINLDDGEIIYPQSEKEIYKELGMPYIIPEIREDTGEVEAAMKDSLPNSVNL
PolX_Planct    --IIGHGPKKSSIQL-----RKGLQVDLRVVEEHQFAFALMYFTGSKEHNTQMRSIAKKHGLKLNEYGLFDE-NDKSAVCESEEQVFSALGLQYVIPELRENLGEIELAQEKKLPKLVSQ
                  *  *                     *         *   ***    *   *  *      * ***              *      **  *  *  *
```

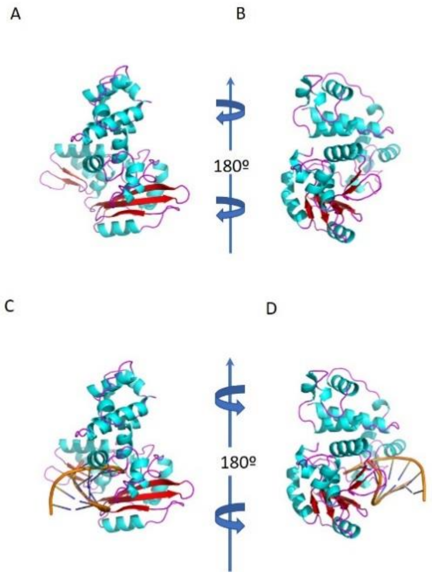


```
DNAP03      6 KSYVVYYLNRLAKKYPLPNH---KYYAFTNAASAIAQSDIKKIDNEVIHNELKKVKFVGP   62
              |++|+++|++||+++++.++   |++||++||++|+++|.++    .+.+++.+|||||+
8ICZ        4 NGGITDMLTELANFEKNVSQAIHKYNAYRKAASVIAKYPHKI----KSGAEAKKLPGVGT   59

DNAP03     63 FIKEILTMLVNEDI---DEKYVLENTDNLIDCY-ELRDVFPVLIEKYYLTGIGELKSILK  118
              +|+++|.||++||+   +++++.+.|++++.++ +||||||++|++||..|++||+||.+
8ICZ       60 KIAEKIDEFLATGKLRKLEKIRQDDTSSSINFLTRVSGIGPSAARKFVDEGIKTLEDLRK  119

DNAP03    119 V--NQSVLDLLNDKHLEFLNLNRSKDWVSLIATYL--------DNPKIRIAGSMRRGCLV  168
              .  +....+.++.++++.+...+++..+..+...+        .+++|++|||||||+++
8ICZ      120 NEDKLNHHQRIGLKYFGDFEKRIPREEMLQMQDIVLNEVKKVDSEYIATVCGSFRRGAES  179

DNAP03    169 IKDFDLVTYLTYP---------ELRDYIASVNNSTNDIRINIIGNGKKRVRINLINNLT-  218
              ||||||||+++++         .+...+..+..................+.+...+... 
8ICZ      180 SGDMDVLLTHPSFTSESTKQPKLLHQVVEQLQKVHFITDTLSKGETKFMGVCQLPSKNDE  239

DNAP03    219 --EKSIEGDVRIVNEESIHSAMLYFTGPKSLNVKMRGIAKSKGLTLNEYGIVG-----TD  271
                +.|+||||++|||++||+||+|||||++||++||.+|+++||+||+||||+     .+
8ICZ      240 KEYPHRRIDIRLIPKDQYYCGVLYFTGSDIFNKNMRAHALEKGFTINEYTIRPLGVTGVA  299

DNAP03    272 KKLITFNSEEEIFKYLGMKYLAPTNRK  298
              +..++++||++||++|||||||||+||
8ICZ      300 GEPLPVDSEKDIFDYIQWKYREPKDRS  326
```

Figure S11. *Characteristics of DNAP03 showing similarity to DNA polymerase beta.*

TOP PANEL. *Multiple sequence alignment of YerA41 DNAP03 with N-terminal polymerase domains of nearest PolX hits in BLASTp. The presence of the conserved PolX motifs I and II are indicated.* ***PolX_Hal_prae,*** *DNAP PolX of Halanaerobium praevalens (WP_014553009.1);* ***PolX_Meth_ar,*** *PHP domain protein of Methanobacteriaceae archaeon (KUK00968.1);* ***PolX_Meth_sp,*** *DNAP PolX of Methanothermobacter sp. (HHW15868.1);* ***PolX_Hal_sac****, DNAP PolX of Halanaerobium saccharolyticum (WP_108142115.1);* ***PolX_Therm,*** *DNAP PolX of Thermoanaerobacterales bacterium (HHY04536.1);* ***PolX_Planct,*** *DNAP PolX of Planctomycetes bacterium SRT547 (BBM81848.1).*

MIDDLE PANEL. *The figures A and B shows the cartoon representation of the modelled DNAP03, and the figures C and D show the model with the DNA fragment imbibed from 8ICZ structure of human DNA polymerase beta. The secondary structure elements have been colored differently (Helix: cyan; Sheet: red, and Loop: pink).*

BOTTOM PANEL. *The amino acid sequence alignment between DNAP03 and 8ICZ with the aligned PolX motifs shown in bold.*

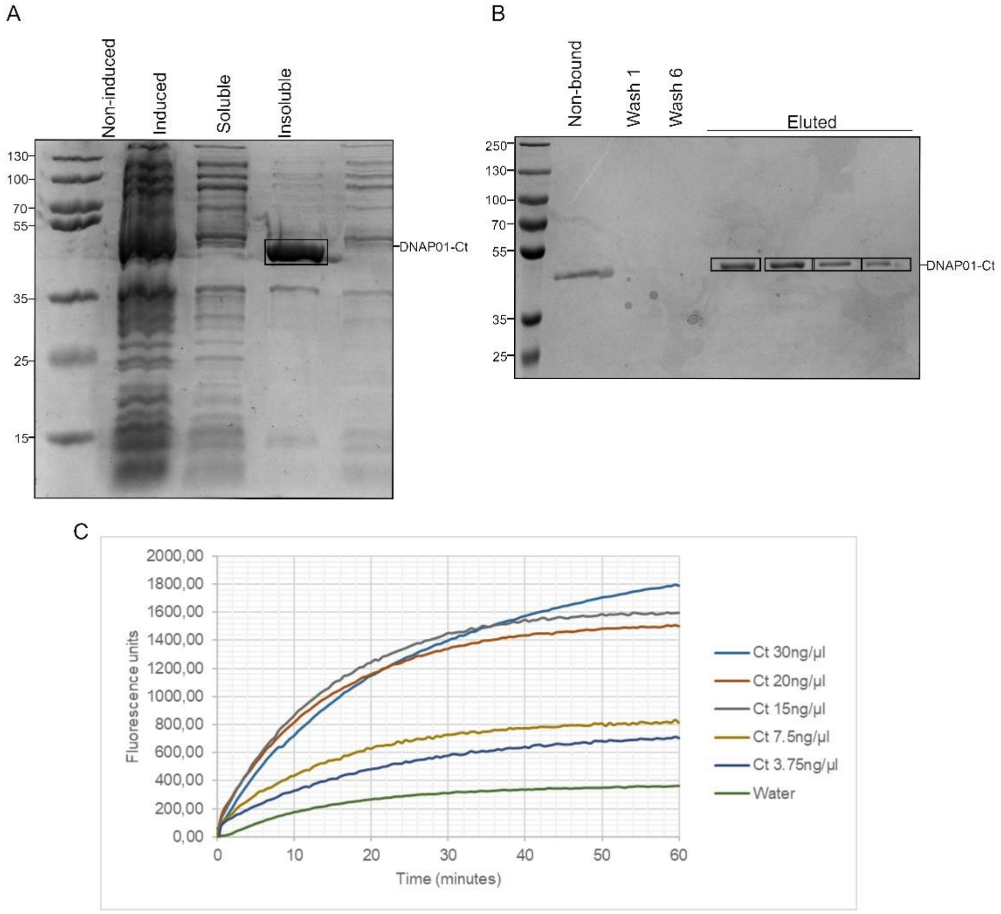

Figure S12. *Isolation and purification of DNAP01-Ct.* ***Panel A****, SDS-PAGE of protein solubility determination for the induced E. coli bacteria.* ***Panel B****, SDS-PAGE of DNAP01-Ct purification under native conditions. The molecular weight sizes of the ladder bands are given in kDa.* ***Panel C,*** *DNA polymerase activity of DNAP01-Ct, measured with a commercial assay for 60 min. Each line represents the mean of three replicates. Negative control is represented by water.*

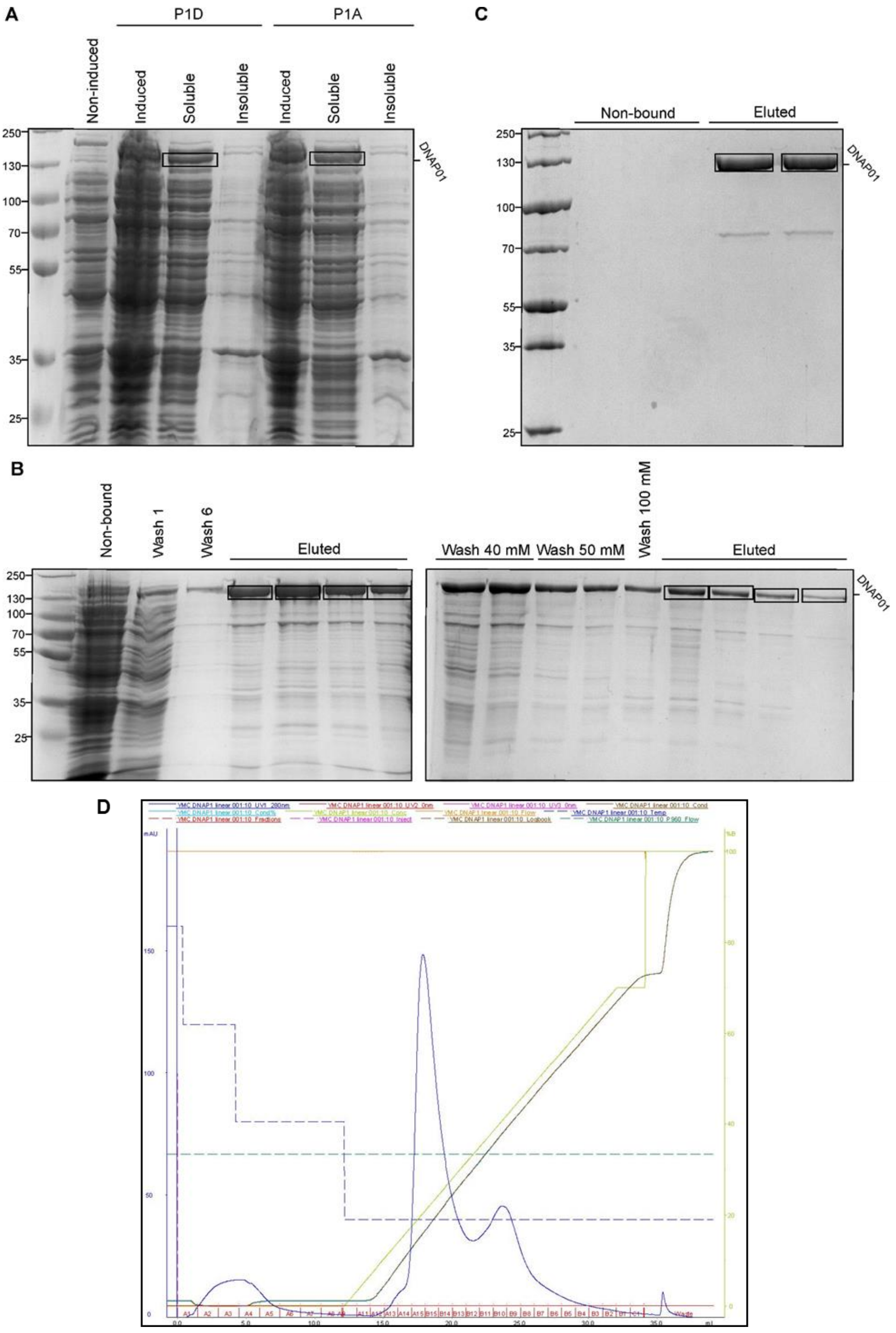

Figure S13. *Isolation and purification of DNAP01.* ***Panel A****, SDS-PAGE of protein solubility determination for the induced P1A and P1D clones.* ***Panel B****, SDS-PAGE of DNAP01 purification under native conditions.* ***Left****: purification following typical method.* ***Right****: purification with increasing imidazole concentration in the washes; molarity of imidazole is indicated. The molecular weight sizes of the ladder bands are given in* kDa. ***Panel C****, SDS-PAGE of two non-bound and eluted fractions from Anion Exchange Chromatography purification of DNAP01.* ***Panel D****, Anion Exchange chromatogram to purify His-tagged DNAP01.*

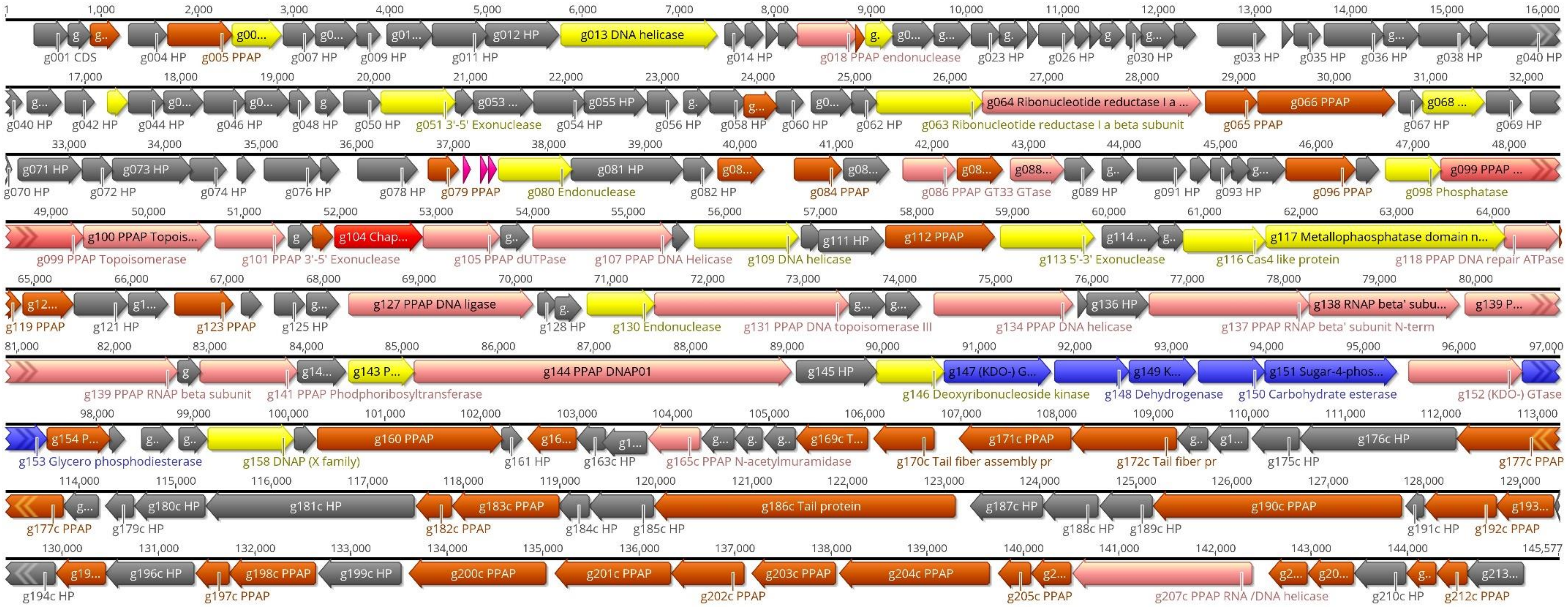

Figure S14. *The complete DNA genome of phage YerA41. The predicted genes are shown as colored arrows labelled with predicted functions. Phage particle associated proteins (PPAP) predicted as structural proteins, brown; PPAPs with enzymatic function, pink; sugar modification enzymes, blue; nucleic acid modification enzymes, yellow; hypothetical proteins, grey; tRNA genes, lila; predicted promoters, numbered and green; carbohydrate interactions, yellow. The map was drawn with Geneious 10.2.6 (www.geneious.com). HP, hypothetical protein. The annotated sequence has been submitted to DNA databases under accession number MW570730.*

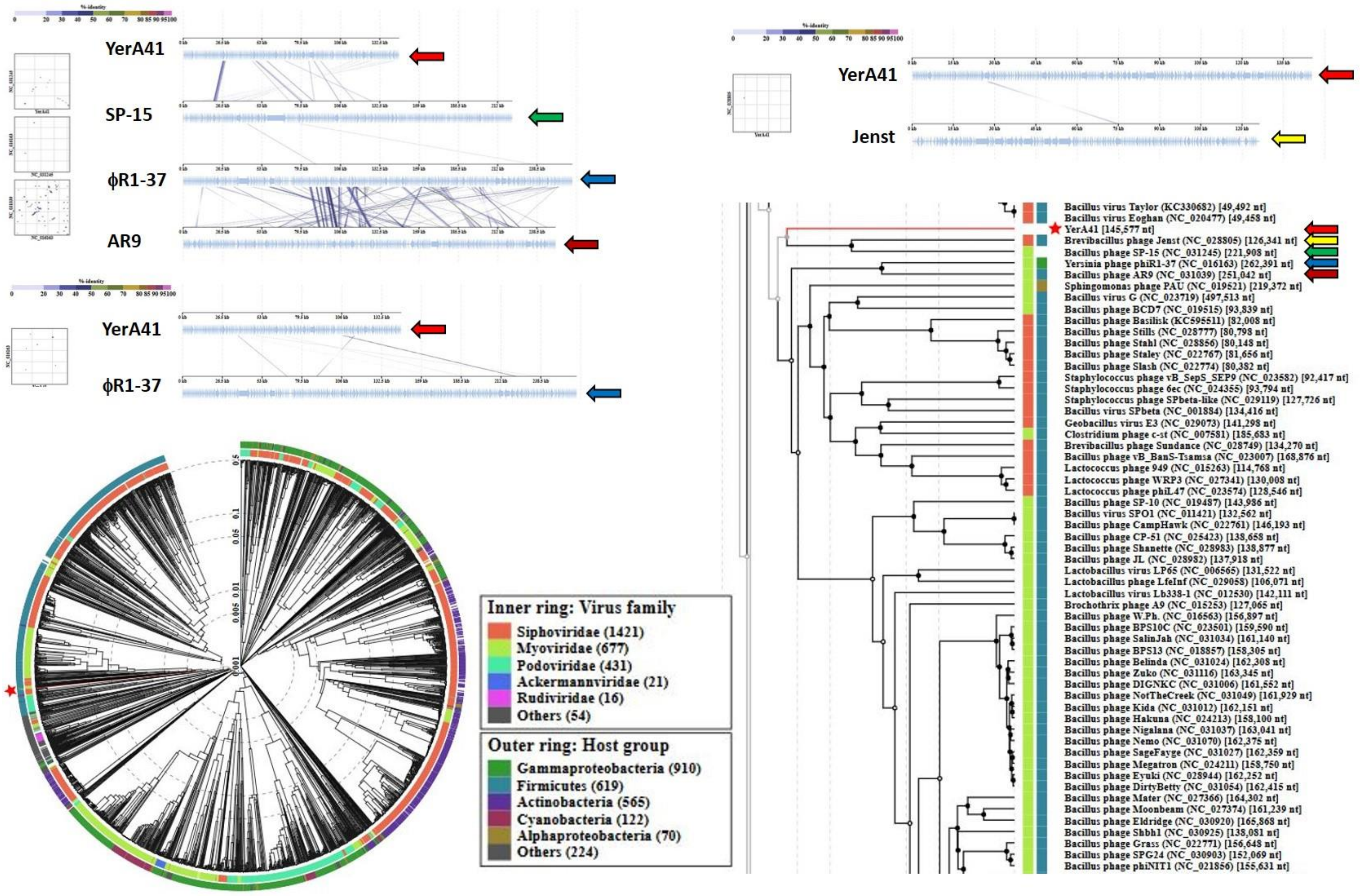

Figure S15. *Position of YerA41 in the Phage Proteomic Tree generated by VIPTree (https://www.genome.jp/viptree/, visited on 7.4.2021). At bottom left, a circular proteomic tree of prokaryotic dsDNA viruses colored by indicated virus families and host taxonomic groups. At bottom right, part of the rectangular presentation of the proteomic tree showing the closest related phages to YerA41. The location of YerA41 in both is indicated by the red asterix. At the top, genomic alignments of YerA41 and the closest related phages Brevibacillus phage Jenst (NC_028805, yellow arrow), Bacillus phage SP-15 (NC_031245, green arrow), Yersinia phage* *R1-37 (NC_016163, blue arrow), and Bacillus phage AR9 (NC031039, brown arrow).*

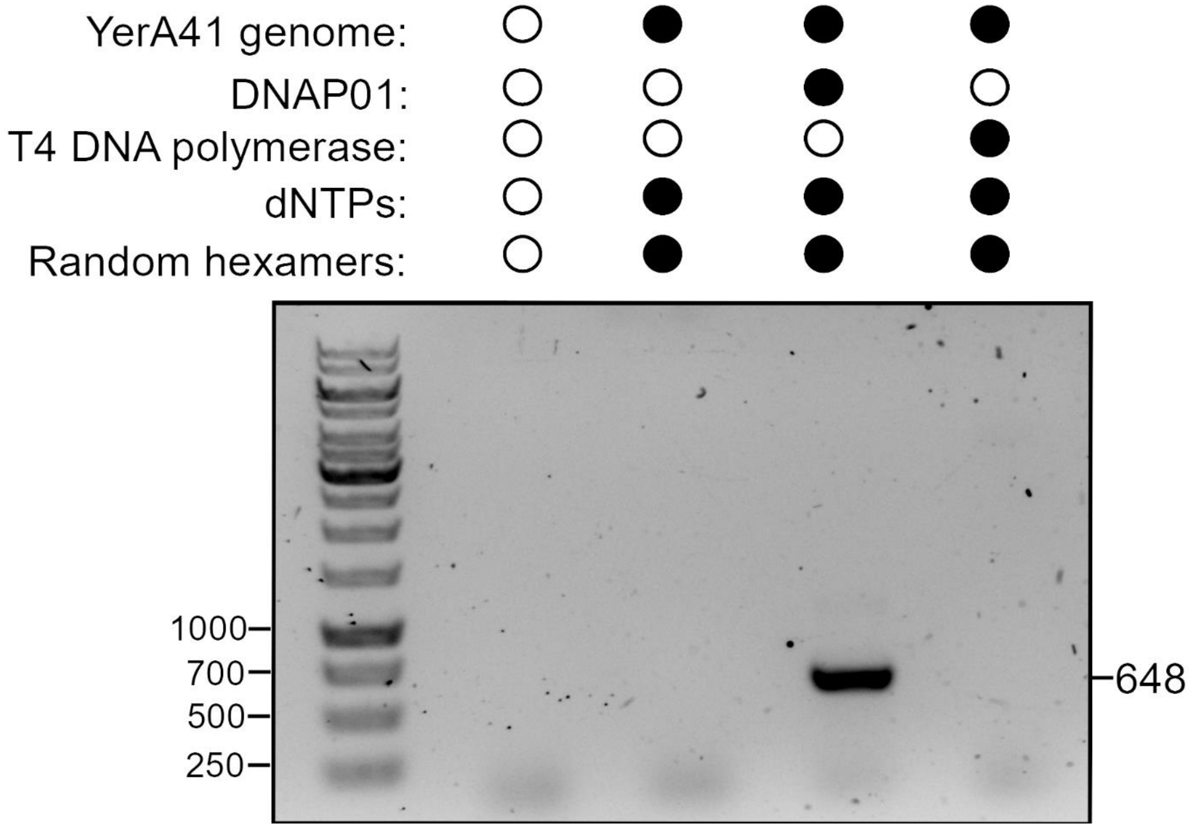

Figure S16. *T4 DNA polymerase cannot replace DNAP01 in the assay. The obtained PCR products were analysed in 1% agarose gel electrophoresis. Above the gel image are indicated the added reagents and any special conditions applied to the DNAP01 incubation. Aliquots of the samples were used as templates for PCR with Dream-taq using gp8 primers, as described in the methods section of the main text. The sizes of the bands are given in bp.*
